# Delivering infectious disease interventions to women and children in conflict settings: a systematic review

**DOI:** 10.1136/bmjgh-2019-001967

**Published:** 2020-04-26

**Authors:** Sarah Meteke, Marianne Stefopulos, Daina Als, Michelle F Gaffey, Mahdis Kamali, Fahad J Siddiqui, Mariella Munyuzangabo, Reena P Jain, Shailja Shah, Amruta Radhakrishnan, Anushka Ataullahjan, Zulfiqar A Bhutta

**Affiliations:** 1 Centre for Global Child Health, Hospital for Sick Children, Toronto, Ontario, Canada; 2 Health System and Services Research, Duke-NUS Medical School, Singapore; 3 Center of Excellence in Women and Child Health, Aga Khan University, Karachi, Pakistan

**Keywords:** child health, systematic review, vaccines, infections, diseases, disorders, injuries, maternal health

## Abstract

**Background:**

Conflict has played a role in the large-scale deterioration of health systems in low-income and middle-income countries (LMICs) and increased risk of infections and outbreaks. This systematic review aimed to synthesise the literature on mechanisms of delivery for a range of infectious disease-related interventions provided to conflict-affected women, children and adolescents.

**Methods:**

We searched Medline, Embase, CINAHL and PsychINFO databases for literature published in English from January 1990 to March 2018. Eligible publications reported on conflict-affected neonates, children, adolescents or women in LMICs who received an infectious disease intervention. We extracted and synthesised information on delivery characteristics, including delivery site and personnel involved, as well as barriers and facilitators, and we tabulated reported intervention coverage and effectiveness data.

**Results:**

A majority of the 194 eligible publications reported on intervention delivery in sub-Saharan Africa. Vaccines for measles and polio were the most commonly reported interventions, followed by malaria treatment. Over two-thirds of reported interventions were delivered in camp settings for displaced families. The use of clinics as a delivery site was reported across all intervention types, but outreach and community-based delivery were also reported for many interventions. Key barriers to service delivery included restricted access to target populations; conversely, adopting social mobilisation strategies and collaborating with community figures were reported as facilitating intervention delivery. Few publications reported on intervention coverage, mostly reporting variable coverage for vaccines, and fewer reported on intervention effectiveness, mostly for malaria treatment regimens.

**Conclusions:**

Despite an increased focus on health outcomes in humanitarian crises, our review highlights important gaps in the literature on intervention delivery among specific subpopulations and geographies. This indicates a need for more rigorous research and reporting on effective strategies for delivering infectious disease interventions in different conflict contexts.

**PROSPERO registration number:**

CRD42019125221.

**Key questions**

**What is already known?**

- Conflict has especially devastating consequences for the implementation and delivery of health interventions for women, children and adolescents, including infectious disease interventions.
- Often, there are resurgences of infectious diseases such as HIV transmission, polio, measles and other vaccine-preventable diseases due to severed access to standard prevention and treatment services.

**What are the new findings?**

- In many conflict settings, coverage rates of infectious disease interventions are challenging to ascertain and are often unknown.
- Health workers, and the infrastructure and systems that support them, are debilitated by conflict. Prioritising local communities’ needs and working collaboratively with multiple sectors to develop holistic and sustainable solutions that address security concerns is crucial in restoring operational functions to deliver essential infectious disease services.

**What do the new findings imply?**

- High-quality research is required to identify evidence-based infectious disease interventions to further support its provision and uptake in conflict-affected settings.
- There must be a greater focus on enhancing the availability of health data, as it relates to infectious diseases, of women and children in conflict settings. Accessible and timely data will encourage accountability and aid in the coordination of multiple actors across country, regional and global levels.

## Introduction

Significant gaps in access to infectious disease interventions within health systems exist globally and are further impeded by war, civil unrest and political violence.^1^ The populations most vulnerable to infectious disease transmission are those who are relegated to live in crowded conditions with inadequate shelter, poor sanitation, insufficient water quality and quantity, limited food security and a lack of access to essential healthcare services.^2^ These risk factors are major contextual determinants of child malnutrition, outbreaks of communicable diseases and low immunisation coverage in many low-income and middle-income countries (LMICs).^3^ Damage to sanitation facilities or a scarcity of clean water means that water-borne and vector-borne diseases are transmitted rapidly. Moreover, conflict-affected countries are potential zones of new emergence such as the case of Ebola imported into the Democratic Republic of Congo (DRC), and conflict often triggers the resurgence of previously controlled vaccine-preventable diseases (VPD) due to severed access to standard prevention and treatment services.^4 5^ For instance, despite fairly high preconflict VPD vaccination coverage rates in Syria, reports of measles outbreaks and acute flaccid paralysis related to poliovirus have become increasingly common.^6^ UNICEF figures show that in Syria, routine administration of first-dose measles-containing-vaccine decreased from 82% coverage in 2010 to 54% in 2014.^7^ Beyond hindering the provision of routine immunisations and treatment of acute conditions, the breakdown of a health system and restricted access to medical supplies can also disrupt the continuum of care for long-term treatment courses such as for HIV or tuberculosis (TB).^1^

Similar to the case in LMICs where armed conflict is absent, acute respiratory infections (ARI, most notably pneumonia), TB, diarrhoea, measles and malaria account for the highest morbidity among children in crisis-affected populations.^8^ However, a lack of consensus on effective intervention implementation strategies and disrupted routine surveillance make it difficult to coordinate sound public health responses in such settings.^9^ To address this difficulty and help improve the quality of humanitarian response, a group of non-governmental organisations (NGOs) and the Red Cross and Red Crescent Movement collectively developed a humanitarian charter and a set of minimum standards as part of the *Sphere Project* initiated in 1997. Now in its fourth edition, the *Sphere Handbook* outlines minimum standards for humanitarian response across a range of sectors, including WASH, shelter, health and nutrition. It also outlines key actions for controlling infectious diseases in emergency settings: (1) swiftly implementing prevention measures; (2) establishing surveillance and reporting systems; (3) active diagnosis and case management and (4) outbreak preparedness and response in a timely and efficient manner.^10^ However, the *Sphere Handbook* does not sufficiently address how best to implement such actions, and there is little evidence-based guidance on implementation available elsewhere.^10^

Stronger scientific evidence on implementing effective infectious disease interventions is needed to effectively respond to humanitarian crises. Previous reviews have systematically characterised the evidence on the effectiveness of infectious disease interventions during humanitarian crises.^11 12^ Though there has been increased international focus to produce and disseminate systematic and comprehensive guidelines for the delivery of infectious disease services in conflict settings, significant challenges remain in terms of provision and utilisation of critical health interventions.^13^ This paper is part of a set of eight systematic reviews using a common protocol to examine the delivery of reproductive, maternal, newborn, child and adolescent health and nutrition interventions in conflict settings, motivated by the urgent need to better address the vulnerabilities of women and children in humanitarian crises generally and in conflict settings particularly.^14 15^ Here we aimed to systematically characterise the literature on the delivery of infectious disease interventions to women, children and adolescents and identify key gaps in that literature; and to synthesise reported information on intervention delivery characteristics, including how they may vary by setting and by population displacement status.

## Methods

### Search strategy

A systematic search of indexed literature published from 1 January 1990 to 31 March 2018 was conducted in Medline, Embase, CINAHL and PsychINFO databases using OVID and EBSCO interfaces. The ‘Explode’ function for the Medical Subject Heading Terms was activated for main sets of terms related to three concepts: (a) conflict; (b) women and children and (c) infectious diseases. Conflict-related search terms included war, crisis, refugees, internally displaced person (IDP) and stateless. Population-related terms included newborn, children, adolescents, women and pregnant. Infectious-disease-related terms included immunisation, cholera, measles, HIV, TB and other disease terms. The complete search syntax for Medline is presented in online supplementary appendix A. We also screened reference lists of key systematic reviews conducted previously in the field of humanitarian health.

10.1136/bmjgh-2019-001967.supp1 Supplementary data

For grey literature, we searched the websites of 10 major humanitarian agencies and organisations that are actively involved in responding to or researching conflict situations for reports on the delivery of health interventions in our population of interest. These websites included Emergency Nutrition Network, International Committee of the Red Cross, International Rescue Committee, Médecins Sans Frontières, Save the Children, United Nations Population Fund, United Nations High Commissioner for Refugees, United Nations Children’s Fund, Women’s Refugee Commission and World Vision. We used broad terms for conflict and health interventions tailored to the search functionality of each website. Grey literature documents published from 1 January 2013 to 30 November 2018 were reviewed.

### Eligibility criteria

Eligible publications were limited to those reporting on populations affected by conflict in LMICs, as classified by the World Bank.^16^ The intervention must have targeted or included neonates, children, adolescents or women of reproductive age. The publication must have also described an intervention being delivered during or within 5 years of cessation of conflict. A 5-year cut-off was applied to help ensure that we were capturing humanitarian rather than broader development contexts, and assuming that it may take recovering LMICs up to 5 years to be able to use foreign aid impactfully.^17^ If ongoing or recent cessation of conflict was not referred to explicitly in the publication, we consulted online encyclopaedic sources as well as the UN Office for the Coordination of Humanitarian Affairs website for information on conflict duration, to assess whether the reported time period of intervention delivery met the 5-year cut-off criterion. To identify the most informative resources from the large volume of grey literature that we retrieved even using a shorter publication window, the same population and intervention delivery period criteria used for the indexed literature were applied, along with an additional requirement of explicit reporting in the grey literature on the delivery site and personnel for each intervention.

Non-English publications; case reports of single patients; publications reporting on military personnel, refugee populations bound for a high-income country, surgical techniques, economic or mathematical modelling; and editorials and opinion pieces were excluded from our review. Other exclusion criteria included systematic reviews, guidelines and publications where no specific health intervention was described (eg, prevalence studies).

### Data extraction and analysis

All identified indexed records were downloaded into EndNote software and duplicates were removed. Unique records were then imported into Covidence software for screening. Titles and abstracts were screened for relevance in duplicate, and the full-text of each potentially relevant publication was then screened by a single reviewer who noted reasons for exclusion at this stage.

Relevant information and data from the eligible indexed and grey literature were extracted in duplicate into a customised and piloted form using REDCap software. Key variables included publication author, publication year, study design, setting, target population characteristics, intervention characteristics and delivery characteristics, including any author-reported narrative information on delivery barriers or facilitators. We also extracted reported quantitative data on intervention coverage and effectiveness (eg, sample size, point estimates, SD or errors, 95% CIs, p-values). The double-entry data were matched and any discrepancies were resolved through discussion, or by a third reviewer if needed.

We generated descriptive statistics to summarise key population and intervention characteristics, including population displacement status and intervention delivery characteristics, and we narratively synthesised factors inhibiting or facilitating the implementation of interventions by grouping reported information into common themes. We tabulated reported quantitative data on coverage and effectiveness; given the wide range of interventions, geographic settings, populations and delivery approaches across the publications, we did not undertake meta-analysis.

## Results

**Figure 1:**
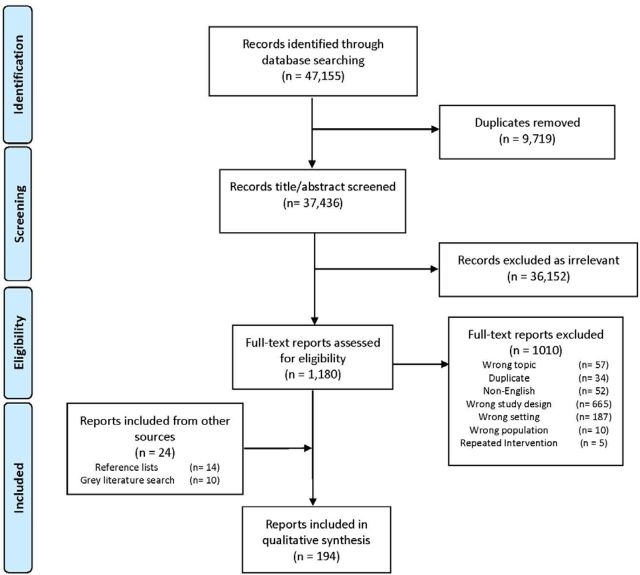
Preferred Reporting Items for Systematic Reviews and Meta-Analyses flow diagram: publication selection process for systematic review on the delivery of infectious disease interventions to women and children in conflict settings.

**Figure 2:**
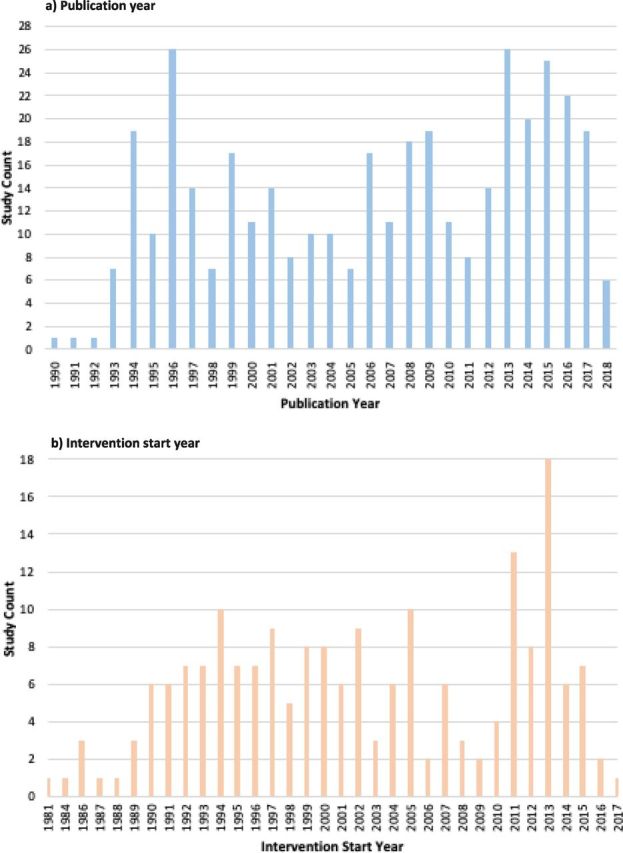
Publications counts by (a) publication year and (b) intervention start year.

A total of 37 436 unique citations were retrieved from indexed databases, with 170 ultimately assessed as eligible (figure 1). From the screening and assessment of the grey literature and of the reference lists of relevant systematic reviews, a further 24 eligible publications were identified. From the total of 194 eligible publications^18–211^ included in our review (table 1; online supplementary appendix B), we captured 392 reported instances of infectious disease intervention delivery. Publication frequency varied since the beginning of the review study period in 1990, with peaks in 1996 and again in 2013 (n=26), and remaining high since then (figure 2). The frequency of reported intervention start years also varied, with a peak in 1994 relating to the genocide in Rwanda and a similar peak in 2005 relating to civil war in DRC, and later peaks in 2011 and 2013 relating to the conflict in Syria.

**Table 1:** Summary of publication characteristics (n=194)*

*Geographic region*	*n*
Sub-Saharan Africa	106
East Asia and Pacific	41
South Asia	26
Middle East and North Africa	15
Europe and Central Asia	3
Latin America and the Caribbean	3
*Publication type*	*n*
Non-research report	44
Mixed methods	5
Observational	104
Non-randomised controlled trial	5
Randomised controlled trial	36
*Target population type†*	*n*
All/general population	115
All women	32
Pregnant women	16
Children<5 years only	27
Adolescents	0
*Displacement status of beneficiary population†*	*n*
IDPs	59
Refugees	102
Returning refugees	8
Non-displaced	45
Host	13
Unreported	16
*Setting of displaced populations‡*	*n*
Camp	101
Dispersed	26
Mixed	30
Unreported	3

*Refer to online supplementary appendix B for detailed information on included publications.

†Publications may be included in more than one category.

‡Only reflects publications that reported on displaced populations (refugees, IDPS, or returning refugees).

IDP, internally displaced person.

**Figure 3:**
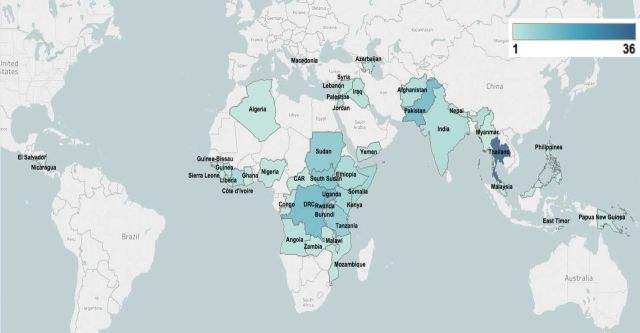
Geographic distribution of included publications.

**Figure 4:**
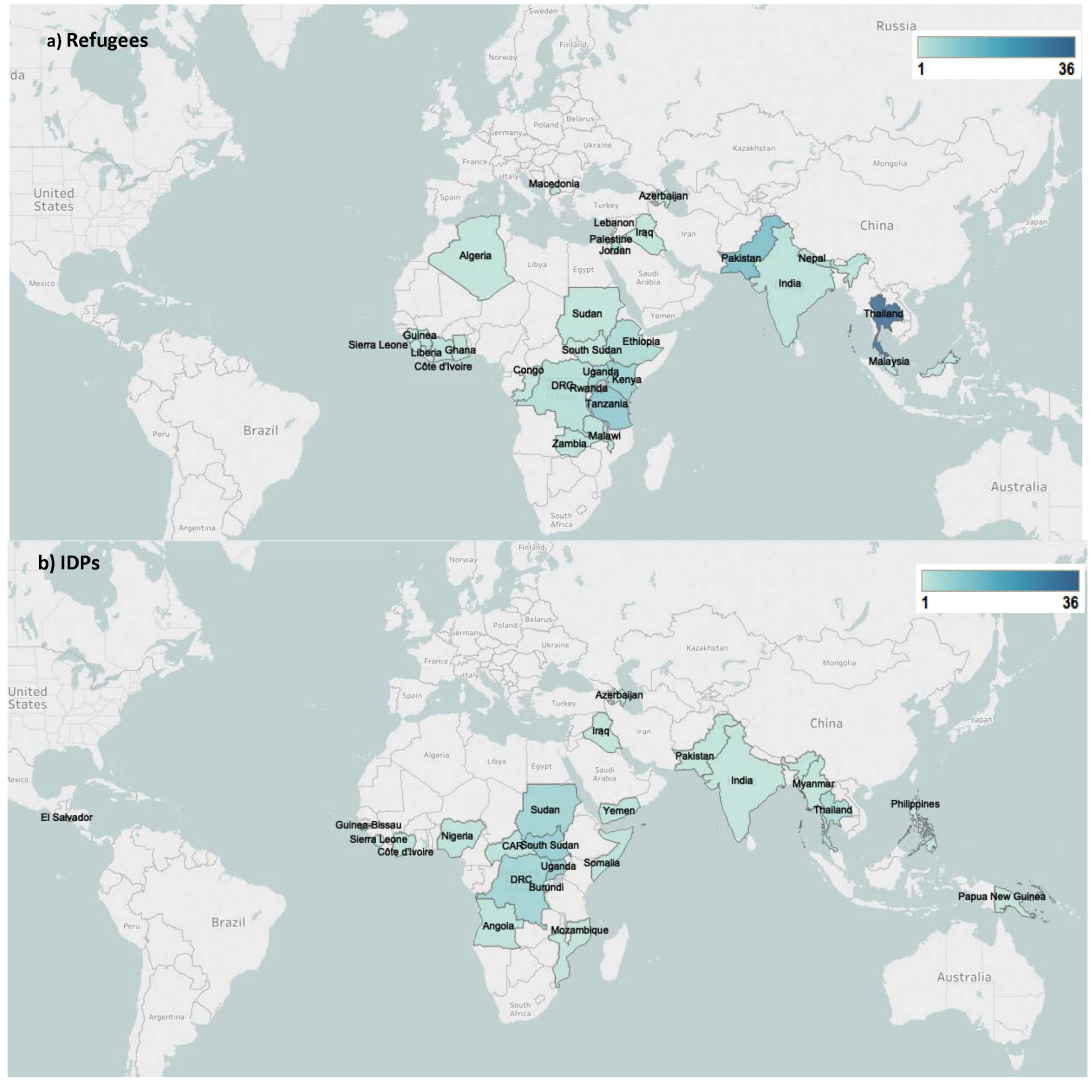
Geographic distribution of included publications by population displacement status. IDPs, internally displaced persons.

In regards to the geographic distribution of the included publications, more than half reported on intervention delivery in sub-Saharan Africa (106/194, 55%), most frequently in the DRC, South Sudan, Sudan, Tanzania and Ethiopia (figure 3). About 20% of included publications (41/194) were focused in East Asia, with Thailand accounting for the vast majority of these (36/41, 88%). South Asia (26 publications, 13%) and the Middle East and North Africa (15 publications, 8%) were the next most frequent regions, with the fewest publications focusing in Latin America (three publications, 2%), only consisting of El Salvador and Nicaragua, and Europe and Central Asia (three publications, 2%), including Macedonia and Azerbaijan. Of the included publications, 53% reported on intervention delivery in refugee populations and 30% in IDP populations. Publications from Uganda, South Sudan, DRC and Sudan predominantly focused on delivery to IDPs; publications from Thailand, Tanzania and Pakistan mostly focused on delivery to refugees from neighbouring countries (figure 4). Nearly 80% of publications reporting on intervention delivery to conflict-affected populations that were not displaced were focused in sub-Saharan Africa.

**Figure 5:**
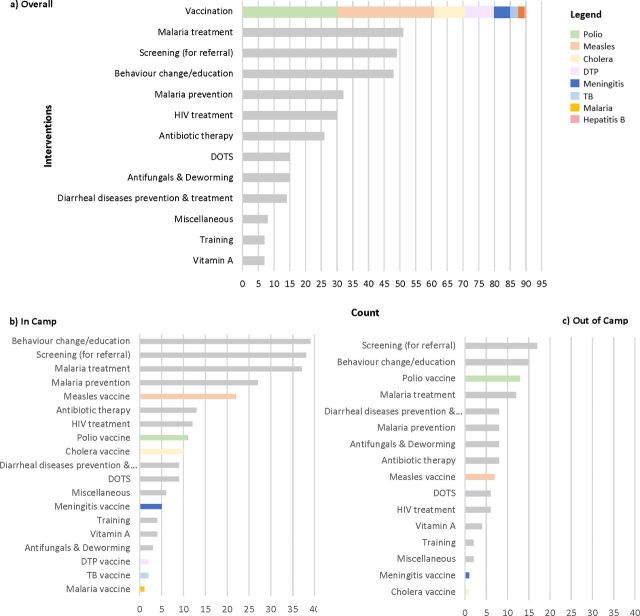
Frequencies of reported interventions delivered (a) overall, (b) in camps and (c) out of camps.

Overall, vaccines were the most commonly reported intervention delivered (figure 5). For interventions where there were mass immunisation campaigns, polio and measles vaccines were the most frequently reported vaccines administered to children under five. These high priority vaccines were often reported to be delivered in conjunction with one another. Vitamin A was also frequently reported to be given in conjunction with the measles vaccine. The delivery of cholera and diphtheria, tetanus and pertussis (DTP) vaccines were also frequently reported. After vaccines, malaria treatment was the second most commonly reported intervention, followed by screening for infectious diseases and behaviour change or education interventions. Screening for HIV or other sexually transmitted infections (STIs) was often reported to be integrated into antenatal care. Of the 49 reported instances of a screening intervention, 27 targeted HIV/STIs (56%), and 10 screened for malaria (21%); the remaining were for TB referrals. Behaviour change or education interventions accounted for 12% of reported interventions delivered. Examples of these interventions included condom provision, hygiene promotion and psychosocial support services. About 62% of the 392 reported instances of intervention delivery occurred in camp settings, and about 30% in non-camp environments, with the remaining 8% occurring among conflict-affected populations who were not displaced. Among both refugees and IDPs, a large majority of reported interventions were delivered within camps compared with out of camps, particularly vaccines (figure 5). The relative frequency of different intervention types was similar in both settings.

**Figure 6:**
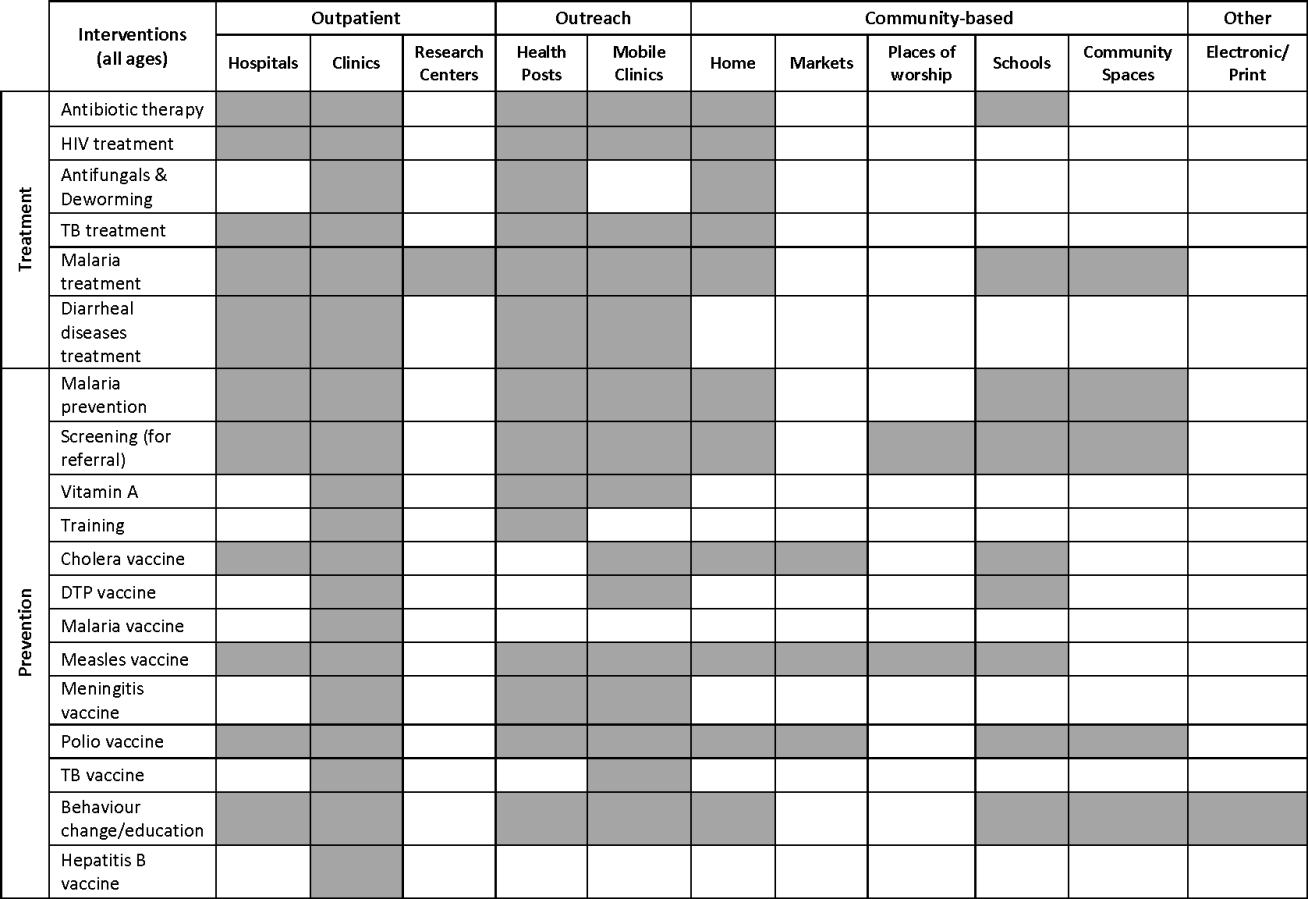
Reported intervention delivery sites. DTP, diphtheria, tetanus and pertussis; TB, tuberculosis.

**Figure 7:**
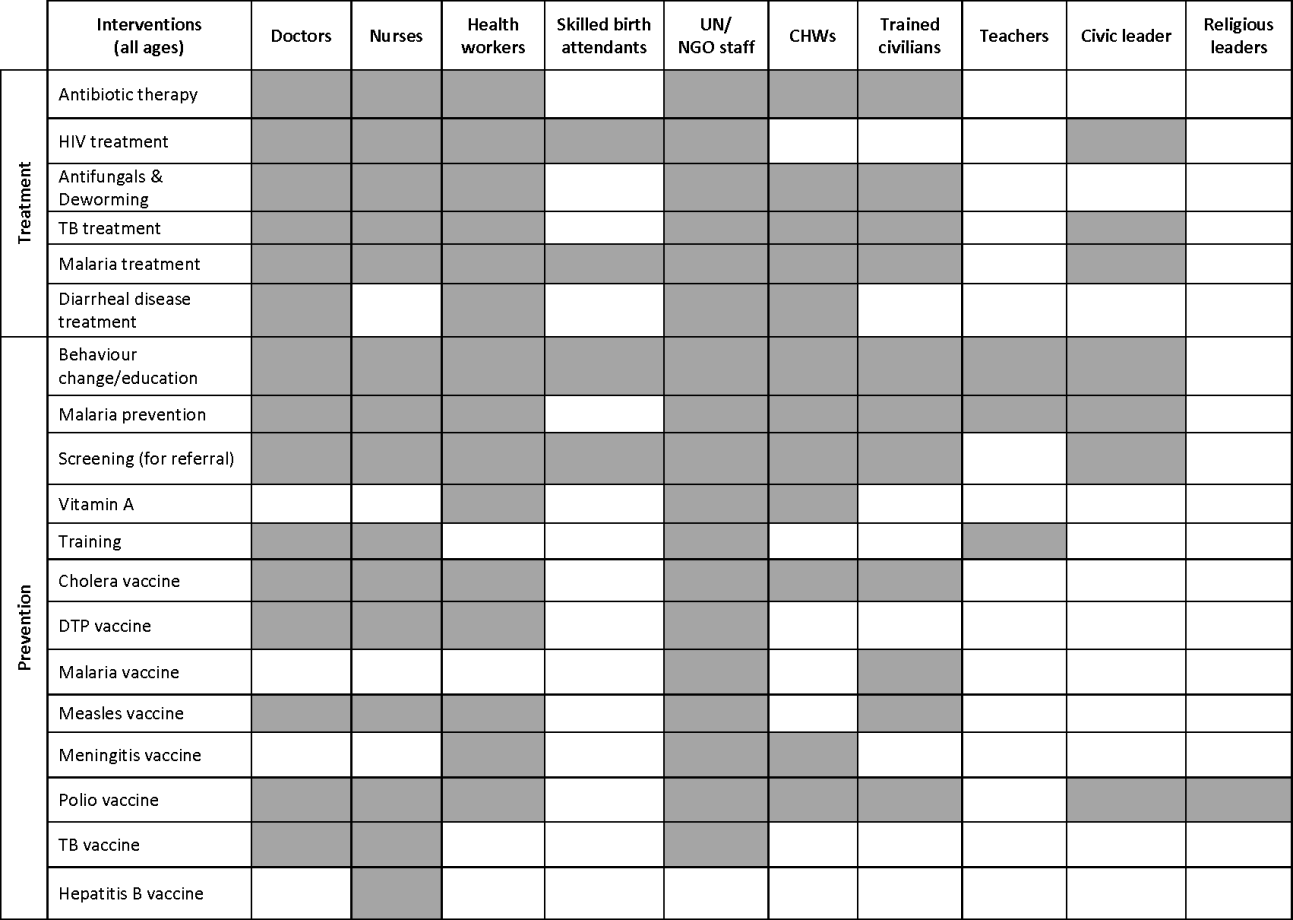
Reported intervention delivery personnel. DTP, diphtheria, tetanus and pertussis; TB, tuberculosis.

The delivery of interventions was categorised into three tiers of the care continuum: ‘outpatient’, consisting of delivery in hospitals and clinics; ‘outreach’, where service mobility is a component, such as delivery through temporary health posts or mobile clinics and ‘community-based’ care, which includes intervention delivery in homes, markets, places of worship, schools or other communal spaces (figure 6). Clinics were used as delivery sites for all reported intervention types, but outreach and community-based delivery were also common levels of care for intervention delivery. In terms of home-based delivery, malaria prevention interventions (eg, the distribution of insecticide-treated nets and indoor residual spraying) were the most frequently reported, while vaccines were also often delivered at the home using door-to-door strategies. Reported behaviour change/education interventions were mostly delivered in community settings, using the broadest range of delivery personnel types (figure 7). UN/NGO staff and health workers were the most frequently reported delivery personnel across all displacement settings, including among those who were non-displaced. Community health workers (CHWs) most often delivered health interventions to homes. Skilled birth attendants delivered interventions such as prevention of mother-to-child transmission of HIV and malaria treatment for pregnant women during antenatal care services. In some cases, civic and religious leaders were enlisted to encourage the uptake of polio vaccines where communities were misinformed or where public trust was compromised.^185 212^

Multiple barriers to implementing and scaling up the delivery of infectious disease interventions in conflict settings were highlighted in the included literature (table 2). Many of the obstacles revolved around limited access to target populations due to active combat, as well as logistical constraints, especially with the procurement and storage of vaccines. Public trepidation and reluctance to use health services delivered by foreign agencies also presented as a common barrier accounting for lower than expected coverage of some interventions. Other key barriers to delivery included a shortage of trained health workers, as well as stigma for receiving treatment for certain illnesses considered taboo, like HIV for example. Multiple strategies to counter some of these barriers were also documented in the literature. The most notable facilitator to implementing interventions was adopting social mobilisation strategies by coordinating a multisectorial approach. This was accomplished in out-of-camp settings by working with the Ministry of Health, engaging prominent local leaders, including CHWs in intervention delivery and integrating health promotion programmes at the community level to ensure accurate and harmonised messaging. Instituting reliable surveillance and population data in camp settings was also crucial for the roll-out of interventions. Some other common facilitators were capacity building—enriching the knowledge and practical skills of local workers, and ensuring that intervention delivery had an outreach component so it was flexible to move with populations.

**Table 2:** Barriers and facilitators to the delivery of infectious disease interventions in conflict settings

Barriers	
General themes	**Examples**
Constrained access	Difficulty accessing target populations due to ongoing conflict, insecurity and armed insurgency. This includes bans on health services and attacks on health workers by antigovernment groups or armed militia. A challenging physical environment, for example: rainy seasons, winter weather or long distances.
Community buy-in	Lack of community support and political hesitancy to embrace health campaigns from foreign agencies. Mistrust and misinformation from local community members, particularly around the administration of polio vaccines.
Poor infrastructure	Destruction of health infrastructure and a lack of transit centres along borders, causing limited access to facilities for care provision. Displaced persons who established living quarters in crowded areas with scarce sanitation and poor water supply, further facilitating an environment for the spread of highly infectious diseases like measles and poliovirus.
Logistics	Logistical problems with supply chain, specifically with the storage and shipment of vaccines to various vaccination sites. Resource constraints and rising costs due to the high demand of services, resulting in a shortage of diagnostic tools, drugs and equipment.
Human resources	Ongoing violence in conflict countries induced an exodus of thousands of doctors and nurses, seriously threatening the already strained health system. Major turnovers of international staff, a shortage of trained health workers and the presence of inexperienced teams during the early phases of an intervention contributed to delays in service delivery.
Stigma	Challenges noted with introducing HIV care into areas with minimal HIV knowledge; concerns that people diagnosed as HIV-positive could face serious negative consequences (eg, abandonment, physical violence, discrimination). Language barriers with care providers, and a fear of stigmatisation for receiving services for other taboo illnesses.
Mobile populations	High patient mobility was challenging for ensuring the continuity of care, particularly for HIV and TB treatment. Frequent movements of conflict-affected populations also accounted for lower than expected screening members and limited vaccination coverage during immunisation efforts.
Facilitators	
Social mobilisation	Forming strong partnerships with local community leaders (eg, elders, civic and religious figures) who leveraged their influence to negotiate access and promote community uptake of health interventions. Swift coordination with Ministry of Health or other partners in developing comprehensive operational plans to allocate resources quickly and efficiently. Employing strategies to provide knowledge to population; guided by promotion efforts (eg, radio broadcasting, precampaign focus-group interviews, etc)
Capacity building	Providing skills training to enrich and strengthen the role of CHWs and other national staff who are most familiar with the context; particularly useful for behavioural change/education and screening interventions. Optimising the use of pre-existing resources, facilities and tools with limited resources.
Safeguards and resource provision	Ensuring sufficient and working equipment for communication and feedback (eg, telephone/internet connection, camera, copier machine and computers). As well as obtaining sufficient resources and long-term commitment from aid agencies. Regular monitoring of the security situation and adapting contingency plans; allowing patients/staff coming from distant locations to stay near project sites.
Operational mobility	Flexibility to move ‘temporary fixed posts’ (ie, mobile clinics, health posts), frequently in response to caregivers’ demand to bring interventions (specifically vaccines) closer to their homes. Working within closed camps or areas with restricted movement of populations helped to ensure intervention coverage and the continuity of care.
Reliable surveillance	Instituting sustainable and reliable infectious disease surveillance helped to guide health planning for refugee populations. Detailed mapping of population settlements and their movements, aided in identifying communities with the largest target populations.
Negotiating ceasefires	Negotiating cease-fire or tranquillity days between warring factions, particularly for national immunisation days, allowing health workers to vaccinate children in areas with ongoing conflict.

CHWs, community health workers.

Only 15% of included publications (30/194) reported data on intervention coverage. Most reported on coverage data related to vaccinations, especially polio vaccines for children under 5 years and measles vaccine for those under 15 years, with some data available on cholera, DTP, tetanus, meningitis and TB vaccination coverage (online supplementary appendix C). Reported coverage rates for polio and measles vaccines often exceeded 80%. There was no observable difference between polio and measles coverage by displacement status or setting. Only 7% of publications (13/194) reported data on intervention effectiveness, most frequently evaluating malaria treatment regimens including different dosages of antimalarial medications, and malaria prevention interventions including insecticide-treated bednets and clothing.

## Discussion

### Principal findings

This systematic review identified 194 publications from 1990 to 2018 that report on delivery modalities of infectious disease interventions for women, children and adolescents in conflict-affected zones. While a range of delivered interventions was reported in the literature, interventions for vaccine-preventable diseases predominated, particularly for polio and measles virus. Malaria treatment in Thailand and infectious disease screening, especially for HIV across sub-Saharan Africa, were also reported frequently. Though clinic-based delivery was evident for all intervention types reported, many reported interventions were also delivered using outreach approaches, highlighting the advantage of mobile health services in many conflict settings. Due to the constraints of maintaining and accessing static clinics in areas of armed conflict, and because of high population movement, health personnel often provided interventions through the installation of health posts and mobile clinics. Moreover, most reported interventions were delivered in camps, likely reflecting the generally higher access to target populations in these settings, but perhaps also reflecting greater documentation of health service delivery in camp sites than in other settings. Few publications provided estimates for intervention coverage and effectiveness, with data for vaccine coverage being most commonly reported. Most of the reported intervention coverage and effectiveness data were captured from people living in camp settings. Multiple publications reported that collaboration through participatory processes involving prominent community actors, and modifying interventions to adapt to the cultural and religious context led to added coverage and impact. This includes inviting key stakeholders and respected community leaders to design programmes and assist with the dissemination and delivery of interventions.^213^

### Evidence gaps

The results of this review highlight several important gaps in the literature, relating to populations, geography and targeted health conditions. Very few publications were included from Latin America, with none from Colombia, despite having the most severe internal displacement situation in the world,^214^ or from other conflict-affected countries in the region such as Honduras, Nicaragua and El Salvador. Despite the need for adolescent-focused sexual and reproductive health programming including HIV/STI prevention and treatment in humanitarian settings,^213^ we found no reporting on infectious disease interventions targeting adolescents, and therefore no evidence on coverage rates in this population group. That much of the included literature focuses on the delivery of polio and measles vaccination is also not surprising. Poliovirus is of great concern in humanitarian crises as the move towards eradication is stalled when immunisation coverage is threatened by conflict; polio eradication relies on accessing over 95% of children with vaccines. Disturbances to routine immunisation and surveillance systems rapidly reduce population immunity, inevitably making individuals more susceptible to polio outbreaks. Measles is also of concern due to its highly contagious nature, especially among young children. Considering the mode of transmission, measles outbreaks are exacerbated by conflict and inhabiting overcrowded spaces.^215^ Nonetheless, the very limited reporting on the delivery of interventions for other important infectious disease conditions in the context of conflict is disproportional. Only one publication reported on the delivery of interventions for ARI including pneumonia, despite ARI morbidity often being very high in crowded camp settings.^216^ There was also limited reporting of neglected tropical disease (NTD) interventions, despite the elimination of NTDs by 2020 being a global goal. The major NTD focus in the literature was visceral/cutaneous leishmaniasis, likely due to the high morbidity and striking symptomology of the condition. Many NTDs often spike during warfare, with displacement in densely populated spaces, but very few publications reported on trachoma, deworming or other NTD interventions.^217^

### Limitations

Previous reviews of the literature have examined the size and quality of the evidence base for infectious disease interventions delivered in humanitarian settings,^11 12^ but this review is the first, to our knowledge, to focus on the mechanisms of intervention delivery to women and children affected by armed conflict. Nonetheless, this systematic review has several limitations. Methodologically, our decision to restrict the eligible literature to reports published only in English means that key information may have been missed, considering many conflicts occur in regions where English is not widely spoken and many humanitarian organisations operate and may document their operations in languages other than English. Our inclusion of interventions delivered within 5 years of the cessation of a conflict means that some interventions delivered during peacetime may also have been included inadvertently, potentially biassing our findings on how and by whom these interventions have been delivered in conflict settings and what the key delivery barriers and facilitators have been. The vast majority of the publications included in our review referred explicitly to delivery in a conflict context however, and so we would expect the influence of peacetime intervention delivery on our findings to be minimal. Furthermore, our approach in conducting a comprehensive but not exhaustive search of the grey literature means that we will have excluded other relevant publications, some of which may have provided different information on intervention delivery than what we have currently captured.

Beyond the limitations of our methodological approach, several limitations derive from the nature of the literature itself: much humanitarian health action goes undocumented and is not evaluated, and so it is not possible to estimate how well the infectious disease interventions and delivery patterns that have been reported and are captured here actually reflect those on the ground. Specifically, reporting on the coverage and effectiveness of these interventions was very poor. This major gap is likely due to multiple factors that make the monitoring and evaluation of intervention delivery difficult in conflict settings, including insecurity, high population mobility and a lack of both human and logistical resources to reliably and systematically collect and analyse quality data. Where coverage or effectiveness data were reported, very few publications reported gender-disaggregated data, precluding any analysis of potential gender differences. Further, where documentation of delivered interventions does exist (often in non-research reports or in cross-sectional studies), detailed information is rarely presented on how those interventions were delivered (eg, where and by whom, overcoming what barriers with what facilitators) or on their coverage or effectiveness. Finally, we must recognise that the highly contextual nature of humanitarian crises, with great variation geographically, socially, culturally and politically, can produce considerable heterogeneity in intervention delivery patterns and even intervention effectiveness across settings.

### Potential implications of findings for programming and future research

The limited information available on how such interventions are currently being delivered, and the paucity of data on achieved intervention coverage and effectiveness make recommendations on delivery strategies for different conflict contexts difficult to make. Our results strongly indicate that more rigorous and timely research is needed to identify effective strategies for delivering evidence-based infectious disease interventions in different conflict contexts. Future data collection should also enable gender analyses to be undertaken, to investigate gendered differences in health needs, access and uptake of services.

There is much variation in practice across UN/NGOs in programming for infectious disease interventions, suggesting a need for further consensus-building to develop a comprehensive set of standard operating procedures and implementation protocols for intervention delivery that could be accessed, adapted and applied in different conflict contexts. In the meantime, it is evident that infectious disease intervention planning must include components to addresses the security concerns of health service beneficiaries as well as providers, with a focus on restoring pre-existing facilities and operational functions that deliver essential services. At a broader level, preventing attacks on healthcare facilities and staff requires sustained effort at international, national and community levels. Moreover, garnering support and acceptance from local communities and influencers, including local authorities, appears to be critical, suggesting that health actors should aim to educate those around them while maintaining the perception of their impartiality and neutrality;^218 219^ further work to identify effective and efficient ways of doing this may be similarly critical. Further training and counselling of less-skilled health workers to enable appropriate task-shifting/sharing is required to better exploit delivery opportunities along the continuum of care, including community-based delivery.
