# Supplementary material for: Delivering infectious disease interventions to women and children in conflict settings: a systematic review
Source: BMJ Glob Health. 2020 Apr 26;5(Suppl 1):e001967. doi: 10.1136/bmjgh-2019-001967 (PMC7213813; doi:10.1136/bmjgh-2019-001967)
Supplement: Supplementary data [file bmjgh-2019-001967supp001.pdf]

Appendix A. Search stategy for Medline database

Conflict related terms:

disasters/ or emergencies/ or mass casualty incidents/

disaster victims/

((disaster or disasters or catastrophe or catastrophes) adj5 (environ* or human or manmade or "man made" or nature or natural or weather)).tw,kf.

("mass casualty" or "mass casualties" or "mass fatalities" or "mass fatality").tw,kf.

((crisis or crises) adj5 (environ* or human or manmade or "man made" or nature or natural or weather)).tw,kf.

"warfare and armed conflicts"/ or armed conflicts/ or warfare/ or biological warfare/ or bioterrorism/ or chemical warfare/ or chemical terrorism/ or nuclear warfare/ or psychological warfare/ or war crimes/ or ethnic cleansing/ or genocide/ or holocaust/ or war exposure/ or war-related injuries/

afghan campaign 2001-/ or gulf war/ or iraq war, 2003-2011/

("afghan campaign" or "armed conflict" or "armed conflicts" or "gulf war" or "iraq war" or "war time" or "wartime").tw,kf.

((armed or zone or political or civil) adj3 (conflict or conflicts or attack or attacks or war or wars or "no fly")).tw,kf.

("war related injuries" or "war related traumas" or "war related injury" or "war related trauma").tw,kf.

("militant group" or "militant groups" or "militant organization" or "militant organizations" or "militant organisation" or "militant organisations").tw,kf.

("biological terrorism" or bioterrorism or biowarfare or "chemical terrorism" or "ethnic cleansing" or "ethnic cleansings" or "gas poisoning" or genocide or holocaust or holocausts or "nuclear terrorism" or "war exposure" or "war exposures").tw,kf.

Disaster Medicine/

disease outbreaks/

Emergency Medical Services/

((emergency or emergencies) adj5 (environ* or human or manmade or "man made" or nature or natural or weather)).tw,kf.

Starvation/

(famine or famines or starvation or starvations).tw,kf.

cyclonic storms/ or droughts/ or floods/ or tornadoes/ or tidal waves/

avalanches/ or earthquakes/ or landslides/ or tidal waves/ or tsunamis/ or volcanic eruptions/

(avalanche or avalanches or cyclone or cyclones or drought or droughts or earthquake or earthquakes or flood or flooded or flooding or floods or hurricane or hurricanes or landslide or landslides or "land slide" or "land slides" or mudslide or mudslides or "mud slide" or "mud slides" or storm or storms or tornado or tornadoes or tsunami or tsunamis or typhoon or typhoons or "volcanic ash" or "volcanic eruption" or "volcanic eruptions" or "volcanic gases").tw,kf.

refugees/

(evacuee or evacuees or refugee or refugees or squatter or squatters or transients).tw,kf.

relief work/ or rescue work/

((rescue or relief or aid) adj (plan or plans or activity or activities or agency or agencies)).tw,kf.

("aid plan" or "aid work" or "relief plan" or "relief work" or "rescue plan" or "rescue work").tw,kf.

((staff or staffs or worker or workers) adj3 (relief or aid)).tw,kf.

(humanitarian assistance or humanitarian assistances or relief work or relief works).tw,kf.

(humanitarian adj2 (aid or response or relief or crisis or crises or emergency or emergencies or disaster or disasters)).tw,kf.

Altruism/

(humanitarianism or altruism).tw,kf.

("displaced children" or "displaced families" or "displaced family" or "displaced individuals" or "displaced internally" or "displaced men" or "displaced people" or "displaced peoples" or "displaced person" or "displaced persons" or "displaced population" or "displaced populations" or "displaced women" or "forced displacement" or "forced displacements" or "internal displaced" or "internal displacement" or "internally displaced" or "population displaced" or "population displacement").tw,kf.

(((camp or camps) and displac*) or "protected village*").tw,kf.

(victim or victims).tw,kf.

rubble.tw,kf.

or/1-35

<u>Population of interest:</u>

37. adolescent/ or young adult/

38. (adolescence or adolescent or adolescents or teen* or youth or youths or "young adult" or "young adults").tw,kf.

39. Pregnant Women/

40. exp pregnancy/

41. (expectant or expectancy or gravid* or pregnant or pregnancies or pregnancy).tw,kf.

42. ("mother to be" or "mothers to be").tw,kf.

43. (prenatal or "pre natal").mp.

44. (perinatal or "peri natal").mp.

45. ((trimester or trimesters) adj3 (first or second or mid or third or final or "1st" or "2nd" or "3rd")).tw,kf.

46. (midtrimester or midtrimesters or "early placental phase" or "early placental phases").tw,kf.

47. exp Delivery, Obstetric/

48. ((labor or labour) adj5 (birth* or breech or childbirth or childbirths or complicat* or difficult or early or easy or induce* or induction or late or obstetric* or onset or pregnan* or present*)).tw,kf.

49. parturients.tw,kf.

50. (birth or births or childbirth or childbirths or parturition or parturitions).tw,kf.

Meteke S, *et al. BMJ Global Health* 2020; 5:e001967. doi: 10.1136/bmjgh-2019-001967

51. ("abdominal deliveries" or "abdominal delivery" or "c-section" or "c-sections" or caesarean or caesareans or cesarean or cesareans or "postcesarean section" or "postcaesarean section").tw,kf.

52. exp Abortion, Induced/

53. (abortion or abortions or embryotomies or embryotomy or "postconception fertility control").tw,kf.

54. ((pregnancy or pregnancies) adj3 terminat*).tw,kf.

55. "sexually active".tw,kf.

56. child/ or child, preschool/ or infant/ or infant, newborn/ or infant, low birth weight/ or infant, small for gestational age/ or infant, very low birth weight/ or infant, extremely low birth weight/ or infant, postmature/ or infant, premature/ or infant, extremely premature/

57. (infan* or newborn* or "new born*" or neonat* or baby* or babies or toddler* or boy or boys or boyfriend or boyhood or girl* or kid or kids or child* or pediatric* or paediatric* or peadiatric* or prematur* or preterm*).mp. or school*.tw.

58. refugees/

59. (refugee or refugees).tw,kf.

60. or/37-59

61. 36 and 60

Domain specific terms – <u>Communicable diseases:</u>

62. exp bacteria/ or exp viruses/

63. (acquired immune deficiency syndrome or bacteria disease or bacteria diseases or bacteria infection or bacteria infections or bacterial disease or bacterial diseases or bacterial infection or bacterial infections or cholera or communicable disease or dengue or diarrheal disease or diarrheal diseases or diarrhoeal disease or diarrhoeal diseases or environmental microbiology or fungal infection or fungal infections or fungal disease or fungal diseases or helminthes or hepatitis or infectious disease or infectious diseases or leptospirosis or malaria or measles or meningitis or mould or mycoses or mycosis or parasite or parasites or respiratory infection or respiratory infections or scabies or sexually transmitted disease or sexually transmitted diseases or sexually transmitted infection or sexually transmitted infections or shigella or tuberculosis or typhoid or vaccine preventable disease or vaccine preventable diseases or viral disease or viral diseases or viral infection or viral infections or virus disease or virus diseases or virus infection or virus infections or worms or zoonoses or tetanus or "clostridium tetani infection").tw,kf.

64. 62 or 63

65. 61 and 64

66. limit 65 to ed=20170530-20180331

67. ("2017 05 30*" or "2017 05 31*" or "2017 06*" or "2017 07*" or "2017 08*" or "2017 09*" or "2017 10*" or "2017 11*" or "2017 12*" or "2018 01*" or "2018 02*" or "2018 03*").dt.

68. 65 and 67

69. 66 or 68.

~~~ End of Appendix ~~~
~~~

APPENDIX B. Characteristics of included publications

Author, Pub Year	Report Type	Country	Displacement Status	Displacement Setting	Target Population	Conditions Targeted	Interventions	Delivery Platform	Delivery Site	Delivery Personnel
**Cholera & Diarrhoeal Diseases**										
Apiyo 2014[12]	Non-Research	Somalia	IDPs, Hosts, Returning refugees	Dispersed	General population	Diarrhoeal diseases	Behaviour change/education	UN/NGO	Other-regional supply hubs	UN/NGO staff
Azman 2016[17]	Observational	South Sudan	IDPs	Camp	General population	Cholera	Vaccination, ORT	Healthcare, UN/NGO	Clinics, health posts	NR
Azman 2016[18]	Observational	South Sudan	IDPs, Not displaced	Camp, Dispersed	General population	Cholera	Vaccination	Healthcare, UN/NGO	NR	NR
Bekolo 2016[23]	Observational	South Sudan	IDPs, Not displaced	Camp	General population	Cholera	Vaccination	Healthcare, UN/NGO	Clinics, health posts	UN/NGO staff
Benny 2014[25]	Observational	Papua New Guinea	IDPs, Not displaced	Camp	General population	Shigellosis	Behaviour change/education, ORT	Healthcare	Communal spaces	Health workers, civic leaders
Bile 2011[26]	Non-Research	Pakistan	IDPs	Camp, Dispersed	General population	Diarrhoeal diseases, Malaria, Measles, TB	ORT, Malaria prevention, Malaria treatment, Vaccination, DOTS	NR	NR	NR
Boru 2013[28]	Observational	Kenya	Refugees	Dispersed	Children (<9y)	Diarrhoeal Diseases, Malaria, Worm Infestation	ORT, Malaria treatment, Deworming	Healthcare	Clinics	NR
Brown 1997[31]	Non-Research	Rwanda	Returning refugees	Dispersed	General population	Cholera, Diarrhoeal diseases	ORT, Diarrheal diseases prevention	Healthcare, UN/NGO	Clinics, health posts, mobile clinics	NR
Ciglenecki 2015[42]	Observational	South Sudan	IDPs	Camp	General population (>1y, excluding pregnant women)	Cholera, Measles, Meningitis, Polio	Vaccination	UN/NGO	NR	UN/NGO staff
Iyer 2016[86]	Observational	South Sudan	IDPs	Camp	General population (>1m)	Cholera	Vaccination	Healthcare, UN/NGO	Health posts, home	Doctors, nurses, UN/NGO staff
Lam 2017[103]	Observational	Iraq	IDPs, Refugees	Camp	General population (>1y)	Cholera	Vaccination	Healthcare, UN/NGO	Clinics, health posts, home, markets	Health workers
Legros 1999[104]	Observational	Uganda	Refugees	Camp	General population (>1y)	Cholera	Vaccination	Healthcare, UN/NGO	Health posts	CHWs, nurses, trained volunteers
Mahalanabis 2012[111]	Non-Research	India	Refugees	Camp, Dispersed	General population	Cholera	ORT, Antibiotics	Healthcare, UN/NGO	Hospitals	Health workers, UN/NGO staff, doctors
Marfin 1994[113]	Non-Research	Nepal	Refugees	Camp	General population	Cholera, Malaria, Measles, Pneumonia	ORT, Malaria prevention, Malaria treatment, Vaccination, Vitamin A, Antibiotics	Healthcare, UN/NGO	Clinics	CHWs, UN/NGO staff, health workers

Meteke S, *et al. BMJ Global Health* 2020; 5:e001967. doi: 10.1136/bmjgh-2019-001967

Matthys 1998[114]	Non-Research	DRC	Refugees	Camp	General population	Cholera	Behaviour change/education, ORT, Screening (for referral)	UN/NGO	Clinics	CHWs, health workers
Phares 2015[135]	Observational	Thailand	Refugees	Camp	General population (>1y, excluding pregnant women)	Cholera	Vaccination	Education, UN/NGO, Healthcare	Hospitals, health posts, home, schools	Health workers, UN/NGO staff
Porta 2014[138]	Observational	South Sudan	Host, Refugees	Camp	General population (>1y)	Cholera	Vaccination	Healthcare, UN/NGO	Health posts, home, markets	CHWs, UN/NGO staff
Salse 2013[171]	RCT	Uganda	Unreported	–	Children (<5y)	Diarrhoeal diseases, Malaria, Pneumonia	Antibiotics, Malaria treatment, ORT	Healthcare, UN/NGO	Hospitals	UN/NGO staff
Swerdlow 1997[181]	Observational	Malawi	Refugees	Camp	General population	Cholera	ORT	NR	Health posts	NR
Toole 1995[186]	Observational	DRC	Refugees	Camp, Dispersed	General population	Diarrhoeal diseases, Measles, Meningitis, Polio	Vaccination, Source-based water treatment (chlorination), Screening (for referral), Vitamin A	Defence, UN/NGO	NR	Health workers
WHO 2014[133]	Non-Research	South Sudan	IDPs	Camp	Females (>1y, excluding pregnant women)	Cholera	Vaccination	UN/NGO	Clinics, mobile clinics	UN/NGO staff, trained volunteers
**Pneumonia**										
Marfin 1994[113]	Non-Research	Nepal	Refugees	Camp	General population	Pneumonia, Cholera, Malaria, Measles	ORT, Malaria prevention, Malaria treatment, Vaccination, Vitamin A, Antibiotics	Healthcare, UN/NGO	Clinics	CHWs, UN/NGO staff, health workers
Salse 2013[171]	RCT	Uganda	Unreported	–	Children (<5y)	Pneumonia, Diarrhoeal diseases, Malaria	Antibiotics, Malaria treatment, ORT	Healthcare, UN/NGO	Hospitals	UN/NGO staff
**Vaccine-Preventable Diseases (VPDs)**										
Aaby 2003[1]	RCT	Guinea-Bissau	IDPs, Not displaced	Dispersed	Children (<1y)	Measles, Polio	Vaccination	Healthcare, UN/NGO, Research	Hospitals, clinics	UN/NGO staff, researchers
Aaby 2005[2]	Observational	Guinea-Bissau	IDPs, Not displaced	Dispersed	Children (<5y)	Polio	Vaccination	UN/NGO, Research	Health posts, home	UN/NGO staff, researchers
Adam 2015[4]	Observational	Sudan	IDPs	Camp	Females (15-49y)	Tetanus	Vaccination	NR	Clinics	NR
Ahoua 2006[8]	Observational	DRC	IDPs	Camp	Children, Adolescents (<14y)	Measles	Vaccination	UN/NGO	NR	NR
Alleman 2018[10]	Non-Research	DRC	Not displaced	–	Children (<5y)	Polio	Vaccination	UN/NGO	NR	NR

Meteke S, *et al. BMJ Global Health* 2020; 5:e001967. doi: 10.1136/bmjgh-2019-001967

Bile 2011[26]	Non-Research	Pakistan	IDPs	Camp, Dispersed	General population	Measles, TB, Diarrhoeal diseases, Malaria	ORT, Malaria prevention, Malaria treatment, Vaccination, DOTS	NR	NR	NR
CDC 1999[36]	Non-Research	Afghanistan	Unreported	–	Children (<5y)	DTP, Measles, Polio	Vaccination	UN/NGO	NR	Health workers
CDC 1999[37]	Non-Research	Somalia, Sudan	Not displaced	–	Children (<5y)	Polio	Vaccination	UN/NGO	NR	Trained volunteers
CDC 1999[38]	Non-Research	Angola	IDPs, Not displaced	Dispersed	Children (<5y)	Polio	Vaccination	Healthcare, UN/NGO	NR	NR
Cetorelli 2013[39]	Observational	Iraq	Unreported	–	Neonates (<28d)	Polio	Vaccination	Healthcare, UN/NGO	NR	NR
Ciglenecki 2015[42]	Observational	South Sudan	IDPs	Camp	General population (>1y, excluding pregnant women)	Measles, Meningitis, Polio, Cholera	Vaccination	UN/NGO	NR	UN/NGO staff
de Lima Pereira 2018[48]	Observational	Syria	Unreported	–	Children (<5y)	Measles	Vaccination	Healthcare, UN/NGO	Clinics, schools	Health workers
Duroch 2014[54]	Non-Research	DRC	IDPs	NR	General population	Tetuanus, Hepatitis B, HIV, STIs	Antibiotics, ART, Vaccination	UN/NGO	Clinics	UN/NGO staff
Elsayed 2004[57]	Non-Research	Sudan	IDPs	Camp, Dispersed	Children, Adolescents (<19y)	Measles, Polio	Vaccination, Vitamin A	Healthcare, UN/NGO	Clinics, health posts, mobile clinics	Health workers
Feroz 2003[63]	Non-Research	Afghanistan	Returning refugees, Not displaced	Dispersed	Children, Adolescents (<14y)	Measles	Vaccination	Faith-based, Healthcare, UN/NGO	Health posts, mobile clinics, markets, places of worship	UN/NGO staff, trained volunteers
Garenne 1997[65]	Observational	Mozambique	Not displaced	–	Children (<9y)	DTP, Measles, Polio, TB	Vaccination, Vitamin A	UN/NGO	NR	UN/NGO staff
Gaspar 2000[66]	Observational	Angola	IDPs, Not displaced	Dispersed	Children, Adolescents (<14y)	Polio	Vaccination	Healthcare	NR	NR
Goodson 2012[70]	Non-Research	Ethiopia, Kenya, Somalia	Refugees	Camp	Females (<34y)	Measles	Vaccination	UN/NGO	Hospitals, home	NR
Habib 2017[75]	RCT	Pakistan	Unreported	–	General population, Children (<5y)	Polio	Behaviour change/education, Vaccination	UN/NGO	Home	CHWs, health workers
Haelterman 1996[76]	Observational	DRC	Refugees	Camp	General population (>1m)	Meningitis	Vaccination	UN/NGO	Health posts	UN/NGO staff, health workers
Hindiyeh 2009[82]	Observational	Palestine	Refugees	Camp	Females (<34y)	Measles	Vaccination	UN/NGO	NR	NR
Kamadjeu 2014[91]	Observational	Somalia	Not displaced	–	General population	Polio	Vaccination	Healthcare, UN/NGO	NR	NR
Kline 1999[97]	Non-Research	Macedonia	Refugees	Camp	Children (<1y)	DTP, Measles, Polio, TB	Vaccination	Healthcare, UN/NGO	Clinics	Doctors, nurses, administrator s
Koop 2001[98]	Non-Research	Macedonia	Refugees	Camp	Children (<5y)	DTP, Measles, Polio, TB	Vaccination	Healthcare, UN/NGO	Clinics, mobile clinics	UN/NGO staff, doctors

Meteke S, *et al. BMJ Global Health* 2020; 5:e001967. doi: 10.1136/bmjgh-2019-001967

Koscalova 2014[99]	Non-Research	DRC	Unreported	–	General population	Measles, Malaria	Malaria treatment, Screening (for referral), Vaccination	UN/NGO	Hospitals, clinics, health posts, mobile clinics	Doctors, nurses, UN/NGO staff
Kouadio 2009[100]	Observational	Côte d'Ivoire	Refugees	Camp	Children, Adolescents (<19y)	Measles	Behaviour change/education, Vaccination	UN/NGO	Health posts	UN/NGO staff, doctors, nurses, health workers
Makokha 2014[112]	Observational	Kenya	Host, Refugees	Camp, Dispersed	Children (<5y)	Polio	Vaccination	Healthcare, UN/NGO	Clinics, health posts	CHWs, health workers
Marfin 1994[113]	Non-Research	Nepal	Refugees	Camp	General population	Measles, Cholera, Malaria, Pneumonia	ORT, Malaria prevention, Malaria treatment, Vaccination, Vitamin A, Antibiotics	Healthcare, UN/NGO	Clinics	CHWs, UN/NGO staff, health workers
Mbaeyi 2017[116]	Non-Research	Iraq, Syria	Unreported	–	Children (<5y)	Polio	Vaccination	Healthcare	Clinics, mobile clinics, home, communal spaces	NR
Mupere 2005[124]	Observational	Uganda	IDPs	Camp	General population	Measles	Vaccination	Healthcare	NR	NR
Navarro-Colorado 2014[125]	Observational	Ethiopia, Kenya	Refugees	Camp	General population	Measles	Vaccination	Healthcare, UN/NGO	Hospitals, clinics, health posts	NR
Nnadi 2017[126]	Non-Research	Nigeria	IDPs, Not displaced	Dispersed	Children (<5y)	Polio	Vaccination	Healthcare	Home	Health workers
Petersen 2013[134]	Non-Research	Jordan	Refugees	Camp, Dispersed	Females (<34y)	Measles, Polio	Vaccination	Healthcare, UN/NGO	Clinics, mobile clinics	NR
Porter 1990[139]	Observational	Malawi	Refugees	Camp	Children (<5y)	Measles	Vaccination	Healthcare, UN/NGO	Clinics	Health workers, UN/NGO staff
Richardson 2013[152]	Non-Research	Ethiopia	Refugees	Camp	Children, Adolescents (<19y)	Measles	Vaccination	UN/NGO	Transit centres	UN/NGO staff
Rull 2018[166]	Non-Research	South Sudan	IDPs	Camp	General population	Measles, Meningitis, Polio	Vaccination	UN/NGO	Clinics	NR
Santaniello-Newton 2000[172]	Observational	Uganda	Refugees	Camp	Females (<34y)	Meningitis	Vaccination	UN/NGO	Clinics, health posts	CHWs
Shuaibu 2016[176]	Observational	Nigeria	IDPs, Not displaced	Camp, Dispersed	Children (<5y)	Polio	Vaccination	Healthcare, UN/NGO	Clinics, mobile clinics, home, markets, schools	Civic leaders, doctors, nurses, religious leaders, UN/NGO staff, health workers

Meteke S, *et al. BMJ Global Health* 2020; 5:e001967. doi: 10.1136/bmjgh-2019-001967

Toole 1995[186]	Observational	DRC	Refugees	Camp, Dispersed	General population	Measles, Meningitis, Polio, Diarrhoeal diseases	Vaccination, Source-based water treatment (chlorination), Screening (for referral), Vitamin A	Defence, UN/NGO	NR	Health workers
UNICEF 2014[59]	Non-Research	India	Unreported	–	Children (<5y)	Polio	Vaccination	UN/NGO	Home	UN/NGO staff
UNICEF 2016[184]	Non-Research	CAR	IDPs	Camp, Dispersed	Children (<5y), Pregnant women	Measles, HIV, Malaria	ART, Malaria prevention, Vaccination, Vitamin A	UN/NGO	NR	UN/NGO staff
Vitek 2000[192]	Observational	Azerbaijan	Unreported	–	Children, Adolescents (<19y)	DTP	Vaccination	Healthcare, UN/NGO	Schools	NR
WHO 1994[60]	Non-Research	Somalia, Sudan	IDPs, Refugees	Dispersed	Children (<9y)	Polio	Vaccination	Healthcare	NR	NR
WHO 2000[146]	Non-Research	DRC	Not displaced	–	Children (<5y)	Measles, Polio	Vaccination	Healthcare, UN/NGO	NR	Health workers
WHO 2004[140]	Observational	Sudan	IDPs, Not displaced	Camp	Children, Adolescents (<19y)	Measles	Vaccination	Healthcare, UN/NGO	Clinics, health posts, mobile clinics	UN/NGO staff, trained volunteers
WHO 2013[137]	Non-Research	Kenya, Somalia	Host, Refugees	Camp, Dispersed	Children, Adolescents (<14y)	Polio	Vaccination	Healthcare, UN/NGO	Health posts, mobile clinics	UN/NGO staff
WHO 2014[44]	Observational	Kenya	Host, Refugees	Dispersed	Children (<5y)	Polio	Vaccination	UN/NGO	Clinics, health posts, mobile clinics, home	Health workers, trained volunteers
**Malaria**										
Ambler 2009[11]	RCT	Thailand	Refugees	Camp	Children (<5y)	Malaria	Malaria treatment	Research	Clinics	NR
Ashley 2004[13]	RCT	Thailand	Refugees, Not displaced	Camp	General population (excluding pregnant and postnatal mothers)	Malaria	Malaria treatment	Research	Hospitals, clinics	Nurses, UN/NGO staff
Ashley 2005[14]	RCT	Thailand	Refugees, Not displaced	Camp	General population (excluding pregnant and postnatal mothers)	Malaria	Malaria treatment	Research	Clinics	Researchers
Ashley 2006[15]	RCT	Thailand	Refugees, Not displaced	Camp	General population (excluding pregnant and postnatal mothers)	Malaria	Malaria treatment	Research	Clinics	Researchers
Bile 2011[26]	Non-Research	Pakistan	IDPs	Camp, Dispersed	General population	Malaria, Measles, TB, Diarrhoeal diseases	ORT, Malaria prevention, Malaria treatment, Vaccination, DOTS	NR	NR	NR

Meteke S, *et al. BMJ Global Health* 2020; 5:e001967. doi: 10.1136/bmjgh-2019-001967

Boru 2013[28]	Observational	Kenya	Refugees	Dispersed	Children (<9y)	Malaria, Worm Infestation, Diarrhoeal Diseases	ORT, Malaria treatment, Deworming	Healthcare	Clinics	NR
Bouma 1996[29]	Non-randomized CT	Pakistan	Refugees, Not displaced	Camp, Dispersed	General population	Malaria	Malaria prevention	UN/NGO	Home	Health workers
Brooks 2017[30]	Mixed methods	DRC	IDPs, Not displaced	Camp	General population	Malaria	Malaria prevention, Malaria treatment, Screening (for referral)	Healthcare, UN/NGO	Clinics, home	CHWs
Burns 2012[32]	RCT	Sierra Leone	Refugees	Camp	General population	Malaria	Malaria prevention, Malaria treatment, Screening (for referral)	UN/NGO	Clinics, Home	UN/NGO staff, health workers, nurses
Carrara 2006[34]	Observational	Thailand	IDPs, Host, Refugees, Not displaced	Camp, Dispersed	General population	Malaria	Malaria prevention, Malaria treatment, Screening (for referral)	Healthcare, Research	Clinics, health posts, mobile clinics, home	Health workers
Charchuk 2015[40]	Observational	South Sudan	Not displaced	–	Children, Adolescents (<19y)	Malaria	Malaria treatment, Screening (for referral)	UN/NGO	Schools	Nurses
Charlwood 200[141]	RCT	Sudan	Refugees	Camp	General population	Malaria	Malaria prevention	UN/NGO	Home	NR
Coldiron 2017[43]	Observational	Uganda	Host, Refugees	Camp	Children, Adolescents (<14y)	Malaria	Malaria prevention	UN/NGO	Clinics, home	Health workers, other - children's caretakers
Depoortere 2004[49]	Observational	Zambia	Refugees	Camp	Children (<9y)	Malaria	Malaria treatment	UN/NGO	Clinics, home	Health workers
Depoortere 2005[50]	RCT	Zambia	Refugees	Camp	Children (<5y)	Malaria	Malaria treatment	UN/NGO	Clinics	NR
Dolan 1993[52]	RCT	Thailand	Refugees	Camp	Pregnant women	Malaria	Malaria prevention	UN/NGO	Hospitals	Health workers
Ezard 2003[61]	Observational	East Timor	Unreported	–	Females (<34y)	Malaria	Malaria treatment	Healthcare, UN/NGO	Hospitals	NR
Ghebreyesus 1996[68]	Observational	Ethiopia	Not displaced	–	General population	Malaria	Behaviour change/education, Malaria prevention, Malaria treatment, Screening (for referral), Training	Healthcare, UN/NGO	Communal spaces	CHWs, UN/NGO staff, health workers
Graham 2002[73]	RCT	Pakistan	Refugees	Camp	General population	Malaria	Malaria prevention	UN/NGO	Home	NR
Graham 2002[74]	Non-randomized CT	Pakistan	Refugees	Camp	General population	Malaria	Malaria prevention	UN/NGO	Home	NR
Hamze 2016[78]	Observational	DRC	IDPs	Camp	General population	Malaria	Malaria treatment, Screening (for referral)	NR	Clinics, home	CHWs, UN/NGO staff
Howard 2011[83]	Non-randomized CT	Pakistan	Refugees	Camp	General population	Malaria	Malaria treatment	UN/NGO, Research	NR	Health workers

Meteke S, *et al. BMJ Global Health* 2020; 5:e001967. doi: 10.1136/bmjgh-2019-001967

Hutagalung 2005[84]	RCT	Thailand	Refugees, Not displaced	Camp	General population (excluding pregnant women)	Malaria	Malaria treatment	Research	Clinics	Researchers
ICRC 2016[85]	Non-Research	Philippines	IDPs	NR	General population	Malaria, Dengue	Malaria prevention	UN/NGO	NR	UN/NGO staff
Kajeechiwa 2016[90]	Observational	Thailand	Refugees	Dispersed	General population (>1m)	Malaria	Malaria treatment	Healthcare, Research	Clinics	Health workers
Kasereka 2014[92]	Observational	DRC	IDPs	Camp	Children (<5y)	Malaria	Malaria treatment, Screening (for referral)	NR	Clinics, home	CHWs
Kimani 2006[96]	RCT	Kenya	Refugees	Camp	General population	Malaria	Malaria prevention	Informal governance, Healthcare, UN/NGO	Home	CHWs, UN/NGO staff, trained volunteers, nurses
Koscalova 2014[99]	Non-Research	DRC	Unreported	–	General population	Malaria, Measles	Malaria treatment, Screening (for referral), Vaccination	UN/NGO	Hospitals, clinics, health posts, mobile clinics	Doctors, nurses, UN/NGO staff
Luxemburger 1994[106]	RCT	Thailand	Refugees	Camp	Children, Adolescents (<19y)	Malaria	Malaria prevention	UN/NGO	Clinics, home	UN/NGO staff, health workers
Luxemburger 1994[107]	RCT	Thailand	Refugees	Camp	General population (>1m, excluding pregnant women)	Malaria	Malaria treatment	UN/NGO, Research	Clinics	NR
Luxemburger 1995[108]	RCT	Thailand	Refugees	Camp	General population (>1y, excluding pregnant women)	Malaria	Malaria prevention, Malaria treatment	UN/NGO	Hospitals	Doctors, nurses, UN/NGO staff
Luxemburger 1996[109]	Observational	Thailand	Refugees	Camp	General population	Malaria	Malaria treatment	UN/NGO	Home, schools	Health workers
Luxemburger 1999[110]	Observational	Thailand	Refugees	Camp	General population (>1y, excluding pregnant and post-natal mothers)	Malaria	Malaria treatment	UN/NGO, Research	Research centres	NR
Marfin 1994[113]	Non-Research	Nepal	Refugees	Camp	General population	Malaria, Measles, Pneumonia, Cholera	ORT, Malaria prevention, Malaria treatment, Vaccination, Vitamin A, Antibiotics	Healthcare, UN/NGO	Clinics	CHWs, UN/NGO staff, health workers
McGready 2012[119]	Observational	Thailand	Refugees	Camp	Pregnant women	Malaria	Malaria treatment	UN/NGO, Research	Clinics	Doctors, nurses, health workers, midwives
Nosten 1991[127]	Observational	Thailand	Refugees	Camp	Pregnant women	Malaria	Malaria treatment	UN/NGO	Clinics	Health workers
Nosten 1999[128]	RCT	Thailand	Refugees	Camp	Children, Adolescents (<19y)	Malaria	Malaria treatment	UN/NGO, Research	Clinics	UN/NGO staff, trained volunteers

Meteke S, *et al. BMJ Global Health* 2020; 5:e001967. doi: 10.1136/bmjgh-2019-001967

Nosten 1999[129]	Observational	Thailand	Refugees	Camp	Pregnant women	Malaria	Vaccination	UN/NGO	Clinics	NR
Obol 2013[130]	Observational	Uganda	IDPs	Camp	Pregnant women	Malaria	Malaria prevention	UN/NGO	Clinics	UN/NGO staff
Price 1995[141]	RCT	Thailand	Refugees	Dispersed	General population (>1m, excluding pregnant women)	Malaria	Malaria treatment	UN/NGO, Research	Clinics	NR
Price 1996[142]	RCT	Thailand	Refugees	Dispersed	General population (>1m, excluding pregnant women)	Malaria	Malaria treatment	NR	Clinics	NR
Price 1997[143]	Observational	Thailand	Refugees	Dispersed	General population (>1m, excluding pregnant women)	Malaria	Malaria treatment	UN/NGO, Research	Clinics	NR
Price 1998[144]	RCT	Thailand	Refugees	Dispersed	General population (>1m, excluding pregnant women)	Malaria	Malaria treatment	UN/NGO, Research	Hospitals	NR
Price 1998[145]	RCT	Thailand	Refugees	Camp	General population (>1m, excluding pregnant women)	Malaria	Malaria treatment	UN/NGO, Research	Clinics, home	Home visitor
Protopopoff 2007[147]	Observational	Burundi	IDPs	Camp, Dispersed	General population	Malaria	Malaria prevention, Malaria treatment	Healthcare, UN/NGO	Clinics, home, schools	Trained volunteers
Richards 2009[151]	Observational	Myanmar	IDPs	Dispersed	General population	Malaria	Behaviour change/education, Malaria prevention, Malaria treatment, Screening (for referral)	Healthcare, UN/NGO	Clinics, communal spaces, home	Civic leaders, CHWs, trained volunteers
Roca 2011[153]	Observational	Tanzania	Host, Refugees	Camp	General population	Malaria	Malaria treatment	Healthcare, UN/NGO	Clinics	Health workers
Rowland 1994[156]	Non-randomized CT	Pakistan	Refugees	Camp	General population	Malaria	Malaria prevention	Education, UN/NGO	Home, Schools	NR
Rowland 1996[157]	RCT	Pakistan	Refugees	Camp	General population	Malaria	Malaria prevention	Healthcare, UN/NGO	Home	CHWs
Rowland 1997[158]	Observational	Pakistan	Refugees	Camp	General population	Malaria	Malaria treatment	UN/NGO	Clinics, home	UN/NGO staff, health workers
Rowland 1999[159]	RCT	Pakistan	Refugees	Camp	General population	Malaria	Malaria prevention	UN/NGO	Home	CHWs, UN/NGO staff
Rowland 1999[160]	RCT	Pakistan	Refugees	Camp	General population (>1y, excluding pregnant women)	Malaria	Malaria prevention	Healthcare, UN/NGO	Clinics, home	UN/NGO staff, outreach workers
Rowland 2001[161]	Observational	Pakistan	Refugees	Camp	General population	Malaria	Malaria prevention, Malaria treatment	UN/NGO	Home	Trained volunteers

Meteke S, *et al. BMJ Global Health* 2020; 5:e001967. doi: 10.1136/bmjgh-2019-001967

Rowland 2002[162]	RCT	Afghanistan	Returning refugees	Camp	General population	Malaria	Behaviour change/education, Malaria prevention	UN/NGO	Mobile clinics	NR
Rowland 2004[163]	RCT	Pakistan	Refugees	Camp	General population	Malaria	Behaviour change/education, Malaria prevention	UN/NGO	Home	CHWs
Ruckstuhl 2017[165]	Observational	CAR	IDPs, Not displaced	Dispersed	General population	Malaria	Malaria prevention, Malaria treatment, Deworming, ORT, Vitamin A, Training	UN/NGO	Clinics	CHWs, doctors, nurses
Salse 2013[171]	RCT	Uganda	Unreported	–	Children (<5y)	Malaria, Pneumonia, Diarrhoeal diseases	Antibiotics, Malaria treatment, ORT	Healthcare, UN/NGO	Hospitals	UN/NGO staff
Smithuis 1993[177]	RCT	Thailand	Refugees	Camp	General population	Malaria	Malaria treatment	UN/NGO	Hospitals, clinics	NR
Spencer 2004[179]	Observational	Uganda	IDPs	Camp	General population	Malaria	Behaviour change/education, Malaria prevention	Healthcare, UN/NGO	Schools	UN/NGO staff, educators
Ter Kuile 1993[183]	RCT	Thailand	Refugees	Camp	General population	Malaria	Malaria treatment	UN/NGO	Clinics	NR
Tomashek 2001[185]	RCT	Tanzania	Refugees	Camp	Children (<5y)	Malaria	Malaria treatment	UN/NGO	Clinics, home	Home health visitor, UN/NGO staff
van Vugt 1998[188]	RCT	Thailand	IDPs	Camp	Females (5-65y)	Malaria	Malaria treatment	UN/NGO, Research	Clinics, home	NR
van Vugt 1999[189]	RCT	Thailand	IDPs	camp	Females (>1y)	Malaria	Malaria treatment	UN/NGO, Research	Clinics, home	NR
van Vugt 2000[190]	RCT	Thailand	IDPs	Camp	Females (>1y)	Malaria	Malaria treatment	UN/NGO, Research	Hospitals, clinics	NR
van Vugt 2002[191]	RCT	Thailand	Refugees	Camp	General population (>1y, excluding pregnant women)	Malaria	Malaria treatment	UN/NGO, Research	Clinics	UN/NGO staff, researchers
McGready 2000[118]	RCT	Thailand	Refugees	Camp	Pregnant women	Malaria, HIV	ART, Malaria treatment	UN/NGO, Research	Clinics	Midwives
UNICEF 2016[184]	Non-Research	CAR	IDPs	Camp, Dispersed	Children (<5y), Pregnant women	Malaria, Measles, HIV	ART, Malaria prevention, Vaccination, Vitamin A	UN/NGO	NR	UN/NGO staff
**HIV & STIs**										
Adam 2016[4]	Observational	Sudan	IDPs	Camp	Females (>15y)	HIV	Behaviour change/education	UN/NGO	Clinics, health posts	Doctors, nurses, birth attendants, health workers
Augusto 2016[16]	Observational	Angola	Not displaced	–	Pregnant women	HIV	ART, Screening (for referral)	Healthcare	Clinics	NR

Banks 2016[20]	Observational	Thailand	Refugees	Camp	Pregnant women	HIV, Hepatitis B	Screening (for referral)	Healthcare, Research	Clinics	NR
Bannink-Mbazzi 2013[21]	Observational	Uganda	IDPs, Not displaced	Camp	Children (<5y), Pregnant women	HIV	ART, Behaviour change/education, Screening (for referral)	Healthcare, UN/NGO	Clinics, electronic/print	NR
Benjamin 1996[24]	Non-Research	Tanzania	Refugees	Camp	General population	HIV, STIs	ART, Behaviour change/education, Screening (for referral)	Healthcare, UN/NGO	Clinics, home, electronic/print, communal spaces	Counselors, UN/NGO staff, trained volunteers
Casey 2006[35]	Observational	Sierra Leone	IDPs	Camp, Dispersed	Females (15-34y)	HIV, STIs	Behaviour change/education	UN/NGO	NR	Health workers
Cossa 1994[46]	Observational	Mozambique	IDPs	Camp	Pregnant women	HIV, Syphilis	Antibiotics, Behaviour change/education, Screening (for referral)	Healthcare	Health posts	Doctors, nurses
Culbert 2007[47]	Observational	DRC	Unreported	–	General population	HIV	ART, Behaviour change/education, Screening (for referral)	Healthcare, UN/NGO	Clinics	Doctors, nurses, trained volunteers health, workers
Doumbouya 2012[53]	Observational	Côte d'Ivoire	Not displaced	–	General population	HIV	ART	Healthcare, UN/NGO	Clinics	Counsellors, health workers
Duroch 2014[54]	Non-Research	DRC	IDPs	NR	General population	HIV, STIs, Hepatitis B, Tetuanus	Antibiotics, ART, Vaccination	UN/NGO	Clinics	UN/NGO staff
Ehui 2015[55]	Observational	Côte d'Ivoire	Unreported	–	Children, Adolescents (<19y)	HIV	ART, Antibiotics	Research	Hospitals, clinics	Health workers, social workers
Ellman 2005[56]	Non-Research	DRC	Not displaced	–	General population	HIV	ART	UN/NGO	Clinics	Health workers
Erickson 2015[58]	Observational	Uganda	IDPs, Refugees	Camp	General population (>10y)	HIV	Screening (for referral)	UN/NGO, Research	Clinics	NR
Fabiani 2006[62]	Observational	Uganda	IDPs	Camp, Dispersed	Pregnant women	HIV	Counselling, Screening (for referral)	Healthcare, Research	Hospitals, clinics	NR
Garang 2009[64]	Observational	Uganda	IDPs, Not displaced	Camp, Dispersed	General population (>15y)	HIV	ART	UN/NGO	Hospitals	Doctors, UN/NGO staff, health workers
Goodrich 2013[69]	Non-Research	Kenya	IDPs, Not displaced	Camp	General population	HIV	ART, Behaviour change/education, Counselling	Healthcare, UN/NGO	Clinics, mobile clinics	Doctors, nurses, health workers
Hampton 2008[77]	Non-Research	Uganda	IDPs	Dispersed	General population	HIV	ART	UN/NGO	Home	Health workers
Hemhongsa 2008[81]	Observational	Thailand	Refugees, Not displaced	Camp	General population	HIV	ART, Antibiotics	Healthcare, UN/NGO	Hospitals, clinics	Doctors, nurses

Meteke S, *et al. BMJ Global Health* 2020; 5:e001967. doi: 10.1136/bmjgh-2019-001967

Kaiser 2006[89]	Observational	Sudan	IDPs	NR	Females (15-49y)	HIV, Syphilis	Behaviour change/education, Screening (for referral), Antibiotics, Counselling	UN/NGO	Clinics	NR
Kiboneka 2009[94]	Non-Research	Uganda	IDPs	Camp, Dispersed	General population (>15y)	HIV	ART	UN/NGO	Clinics, mobile clinics, home	Civic leaders, doctors, UN/NGO staff, field workers
Kim 2009[95]	Observational	DRC	IDPs, Returning refugees, Not displaced	Camp, Dispersed	Females (15-49y)	HIV, Syphilis	Behaviour change/education, Screening (for referral), Antibiotics	UN/NGO	Clinics	NR
Krause 2015[101]	Mixed methods		Refugees	Camp, Dispersed	General population	HIV	Behaviour change/education	Healthcare, UN/NGO	Hospitals, clinics	NR
Kruk 2010[102]	Observational	Liberia	Unreported	–	General population	HIV	Screening (for referral)	Healthcare, UN/NGO	Hospitals, clinics, health posts	Doctors, nurses, health workers
Mayaud 2001[115]	Observational	Tanzania	Host, Refugees	Camp	General population	HIV, Syphilis	Antibiotics, Behaviour change/education, Screening (for referral), Training	UN/NGO	Clinics	Health workers, trained volunteers
McGinn 2006[117]	Observational	Guinea	Refugees	Camp	Females (>15y)	HIV	Training	UN/NGO	NR	Teachers
McGready 2000[118]	RCT	Thailand	Refugees	Camp	Pregnant women	HIV, Malaria	ART, Malaria treatment	UN/NGO, Research	Clinics	Midwives
Mendelsohn 2014[120]	Observational	Malaysia	Host, Refugees	Dispersed	General population (>15y)	HIV	ART	Healthcare, UN/NGO	Hospitals	NR
Morrison 2000[122]	Mixed methods	Thailand	Refugees	Camp	Females (>10y)	HIV	Screening (for referral)	UN/NGO	Clinics	Birth attendants
Msuya 1996[123]	Non-Research	Tanzania	Refugees	Camp	General population	HIV, STIs	Behaviour change/education, Screening (for referral), Antibiotics	UN/NGO	Clinics, electronic/print	Birth attendants, health workers, peer health educators
O'Brien 2009[131]	Non-Research	Republic of Congo	Unreported	–	General population	HIV, Syphilis, Cryptococcosis	Antifungals, ART, Behaviour change/education, Antibiotics	Healthcare, UN/NGO	Hospitals, clinics	Doctors, nurses, health workers, counsellors
O'Laughlin 2014[132]	Observational	Uganda	Refugees, Not displaced	Camp, Dispersed	General population	HIV	Screening (for referral)	UN/NGO, Research	Clinics	UN/NGO staff
Plewes 2008[136]	Mixed methods	Thailand	Refugees	Camp	Pregnant women	HIV	Behaviour change/education, ART	UN/NGO, Research	Clinics	Counsellors
Reid 2008[149]	Non-Research	Kenya	Not displaced	–	General population	HIV	HIV prevention and treatment (call centre)	Healthcare, UN/NGO	Electronic print	UN/NGO staff

Meteke S, *et al. BMJ Global Health* 2020; 5:e001967. doi: 10.1136/bmjgh-2019-001967

Rodger 2002[154]	Observational	India	IDPs	Camp, Dispersed	General population	HIV, TB	Screening (for referral), DOTS, Nutrition intervention	Healthcare, UN/NGO	Clinics	Civic leaders, trained volunteers
Rosenberg 2017[155]	RCT	Uganda	Refugees	Camp, Dispersed	Females (>15y)	HIV	Screening (for referral), Training	UN/NGO	Clinics	UN/NGO staff
Rowley 2008[164]	Observational	Tanzania	Host, Refugees	Camp, Dispersed	General population (>15y)	HIV	Behaviour change/education, Screening (for referral)	UN/NGO	NR	UN/NGO staff
Rutta 2008[167]	Observational	Tanzania	Refugees	Camp	Pregnant women, Post-natal mothers	HIV	ART, Behaviour change/education	Healthcare, UN/NGO	Hospitals, clinics	CHWs, doctors, nurses, birth attendants, health workers
Salami 2010[169]	Observational	South Sudan	IDPs, Returning refugees	Camp, Dispersed	General population	HIV	ART	UN/NGO	Clinics	NR
Shamomesh 1994[175]	Observational	El Salvador	Returning refugees	Dispersed	Females (>15y)	HIV	Screening (for referral)	UN/NGO	Clinics	NR
Somigliana 2011[178]	Observational	Uganda	Unreported	–	Pregnant women	HIV	ART	UN/NGO	Hospitals, clinics	Doctors, health workers, traditional birth attendants
Tanaka 2008[182]	Mixed methods	Tanzania	Refugees	Camp	General population (>10y)	HIV	Behaviour change/education, Screening (for referral)	UN/NGO	Clinics, electronic/print	Trained volunteers
Tshipala 2012[187]	Observational	Côte d'Ivoire, Ghana, Liberia	IDPs, Refugees	Dispersed	General population	HIV	ART	NR	Clinics, health posts	NR
UNICEF 2016[184]	Non-Research	CAR	IDPs	Camp, Dispersed	Children (<5y), Pregnant women	HIV, Malaria, Measles	ART, Malaria prevention, Vaccination, Vitamin A	UN/NGO	NR	UN/NGO staff
von Roenne 2010[193]	Non-Research	Guinea	Host, Refugees	Camp, Dispersed	Females (15-49y)	HIV	Screening (for referral)	Healthcare, UN/NGO	Hospitals, clinics, health posts	Nurses
WHO/GPA 1994[72]	Non-Research	Rwanda	Refugees	Camp	General population	HIV	Behaviour change/education	UN/NGO	NR	NR
**Tuberculosis**										
Agutu 1997[6]	Observational	Somalia	Not displaced	–	General population	TB	DOTS	UN/NGO	Hospitals, clinics	Doctors, health workers
Ahmadzai 2008[7]	Observational	Afghanistan	Not displaced	–	General population	TB	DOTS	Healthcare, UN/NGO	Hospitals, clinics, mobile clinics, home	CHWs, doctors, nurses, health workers
Bam 2007[19]	Observational	Nepal	Refugees	Camp	General population	TB	DOTS	Healthcare, UN/NGO	Clinics	CHWs

Barr 1994[22]	Observational	El Salvador	IDPs, Returning refugees	Dispersed	General population	TB	Antibiotics, Screening (for referral)	UN/NGO	Communal spaces, home	Civic leaders, UN/NGO staff, trained volunteers
Bile 2011[26]	Non-Research	Pakistan	IDPs	Camp, Dispersed	General population	TB, Diarrhoeal diseases, Malaria, Measles	ORT, Malaria prevention, Malaria treatment, Vaccination, DOTS	NR	NR	NR
Bohler 2005[27]	Observational	Sudan	IDPs, Host	Camp, Dispersed	General population	TB	DOTS	Healthcare	Clinics, mobile clinics	NR
Cookson 2015[45]	Non-Research	Jordan	Refugees	Camp, Dispersed	General population	TB	Behaviour change/education, DOTS, Screening (for referral)	Healthcare, UN/NGO	Mobile clinics, communal spaces	UN/NGO staff, health workers
Dierberg 2016[51]	Observational	India	Refugees	Camp, Dispersed	General population	TB	Screening (for referral)	UN/NGO	Clinics, home, places of worship	Health workers, UN/NGO staff
Gorbacheva 2010[71]	Observational	Nepal	Refugees	Camp	General population	TB	DOTS, Screening (for referral)	UN/NGO	NR	UN/NGO staff
Hehenkamp 2003[79]	Non-Research	South Sudan	Not displaced	–	General population	TB	Antibiotics, DOTS	UN/NGO	Clinics	UN/NGO staff
Heldal 1997[80]	Observational	Nicaragua	Unreported	–	General population	TB	Antibiotics, DOTS	Healthcare, UN/NGO	Hospitals, clinics, health posts	Doctors, nurses
Jarallah 1993[87]	Observational	Pakistan	Refugees	Camp	General population (>1y)	TB	Antibiotics	UN/NGO	Clinics	Doctors
Keus 2003[93]	Observational	Sudan	Not displaced	–	General population	TB	Antibiotics, DOTS	UN/NGO	Clinics	Doctors, health workers, UN/NGO staff
Liddle 2013[105]	Observational	Somalia	IDPs	Dispersed	General population	TB	Antibiotics, Behaviour change/education, DOTS, Monthly nutrition package	Healthcare, UN/NGO	Hospitals, clinics	Doctors, nurses
Minetti 2010[121]	Observational	Thailand	Refugees, Not displaced	Camp, Dispersed	General population	TB	DOTS	UN/NGO	Clinics	CHWs
Rodger 2002[154]	Observational	India	IDPs	Camp, Dispersed	General population	TB, HIV	Screening (for referral), DOTS, Nutrition intervention	Healthcare, UN/NGO	Clinics	Civic leaders, trained volunteers
Rutta 2001[168]	Observational	Tanzania	Refugees	Camp	General population	TB	DOTS	Healthcare, UN/NGO	Clinics, home	CHWs, Nurses
Sukrakanchana-Trikham 1992[180]	Observational	Thailand	Refugees	Camp	General population	TB	DOTS	UN/NGO	Clinics	Nurses

Meteke S, *et al. BMJ Global Health* 2020; 5:e001967. doi: 10.1136/bmjgh-2019-001967

**Neglected Tropical Diseases (NTDs)**										
Abdullah 2016[3]	Non-Research	Yemen	IDPs	Dispersed	Children (<5y)	Worm infestation	Deworming	UN/NGO	NR	NR
Al-Kamel 2016[9]	Observational	Yemen	IDPs, Not displaced	Dispersed	General population	Leishmaniasis	Parasitic treatment	UN/NGO	Health posts, home	UN/NGO staff, trained volunteers
Boru 2013[28]	Observational	Kenya	Refugees	Dispersed	Children (<9y)	Worm Infestation, Diarrhoeal Diseases, Malaria	ORT, Malaria treatment, Deworming	Healthcare	Clinics	NR
Bustami 2010[33]	Non-randomized CT	Jordan	Refugees	Camp	Children, Adolescents (<19y)	Worm Infestation	Behaviour change/education, Deworming, Screening (for referral), Deworming	NR	Clinics, home	NR
Gelaw 2015[67]	Observational	Ethiopia	Refugees	Camp	General population	Trachoma	Antibiotics, Behaviour change/education, Screening (for referral), Surgical management	UN/NGO, Research	Clinics, mobile clinics	UN/NGO staff, researchers
Javaloy 2003[88]	Observational	Algeria	Refugees	Camp	Children, Adolescents (<19y)	Trachoma	Antibiotics, Screening (for referral)	Research	Home, schools	UN/NGO staff
Sallam 2018[170]	Non-Research	Yemen	Not displaced	–	Children (<5y), Pregnant women	Worm infestation	Deworming, Training	Healthcare, UN/NGO	NR	UN/NGO staff
Saroufim 2014[173]	Observational	Lebanon	Refugees	Camp	General population	Leishmaniasis	Parasitic treatment	Healthcare, UN/NGO, Research	NR	UN/NGO staff, researchers, health workers
Seaman 1996[174]	Observational	Sudan	Not displaced	–	General population	Leishmaniasis	Antimonial therapy	UN/NGO	Clinics	UN/NGO staff, trained volunteers
Witzig 2015[194]	Observational	Syria	Not displaced	–	General population	Leishmaniasis	Antifungals, Parasitic treatment and Deworming	NR	Clinics	NR
**Other Infections**										
Raoult 1998[148]	Observational	Burundi	IDPs	Camp	General population	Typhus	Antibiotics	Healthcare	Clinics	CHWs
Reynolds 2013[150]	Non-Research	Republic of Congo	Refugees	Camp, Dispersed	General population	Monkeypox	Behaviour change/education	UN/NGO	Communal spaces	UN/NGO staff

Meteke S, *et al. BMJ Global Health* 2020; 5:e001967. doi: 10.1136/bmjgh-2019-001967

Appendix C. Summary of quantitative data on intervention coverage and effectiveness, by displacement status and setting

Author, Year	Intervention	Outcome Description	Sample size	Point Estimate (95% CI)	Displacement Status	Setting
**Coverage**						
Ciglenecki 2015[42]	Cholera vaccine	Proportion of individuals >1y (excluding pregnant women) who received at least one dose of oral cholera vaccine during mass immunization campaign in camp	NR	84.1 (81.5, 86.3)	IDPs	Camp
Ciglenecki 2015[42]	Cholera vaccine	Proportion of individuals >1y (excluding pregnant women) who received two doses of oral cholera vaccine with during mass immunization campaign in camp	NR	65.5 (61.2, 69.6)	IDPs	Camp
Lam 2017[103]	Cholera vaccine	Proportion of children 1-4y who received 2 doses of cholera oral vaccine during a national vaccination campaign in 2015	407	85.0 (81.0, 88.0)	IDPs, Refugees	Camp
Lam 2017[103]	Cholera vaccine	Proportion of children 5-14y who received 2 doses of cholera oral vaccine during a national vaccination campaign in 2015	931	89.0 (85.0, 92.0)	IDPs, Refugees	Camp
Lam 2017[103]	Cholera vaccine	Proportion of females >1y who received 2 doses of cholera oral vaccine during a national vaccination campaign in 2015	1,777	88.0 (85.0, 90.0)	IDPs, Refugees	Camp
Lam 2017[103]	Cholera vaccine	Proportion of women >15y who received 2 doses of cholera oral vaccine during a national vaccination campaign in 2015	1,143	88.0 (84.0, 91.0)	IDPs, Refugees	Camp
Garenne 1997[65]	DTP vaccination	Proportion of children 1-5y who received all vaccinations during an EPI (BCG, DPT, OPV, and measles) that began in 1991.	NR	41.0	Not displaced	—
Vitek 20001[92]	DTP vaccination	Proportion of children in grades 1-5 that were vaccinated in a mass immunization campaign targeting school-aged children for a single dose of diphtheria toxoid December 1994	NR	96.0	Unreported	—
Vitek 20001[92]	DTP vaccination	Proportion of children in grades 6-11 that were vaccinated in a mass immunization campaign targeting school-aged children for a single dose of diphtheria toxoid May 1995	NR	98.0	Unreported	—
Augusto 2015[16]	HIV prevention & treatment	Proportion of HIV exposed infants (HEI) identified through PMTCT services who received antiretroviral prophylaxis nationally in 2012	NR	13.0	Not displaced	—
Augusto 2015[16]	HIV prevention & treatment	Proportion of pregnant women who were tested for HIV at PMTCT sites nationally in 2005	12,061	1.6	Not displaced	—
Augusto 2015[16]	HIV prevention & treatment	Proportion of pregnant women who were tested for HIV at PMTCT sites nationally in 2012	314,805	33.7	Not displaced	—
Augusto 2015[16]	HIV prevention & treatment	Proportion of HIV positive pregnant women who received antiretroviral prophylaxis in 2012	NR	17.0	Not displaced	—
Doumbouya 2012[53]	HIV prevention & treatment	Proportion of HIV-positive children who received antiretroviral therapy	17,471	8.7	Not displaced	—
Kim 2009[95]	HIV & syphilis screening	Proportion of women 15-49y living in river communities along the Congo River who participated in a referral vouchers for free voluntary counseling and HIV and Syphilis testing (VCT) services at temporary VCT sites program.	NR	20.0	Not displaced	Dispersed
Kim 2009[95]	HIV & syphilis screening	Proportion of women 15-49y living in IDP camps who participated in a referral vouchers for free voluntary counseling and HIV and Syphilis testing (VCT) services at temporary VCT sites program.	NR	90.0	IDPs	Camp
Obol 2013[130]	Malaria prevention	Proportion of pregnant women utilizing ITNs for prevention of malaria (across 31 camps)	769	35.0 (31.0, 38.0)	IDPs	Camp
Richards 2009[151]	Malaria prevention	Proportion of children < 5y that were reached by a community based integrated malaria control program in 2004	3,457	17.0	IDPs	Dispersed

Meteke S, *et al. BMJ Global Health* 2020; 5:e001967. doi: 10.1136/bmjgh-2019-001967

Coldiron 2017[43]	Malaria treatment	Proportion of children (6m-15y) who adhered to malaria treatment in refugee camps in Northern Uganda	NR	90.0	Hosts, Refugees	Camp
Kajeechiwa 2016[90]	Malaria treatment	Proportion of Female Participants who participated in a targeted malaria elimination project and completed all three rounds of mass drug administrations (MDA) of dihydroartemisinin/piperaquine plus a single low dose primaquine (anti-malarial drugs)	174	72.0	Refugees	Dispersed
Ciglenecki 2015[42]	Measles vaccine	Proportion of children <5 who received mass measles vaccine during mass vaccination campaign in camp	NR	73.9 (68.8, 78.3)	IDPs	Camp
de Lima Pereira 2018[48]	Measles vaccine	Proportion of children 6-59m who received administrative measles vaccination coverage	4,800	71.0	Unreported	—
de Lima Pereira 2018[48]	Measles vaccine	Proportion of children 6-59m who received measles vaccination during the post supplementary immunization activities	280	81.8 (76.9, 85.9)	Unreported	—
Elsayed 2004[57]	Measles vaccine	Proportion of children 9m-15y who received measles vaccine during mass campaign in North Darfur in 2004	657,774	75.0	IDPs	Camp, Dispersed
Elsayed 2004[57]	Measles vaccine	Proportion of children 9m-15y who received measles vaccine during mass campaign in South Darfur in 2004	1,260,324	97.0	IDPs	Camp, Dispersed
Elsayed 2004[57]	Measles vaccine	Proportion of children 9m-15y who received measles vaccine during mass campaign in West Darfur in 2004	688,984	44.0	IDPs	Camp, Dispersed
Elsayed 2004[57]	Measles vaccine	Proportion of children 9m-15y who received measles vaccine during mass campaign in all regions of Darfur (total) in 2004	2,607,082	77.0	IDPs	Camp, Dispersed
Feroz 2003[63]	Measles vaccine	Proportion of children aged 6m-12y who received a Measles vaccine during a Nationwide Measles vaccination campaign in 2002	12,491,152	82.0	Returning Refugees, Not displaced	Dispersed
Koscalova 2014[99]	Measles vaccine	Proportion of children who received measles vaccine in children (6m-15y) in Buta, DRC	NR	88.6 (82.2, 92.9)	Unreported	—
Koscalova 2014[99]	Measles vaccine	Proportion of children who received measles vaccine in children (6m-15y) in Ganga-Dingila, DRC	NR	90.5 (86.2, 93.5)	Unreported	—
Koscalova 2014[99]	Measles vaccine	Proportion of children who received measles vaccine in children (6m-15y) in Aketi, DRC	NR	97.7 (97, 98.4)	Unreported	—
Koscalova 2014[99]	Measles vaccine	Proportion of children who received measles vaccine in children (6m-15y) in Bondo, DRC	NR	89.6 (86.8, 92.5)	Unreported	—
Koscalova 2014[99]	Measles vaccine	Proportion of children who received measles vaccine in children (6m-15y) in Likati, DRC	NR	93.4 (90.8, 95.9)	Unreported	—
WHO 2004[140]	Measles vaccine	Proportion of children 9m-15y who received measles vaccine during mass immunization campaign	2,607,082	77.0	IDPs, Not displaced	Camp
Ciglenecki 2015[42]	Meningitis vaccine	Proportion of individuals aged 1-30y who received meningicoccal A (MenA) vaccines	NR	77.3 (73.5, 80.8)	IDPs	Camp
Aaby 2005[2]	Polio vaccine	Proportion of children <5y who received 1 or 2 doses of polio vaccines during mass immunization campaign in Bandim, Belem and Mindara	6,103	82.0	IDPs, Not displaced	Dispersed
CDC 1999[36]	Polio vaccine	Proportion of children <1y who received three doses of oral polio vaccine nationally in 1996	NR	30.0	Unreported	—
CDC 1999[36]	Polio vaccine	Proportion of children <5y who received 2 doses of oral polio vaccine during national immunization days in 1997	NR	80.0	Unreported	—
Habib 2017[75]	Polio vaccine	Proportion of children (1-59m) who received OPV during routine polio programme activities	28,760	75.0 (74.0, 77.0)	Unreported	—

Habib 2017[75]	Polio vaccine	Proportion of children (1-59m) who received OPV and additional interventions with community outreach/mobilization using enhanced communication packages	30,098	82.0 (81.0, 83.0)	Unreported	—
Habib 2017[75]	Polio vaccine	Proportion of children (1-59m) who received OPV, in addition to IPV and other interventions using enhanced communication packages	29,126	84.0 (83.0, 85.0)	Unreported	—
Makokha 2014[112]	Polio vaccine	Proportion of children <5y who received both oral and inactivated polio vaccine in refugee camps during the December campaign	1,568	92.8 (90.2, 94.8)	Hosts, Refugees	Camp, Dispersed
Makokha 2014[112]	Polio vaccine	Proportion of children <5y who received both oral and inactivated polio vaccine in host communities during the December campaign	593	95.8 (93.5, 97.3)	Hosts, Refugees	Camp, Dispersed
Makokha 2014[112]	Polio vaccine	Proportion of children <5y who received oral polio vaccine only in refugee camps during the December campaign	1,534	97.2 (95.4, 98.3)	Hosts, Refugees	Camp, Dispersed
Makokha 2014112[116]	Polio vaccine	Proportion of children <5y who received oral polio vaccine only in host communities during the December campaign	590	97.3 (95, 98.5)	Hosts, Refugees	Camp, Dispersed
Mbaeyi 2017[116]	Polio vaccine	Proportion of children 6-59m who received oral polio vaccine during national immunization days in December 2013	NR	79.0	Unreported	—
Mbaeyi 2017[116]	Polio vaccine	Proportion of children 6-59m who received oral polio vaccine during national immunization days in March 2014	NR	93.0	Unreported	—
Shuaibu 2016[176]	Polio vaccine	Proportion of children <5y who received oral polio vaccine after immunization campaign in Borno	685,674	105.1	IDPs, Not displaced	Camp, Dispersed
Shuaibu 2016[176]	Polio vaccine	Proportion of children <5y who received oral polio vaccine after immunization campaign in Yobe	113,774	103.3	IDPs, Not displaced	Dispersed
Shuaibu 2016[176]	Polio vaccine	Proportion of children 14w-5y who received inactivated polio vaccine after immunization campaign in Borno	608,964	102.9	IDPs, Not displaced	Camp, Dispersed
Shuaibu 2016[176]	Polio vaccine	Proportion of children 14w-5y who received inactivated polio vaccine after immunization campaign in Yobe	111,570	94.9	IDPs, Not displaced	Camp, Dispersed
WHO 2000[146]	Polio vaccine	Proportion of children <5y who received first round of oral polio vaccine in 11 DRC provinces	10,462,289	71.0	Not displaced	—
WHO 2000[146]	Polio vaccine	Proportion of children <5y who received second round of oral polio vaccine in 11 DRC provinces	10,462,289	86.0	Not displaced	—
WHO 2000[146]	Polio vaccine	Proportion of children <5y who received third round of oral polio vaccine in 11 DRC provinces	10,462,289	81.0	Not displaced	—
WHO 2014[44]	Polio vaccine	Proportion of children <5y who received oral and intramuscular polio vaccine during national immunization in December 2013	2,161	93.3 (91.2, 95)	Hosts, Refugees	Camp, Dispersed
WHO 2014[44]	Polio vaccine	Proportion of children <5y who received oral and intramuscular polio vaccine during national immunization in November 2013	2,124	97.2 (95.4, 98.3)	Hosts, Refugees	Camp, Dispersed
Cookson 2015[45]	TB screening	Proportion of children <15y screened for tuberculosis among Syrian refugees in camps	68,906	45.0	Refugees	Camp, Dispersed
Kline 1999[97]	TB vaccine	Proportion of children <4y who received age-appropriate vaccines among those targeted according to MoH immunization schedule in Bojane	233	92.0	Refugees	Camp
Kline 1999[97]	TB vaccine	Proportion of children <4y who received age-appropriate vaccines among those targeted according to MoH immunization schedule in Brazda	2,547	93.0	Refugees	Camp
Kline 1999[97]	TB vaccine	Proportion of children <4y who received age-appropriate vaccines among those targeted according to MoH immunization schedule in Cegrane	2,928	89.0	Refugees	Camp
Kline 1999[97]	TB vaccine	Proportion of children <4y who received age-appropriate vaccines among those targeted according to MoH immunization schedule in Senokos	314	98.0	Refugees	Camp

Kline 1999[97]	TB vaccine	Proportion of children <4y who received age-appropriate vaccines among those targeted according to MoH immunization schedule in Stankovec	1,431	78.0	Refugees	Camp
Garenne 1997[65]	Tetanus vaccine	Proportion of women 15y-45y who had given birth over the past 12 months in 1994 that had received at least one dose of Tetanus toxoid.	NR	82.0	Not displaced	—
Garenne 1997[65]	Tetanus vaccine	Proportion of women 15y-45y who had given birth over the past 12 months in 1994, that had received two doses of tetanus toxoid at least 15 days before delivery	NR	35.0	Not displaced	—
**Effectiveness**						
Bustami 2010[33]	Deworming	Proportion of children (5-15y) recovered after 1st dose mebendazole treatment	NR	23.3	Refugees	Camp
Bustami 2010[33]	Deworming	Proportion of children (5-15y) recovered after 1st dose mebendazole treatment + weekly enema & health education	NR	81.6	Refugees	Camp
Burns 2012[32]	Malaria prevention	Malaria Incidence Rate Ratio among children from Largo camp treated with Insectiside treated plastic sheeting	51	60.0 (53.0, 67.0)	Refugees	Camp
Burns 2012[32]	Malaria prevention	Malaria Incidence Rate Ratio among children from Largo camp with untreated polyethylene sheeting	49	1.0	Refugees	Camp
Burns 2012[32]	Malaria prevention	Malaria Incidence Rate Ratio among children from Tobanda camp with Insectiside treated plasting sheeting	55	0.85 (0.81, 0.89)	Refugees	Camp
Burns 2012[32]	Malaria prevention	Malaria Incidence Rate Ratio among children from Tobanda camp with untreated polyethylene sheeting	67	1.00	Refugees	Camp
Kimani 2006[96]	Malaria prevention	OR of malaria infection among youth 15-24y with use of insecticide-treated clothing	NR	0.09	Refugees	Camp
Kimani 2006[96]	Malaria prevention	OR of malaria infection among children 5-14y with use of insecticide-treated clothing	NR	0.24	Refugees	Camp
Kimani 2006[96]	Malaria prevention	OR of malaria infection among children <5y with use of insecticide-treated clothing	NR	0.56	Refugees	Camp
Spencer 2004[179]	Malaria prevention	Relative risk of having malarial parasitaemia among ITNs users compared to non-users	578	0.67 (0.43, 1.03)	IDPs	Camp
Spencer 2004[179]	Malaria prevention	Proportion of children <4y having malarial parasitaema among ITN users	331	10.3	IDPs	Camp
Spencer 2004[179]	Malaria prevention	Proportion of children <4y having malarial parasitaema among non-users of ITN	247	14.6	IDPs	Camp
Depoortere 2005[50]	Malaria treatment	Proportion of children <5y with adequate clinical & parasitological response at day 14 in the supervised arm (efficacy)	82	86.6 (77.9, 92.7)	Refugees	Camp
Depoortere 2005[50]	Malaria treatment	Proportion of children <5y with adequate clinical & parasitological response at day 14 in the unsupervised arm (effectiveness)	82	71.9 (61.5, 80.9)	Refugees	Camp
Depoortere 2005[50]	Malaria treatment	Proportion of children <5y with adequate clinical & parasitological response at day 28 in the supervised arm (efficacy)	79	83.5 (74.1, 90.5)	Refugees	Camp
Depoortere 2005[50]	Malaria treatment	Proportion of children <5y with adequate clinical & parasitological response at day 28 in the unsupervised arm (effectiveness)	82	63.4 (52.6, 73.3)	Refugees	Camp
Howard 2011[83]	Malaria treatment	Proportion of children (6–14y) who did not respond to First line Treatment (CQ 25mg dose)	111	86.0	Refugees	Camp
Howard 2011[83]	Malaria treatment	Proportion of children (6–14y) who did not respond to First line Treatment (CQ 40mg dose)	111	55.0	Refugees	Camp
Howard 2011[83]	Malaria treatment	Proportion of children (6–14y) who did not respond to Second line Treatment (CQ 25mg dose)	62	84.0	Refugees	Camp

Meteke S, *et al. BMJ Global Health* 2020; 5:e001967. doi: 10.1136/bmjgh-2019-001967

Howard 2011[83]	Malaria treatment	Proportion of children (6–14y) who did not respond to Second line Treatment (CQ 40mg dose)	62	75.0	Refugees	Camp
Howard 2011[83]	Malaria treatment	Proportion of children (6m–5y) who did not respond to First line Treatment (CQ 25mg dose)	111	89.0	Refugees	Camp
Howard 2011[83]	Malaria treatment	Proportion of children (6m–5y) who did not respond to First line Treatment (CQ 40mg dose)	111	61.0	Refugees	Camp
Howard 2011[83]	Malaria treatment	Proportion of children (6m–5y) who did not respond to Second line Treatment (CQ 25mg dose)	62	93.0	Refugees	Camp
Howard 2011[83]	Malaria treatment	Proportion of children (6m–5y) who did not respond to Second line Treatment (CQ 40mg dose)	62	62.0	Refugees	Camp
McGready 2000[118]	Malaria treatment	Proportion of pregnant women cured with a mefloquine-artesunate regimen at follow up	65	98.2 (94.7, 100)	Refugees	Camp
McGready 2000[118]	Malaria treatment	Proportion of pregnant women cured with a quninine regimen at follow-up	41	67 (43.3, 90.8)	Refugees	Camp
Smithuis 1993[177]	Malaria treatment	Proportion of children 1-15y who failed to respond to therapy M15 after 42 days	11	73.0	Refugees	Camp
Smithuis 1993[177]	Malaria treatment	Proportion of children 1-15y who failed to respond to therapy M25 after 42 days	15	53.0	Refugees	Camp
Ter Kuile 1993[183]	Malaria treatment	Failure rates with halofantrine 24 mg/kg in children <6y compared to older children and adults	27	59.0	Refugees	Camp
Nosten 1999[128]	Malaria vaccine	Proportion of children with Symptomatic falciparum Malaria after receiving all 3 doses of the vaccination SPf66- Intervention arm	610	51.5	Refugees	Camp
Nosten 1999[128]	Malaria vaccine	Proportion of children with Symptomatic falciparum Malaria after receiving Recombinant hepatitis B vaccine- Control arm	611	48.5	Refugees	Camp
Nosten 1999[129]	Malaria vaccine	Vaccine efficacy (proportionate reduction in malaria incidence among the vaccinated) in children 2-15y	1221	–9.0 (–33.0, 14.0)	Refugees	Camp
Porter 1990[139]	Measles vaccine	Vaccine efficacy (proportionate reduction in measles incidence among the vaccinated) in children 6-59m in all 5 camps	NR	94.0	Refugees	Camp
Porter 1990[139]	Measles vaccine	Vaccine efficacy (proportionate reduction in measles incidence among the vaccinated) in children 6m-9m in all 5 camps (protection)	NR	93.0	Refugees	Camp
Mayaud 2001[115]	Syphilis screening	Percent decrease in reproductive tract infections at time of second survey among pregnant women attending antenatal clinics	NR	25.0	Hosts, Refugees	Camp
Liddle 2013[105]	TB DOTS	OR of having successful DOTS treatment outcome (cure or complete) among patients 1-5y compared to patients >15y	2717	1.03 (0.68, 1.57)	IDPs	Dispersed
Liddle 2013[105]	TB DOTS	OR of having successful DOTS treatment outcome (cure or complete) among patients 5-15y compared to patients >15y	2717	1.34 (0.83, 2.15)	IDPs	Dispersed
Liddle 2013[105]	TB DOTS	OR of having successful DOTS treatment outcome (cure or complete) among patients (<1y) compared to patients >15y	2717	0.28 (0.2, 0.4)	IDPs	Dispersed

Included Publications Citation List

1. Aaby P, Garly ML, Bale C, et al. Survival of previously measles-vaccinated and measles-unvaccinated children in an emergency situation: An unplanned study. *Pediatr Infect Dis J*. 2003;22(9):798-805.
2. Aaby P, et al. Childhood mortality after oral polio immunisation campaign in Guinea-Bissau. *Vaccine*. 2005;23(14):1746-51.
3. Abdullah S, Al Ardi R, R S. Scaling up nutrition services and maintaining service during conflict in Yemen: lessons from the Hodeiadah sub-national nutrition cluster. New York, NY: UNICEF; 2016.
4. Adam IF, Nakamura K, Kizuki M, et al. Relationship between implementing interpersonal communication and mass education campaigns in emergency settings and use of reproductive healthcare services: Evidence from Darfur, Sudan. *BMJ Open*. 2015;5(9):e008285.
5. Adam I. Evidence from cluster surveys on the association between home-based counseling and use of family planning in conflict-affected Darfur. *International Journal of Gynecology and Obstetrics*. 2016;133:221-5.
6. Agutu, WO. Short-course tuberculosis chemotherapy in rural Somalia. *East Afr Med J*. 1997;74:348-52.
7. Ahmadzai H, et al. Scaling up TB DOTS in a fragile state: post-conflict Afghanistan. *Int J Tuberc Lung Dis*. 2008;12(2):180-5.
8. Ahoua L, A T, Duroch F, et al. High mortality in an internally displaced population in Ituri, Democratic Republic ofCongo, 2005: Results of a rapid assessment under difficult conditions. *Global Public Health*. 2006;1:195-204.
9. Al-Kamel MA. Impact of leishmaniasis in women: a practical review with an update on my ISD-supported initiative to combat leishmaniasis in Yemen (ELYP). *International Journal of Women's Dermatology*. 2016;2(3):93-101.
10. Alleman MM, Chitale R, Burns CC, et al. Vaccine-derived polioviruses outbreaks and events in 3 provinces of Democratic Republic of the Congo, 2017. *WHO Weekly epidemiological record*. 2018;93:117-32.
11. Ambler MT, et al. The neurological assessment in young children treated with artesunate monotherapy or artesunate-mefloquine combination therapy for uncomplicated Plasmodium falciparum malaria. *Malar J*. 2009;8:207.
12. Apiyo R. Regional supply hub mechanism as a strategy for wash emergency response in Somalia. Nairobi, Kenya UNICEF; 2014.
13. Ashley EA, Krudsood S, Phaiphun L, et al. Randomized, controlled dose-optimization studies of dihydroartemisinin-piperaquine for the treatment of uncomplicated multidrug-resistant falciparum malaria in Thailand. *The Journal of infectious diseases*. 2004;190(10):1773-82.
14. Ashley EA, Lwin KM, McGready R, et al. An open label randomized comparison of mefloquine-artesunate as separate tablets vs. a new co-formulated combination for the treatment of uncomplicated multidrug-resistant falciparum malaria in Thailand. *Tropical medicine & international health : TM & IH*. 2006;11(11):1653-60.
15. Ashley EA, McGready R, Hutagalung R, et al. A randomized, controlled study of a simple, once-daily regimen of dihydroartemisinin-piperaquine for the treatment of uncomplicated, multidrug-resistant falciparum malaria. *Clinical infectious diseases : an official publication of the Infectious Diseases Society of America*. 2005;41(4):425-32.
16. Augusto GF. Use of services for prevention of mother-to-child transmission in Angola: a retrospective analysis. *Journal of public health (Oxford, England)*. 2016;38(2):371-7.
17. Azman AS, Parker LA, Rumunu J, et al. Effectiveness of one dose of oral cholera vaccine in response to an outbreak: a case-cohort study. *The Lancet Global health*. 2016;4(11):e856-e63.
18. Azman AS, Rumunu J, Abubakar A, et al. Population-level effect of cholera vaccine on displaced populations, South Sudan, 2014. *Emerg Infect Dis*. 2016;22(6):1067-70.
19. Bam TS, Enarson DA, Hinderaker SG, et al. High success rate of TB treatment among Bhutanese refugees in Nepal. *Int J Tuberc Lung Dis*. 2007;11(1):54-8.
20. Banks T, Khang J, Watts I, et al. High hepatitis B seroprevalence and risk factors for infection in pregnant women on the Thailand-Myanmar border. *Journal of Infection in Developing Countries*. 2016;10(4):377-83.
21. Bannink-Mbazzi F, Lowicki-Zucca M, Ojom L , et al. High PMTCT Program Uptake and Coverage of Mothers, Their Partners, and Babies in Northern Uganda: Achievements and Lessons Learned Over 10 Years of Implementation (2002–2011). *J Acquir Immune Defic Syndr*. 2013;62:138-45.
22. Barr RG, Menzies R. The effect of war on tuberculosis: Results of a tuberculin survey among displaced persons in El Salvador and a review of the literature. *Tuber Lung Dis*. 1994;75(4):251-9.
23. Bekolo CE, van Loenhout JAF, Rodriguez-Llanes JM, et al. A retrospective analysis of oral cholera vaccine use, disease severity and deaths during an outbreak in South Sudan. *Bull World Health Organ*. 2016;94(9):667-74.
24. Benjamin JA. AIDS prevention for refugees. The case of Rwandans in Tanzania. *Aidscaptions*. 1996;3(2):4-9.
25. Benny E, Mesere K, Pavlin BI, et al. A large outbreak of shigellosis commencing in an internally displaced population, Papua New Guinea, 2013. *Western Pacific surveillance and response journal : WPSAR*. 2014;5(3):18-21.
26. Bile KM, Shadoul AF, Raajimakers H, et al. Learning through crisis: development and implementation of a health cluster strategy for internally displaced persons. *Eastern Mediterrnean Health Journal*. 2010;16:82-90.
27. Bohler M, Mustafaa SA, Morkve O. Tuberculosis treatment outcome and health services: a comparison of displaced and settled population groups in Khartoum, Sudan. *The international journal of tuberculosis and lung disease : the official journal of the International Union against Tuberculosis and Lung Disease*. 2005;9(1):32-6.
28. Boru WG, Kikuvi G, Omollo J, et al. Aetiology and factors associated with bacterial diarrhoeal diseases amongst urban refugee children in Eastleigh, Kenya: A case control study. *African Journal of Laboratory Medicine*. 2013;2(1):no pagination.
29. Bouma MJ, Parvez SD, Nesbit R, et al. Malaria control using permethrin applied to tents of nomadic Afghan refugees in northern Pakistan. *Bull World Health Organ*. 1996;74(4):413-21.
30. Brooks HM, Paul MKJ, Claude KM, et al. Use and disuse of malaria bed nets in an internally displaced persons camp in the Democratic Republic of the Congo: A mixed-methods study. *PLoS One*. 2017;12(9):e0185290.
31. Brown V, Reilley B, Ferrir MC, et al. Cholera outbreak during massive influx of Rwandan returnees in November, 1996. *Lancet*. 1997;349(9046):212.
32. Burns M, Rowland M, N'Guessan R, et al. Insecticide-treated plastic sheeting for emergency malaria prevention and shelter among displaced populations: An observational cohort study in a refugee setting in Sierra Leone. *Am J Trop Med Hyg*. 2012;87(2):242-50.
33. Bustami F, Khraisha S. Enterobius vermicularis infection in three refugee camps in Jordan. *Jordan Medical Journal*. 2010;44(4):432-6.
34. Carrara VI, Sirilak S, Thonglairuam J, et al. Deployment of early diagnosis and mefloquine-artesunate treatment of falciparum malaria in Thailand: The Tak Malaria Initiative. *PLoS Med*. 2006;3(6):0856-64.
35. Casey SE, Larsen MM, McGinn T, et al. Changes in HIV/AIDS/STI knowledge, attitudes, and behaviours among the youth in Port Loko, Sierra Leone. *Global public health*. 2006;1(3):249-63.

36. CDC. Progress toward poliomyelitis eradication--Afghanistan, 1994-1999. *MMWR Morbidity and mortality weekly report*. 1999;48(37):825-8.
37. CDC. Progress toward poliomyelitis eradication during armed conflict--Somalia and southern Sudan, January 1998-June 1999. United States; 1999. Contract No.: 29.
38. CDC. From the Centers for Disease Control and Prevention. Outbreak of poliomyelitis--Angola, 1999. United States; 1999. Contract No.: 23.
39. Cetorelli V. The impact of the Iraq War on neonatal polio immunisation coverage: a quasi-experimental study. *J Epidemiol Community Health*. 2015;69(3):226-31.
40. Charchuk R, Houston S, Hawkes MT. Elevated prevalence of malnutrition and malaria among school-aged children and adolescents in war-ravaged South Sudan. *Pathogens and Global Health*. 2015;109(8):395-400.
41. Charlwood JD, Qassim M, Elnsur EI, et al. The impact of indoor residual spraying with malathion on malaria in refugee camps in eastern Sudan. *Acta Trop*. 2001;80(1):1-8.
42. Ciglenecki I, Masson S, Peyraud N, et al. Vaccinations in acute humanitarian emergencies: Minkamman, Lakes State, South Sudan. *Trop Med Int Health*. 2015;20((Masson, Luquero) Epicentre, Paris, France):367.
43. Coldiron ME, Lasry E, Bouhenia M, et al. Intermittent preventive treatment for malaria among children in a refugee camp in Northern Uganda: lessons learned. *Malar J*. 2017;16(1):218.
44. WHO. Combined use of inactivated and oral poliovirus vaccines in a large-scale campaign in refugee camps and host communities - Kenya, December 2013. 2014. Contract No.: 12.
45. Cookson ST, Abaza H, Clarke KR, et al. Impact of and response to increased tuberculosis prevalence among Syrian refugees compared with Jordanian tuberculosis prevalence: case study of a tuberculosis public health strategy. *Conflict and health*. 2015;9(101286573):18.
46. Cossa HA, Gloyd S, Vaz RG, et al. Syphilis and HIV infection among displaced pregnant women in rural Mozambique. *Int J STD AIDS*. 1994;5(2):117-23.
47. Culbert H TDOBDPETMCFNATCKVS. HIV treatment in a conflict setting: outcomes and experiences from Bukavu, Democratic Republic of the Congo. *PLoS Med*. 2007;4:e129.
48. de Lima Pereira A, Southgate R, Ahmed H, et al. Infectious Disease Risk and Vaccination in Northern Syria after 5 Years of Civil War: The MSF Experience. *PLoS currents*. 2018;10(101515638).
49. Depoortere E, Guthmann JP, Sipilanyambe N, et al. Adherence to the combination of sulphadoxine-pyrimethamine and artesunate in the Maheba refugee settlement, Zambia. *Trop Med Int Health*. 2004;9(1):62-7.
50. Depoortere E, Guthmann JP, Presse J, et al. Efficacy and effectiveness of the combination of sulfadoxine/pyrimethamine and a 3-day course of artesunate for the treatment of uncomplicated falciparum malaria in a refugee settlement in Zambia. *Trop Med Int Health*. 2005;10(2):139-45.
51. Dierberg KL, Dorjee K, Salvo F, et al. Improved detection of tuberculosis and multidrug-resistant tuberculosis among tibetan refugees, India. *Emerg Infect Dis*. 2016;22(3):463-8.
52. Dolan G, ter Kuile FO, Nosten F, et al. Halofantrine versus mefloquine in treatment of multidrug-resistant falciparum malaria. *Lancet (London, England)*. 1993;341(8852):1044-9.
53. Doumbouya B, Zaho M, Adouko S, et al. The challenge of maintaining continuum of care and support to PLHIV in health facilities located in military conflict zones in Ivory Coast. *J Int AIDS Soc*. 2012;15((Toure) ACONDA VS CI, Poject Executive Direction, Abidjan, Cote D'Ivoire):267.
54. Duroch FS-H, C Care for victims of sexual violence, an organization pushed to its limits: The case of Médecins Sans Frontières. International Review of the Red Cross; 2014.
55. Ehui E, Couitchere LS, Kouakou GA, et al. Antiretroviral chemoprophylaxis in children and adolescents victims of rape in Abidjan. *Med Mal Infect*. 2015;45(8):324-7.
56. Ellman T, Culbert H, Torres-Feced V. Treatment of AIDS in conflict-affected settings: a failure of imagination. *Lancet*. 2005;365 North American Edition(9456):278-80.
57. Elsayed A, N M. Emergency Measles Control Activities— Darfur, Sudan, 2004. *CDC MMWR*. 2004;53:897-9.
58. Erickson M, Goldenberg SM, Akello M, et al. Structural determinants of dual contraceptive use among female sex workers in conflict-affected Gulu, Northern Uganda. *Canadian Journal of Infectious Diseases and Medical Microbiology*. 2015;26((Akello, Muzaaya) KampalaUganda):83B.
59. UNICEF. Evaluation of Social Mobilization Network (SMNet) - Final Report. New York, NY: UNICEF; 2014.
60. WHO. Expanded programme on immunization (EPI). Poliomyelitis outbreak. Switzerland; 1994. Contract No.: 14.
61. Ezard N, Burns M, Lynch C, et al. Efficacy of chloroquine in the treatment of uncomplicated Plasmodium falciparum infection in East Timor, 2000. *Acta Trop*. 2003;88:87-90.
62. Fabiani M, Nattabi B, Opio AA, et al. A high prevalence of HIV-1 infection among pregnant women living in a rural district of north Uganda severely affected by civil strife. *Trans R Soc Trop Med Hyg*. 2006;100(6):586-93.
63. Feroz F, Sherazi A, Ashgar A. Nationwide measles vaccination campaign for children aged 6 months-12 years--Afghanistan, 2002. United States; 2003. Contract No.: 16.
64. Garang PG, Odoi RA, Kalyango JN. Adherence to antiretroviral therapy in conflict areas: a study among patients receiving treatment from Lacor Hospital, Uganda. *AIDS Patient Care STDS*. 2009;23(9):743-7.
65. Garenne ML, Coninx R, Dupuy C. Effects of the civil war in central Mozambique and evaluation of the intervention of the International Committee of the Red Cross. *J Trop Pediatr*. 1997;43(6):318-23.
66. Gaspar M, Leite F, Brumana L, et al. Epidemiology of meningococcal meningitis in Angola, 1994–2000. *Epidemiol Infect*. 2001;127:421-4.
67. Gelaw Y, Abateneh A. Blinding trachoma among refugees: Complicating social disaster. *Asian Pacific Journal of Tropical Biomedicine*. 2015;5(2):124-7.
68. Ghebreyesus TA, Alemayehu T, Bosman A, et al. Community participation in malaria control in Tigray region Ethiopia. *Acta Trop*. 1996;61(2):145-56.
69. Goodson J, Alexander J, Husain F. Measles - horn of Africa, 2010-2011. *MMWR Morb Mortal Wkly Rep*. 2012;61(34):678-84.
70. Goodrich S, Ndege S, Kimaiyo S, et al. Delivery of HIV care during the 2007 post-election crisis in Kenya: a case study analyzing the response of the Academic Model Providing Access to Healthcare (AMPATH) program. *Conflict and Health*. 2013;7:1-12.

71. Gorbacheva O, Mishra AK, Shapovalov D, et al. Prevalence of bacteriologically confirmed pulmonary tuberculosis in the Bhutanese refugees in Nepal. Results of active case finding. *Int J Infect Dis*. 2010;14
72. WHO/GPA. GPA joins emergency efforts in Rwanda. Switzerland; 1994. Contract No.: 4.
73. Graham K, Mohammad N, Rehman H, et al. Comparison of three pyrethroid treatments of top-sheets for malaria control in emergencies: Entomological and user acceptance studies in an afghan refugee camp in pakistan. *Med Vet Entomol*. 2002;16(2):199-206.
74. Graham K, Mohammad N, Rehman H, et al. Insecticide-treated plastic tarpaulins for control of malaria vectors in refugee camps. *Med Vet Entomol*. 2002;16(4):404-8.
75. Habib M, Soofi S, Cousens S, et al. Community engagement and integrated health and polio immunisation campaigns in conflict-affected areas of Pakistan: a cluster randomised controlled trial. *The Lancet*. 2017;5:593-603.
76. Haelterman E, Boelaert M, Suetens C, et al. Impact of a mass vaccination campaign against a meningitis epidemic in a refugee camp. *Trop Med Int Health*. 1996;1(3):385-92.
77. Hampton T. Innovative program offers HIV therapy to internally displaced persons in Uganda. *JAMA: Journal of the American Medical Association*. 2008;300(5):493-.
78. Hamze H, Charchuk R, Jean Paul MK, et al. Lack of household clustering of malaria in a complex humanitarian emergency: implications for active case detection. *Pathogens and Global Health*. 2016;110(6):223-7.
79. Hehenkamp A, Hargreaves S. Tuberculosis treatment in complex emergencies: South Sudan. *Lancet*. 2003;362 Suppl((Hehenkamp, Hargreaves) Medecins Sans Frontieres mission in South, Sudan.):s30-1.
80. Heldal E, et al. Successful management of a national tuberculosis programme under conditions of war. *Int J Tuberc Lung Dis*. 1997;1(1):16-24.
81. Hemhongsa P, Tasaneeyapan T, Swaddiwudhipong W, et al. TB, HIV-associated TB and multidrug-resistant TB on Thailand's border with Myanmar, 2006-2007. *Trop Med Int Health*. 2008;13(10):1288-96.
82. Hindiyeh MY, Aboudy Y, Wohoush M, et al. Characterization of large mumps outbreak among vaccinated palestinian refugees. *J Clin Microbiol*. 2009;47(3):560-5.
83. Howard N, Durrani N, Sanda S, et al. Clinical trial of extended-dose chloroquine for treatment of resistant falciparum malaria among Afghan refugees in Pakistan. *Malar J*. 2011;10((Durrani, Rowland) HealthNet-TPO, Peshawar, Pakistan):no pagination.
84. Hutagalung R, Paiphun L, Ashley EA, et al. A randomized trial of artemether-lumefantrine versus mefloquine-artesunate for the treatment of uncomplicated multi-drug resistant Plasmodium falciparum on the western border of Thailand. *Malar J*. 2005;4:46.
85. ICRC. Facts and Figures 2016: Cotabato Office. 2016.
86. Iyer AS, Bouhenia M, Rumunu J, et al. Immune Responses to an Oral Cholera Vaccine in Internally Displaced Persons in South Sudan. *Sci Rep*. 2016;6(101563288):35742.
87. Jarallah JS. Tuberculosis among Afghani refugees: Epidemiology, clinical pattern and assessment of default and non-compliance. *Saudi Med J*. 1993;14(3):233-6.
88. Javaloy J, Ferrer C, Vidal MT, et al. Follicular conjunctivitis caused by Chlamydia trachomatis in an infant Saharan population: molecular and clinical diagnosis. *The British journal of ophthalmology*. 2003;87(2):142-6.
89. Kaiser R, Kedamo T, Lane J, et al. HIV, syphilis, herpes simplex virus 2, and behavioral surveillance among conflict-affected populations in Yei and Rumbek, southern Sudan. *AIDS*. 2006;20(6):942-4.
90. Kajeechiwa L, Thwin MM, Shee PW, et al. The acceptability of mass administrations of anti-malarial drugs as part of targeted malaria elimination in villages along the Thai-Myanmar border. *Malar J*. 2016;15(1):494.
91. Kamadjeu R, Mahamud A, Webeck J, et al. Polio outbreak investigation and response in Somalia, 2013. *J Infect Dis*. 2014;210((Bile) Somalia Ministry of Human Development and Public Services, Mogadishu, United States):S181-S6.
92. Kasereka CM, Katsuva JPM, Hawkes M. Malaria case-finding and treatment strategies in an internally displaced persons (IDP) camp in the democratic republic of Congo. *Am J Trop Med Hyg*. 2014;91(5 SUPPL. 1):105.
93. Keus K, Houston S, Melaku Y, et al. Field research in humanitarian medical programmes. Treatment of a cohort of tuberculosis patients using the Manyatta regimen in a conflict zone in South Sudan. *Trans R Soc Trop Med Hyg*. 2003;97(6):614-8.
94. Kiboneka A, Edward JM. Combination antiretroviral therapy in population affected by conflict: outcomes from large cohort in northern Uganda. *BMJ*. 2009;338:201.
95. Kim AA, Malele F, Kaiser R, et al. HIV infection among internally displaced women and women residing in river populations along the congo river, democratic republic of Congo. *AIDS Behav*. 2009;13(5):914-20.
96. Kimani EW, Vulule JM, Kuria IW, et al. Use of insecticide-treated clothes for personal protection against malaria: A community trial. *Malar J*. 2006;5((Kuria) Care International Kenya, GPO, P.O. Box 43864-00100, Nairobi, Kenya):no pagination.
97. Kline D, Dakkak H, Cami A. Vaccination campaign for Kosovar Albanian refugee children--former Yugoslav Republic of Macedonia, April-May, 1999. *MMWR Morbidity and mortality weekly report*. 1999;48(36):799-803.
98. Koop DG, Jackson BM, Nestel G. Results of the expanded program on immunization in the Macedonian refugee camps. *Am J Public Health*. 2001;91(10):1656-9.
99. Koscalova A, M I. Evaluation of two emergency interventions: outbreak of malaria and epidemic of measles, DRC. Geneva, Switzerland: MSF; 2014.
100. Kouadio IK, Koffi AK, Attoh-Toure H, et al. Outbreak of measles and rubella in refugee transit camps. *Epidemiol Infect*. 2009;137(11):1593-601.
101. Krause S, Williams H, Onyango M, et al. Reproductive health services for Syrian refugees in Zaatri Camp and Irbid City, Hashemite Kingdom of Jordan: an evaluation of the Minimum Initial Services Package. *Conflict and Health*. 2015;9:1-10.
102. Kruk ME, Rockers PC, Williams EH, et al. Availability of essential health services in post-conflict Liberia. 2010;88:527–34.
103. Lam E, Al-Tamimi W, Russell SP, et al. Oral cholera vaccine coverage during an outbreak and humanitarian Crisis, Iraq, 2015. *Emerg Infect Dis*. 2017;23(1):38-45.
104. Liddle KF, Elema R, Thi SS, et al. TB treatment in a chronic complex emergency: treatment outcomes and experiences in Somalia. *Trans R Soc Trop Med Hyg*. 2013;107(11):690-8.
105. Legros D, Paquet C, Perea W, et al. Mass vaccination with a two-dose oral cholera vaccine in a refugee camp. *Bull World Health Organ*. 1999;77(10):837-42.
106. Luxemburger C, ter Kuile FO, Nosten F, et al. Single day mefloquine-artesunate falciparum resistant malaria. *Trans R Soc Trop Med Hyg*. 1994;88:213-7.

107. Luxemburger C, Perea WA, Delmas G, et al. Permethrin-impregnated on the Thai-Burmese, bed nets for the prevention border. *Trans R Soc Trop Med Hyg*. 1994;88:155-9.
108. Luxemburger C, et al. Oral artesunate in the treatment of uncomplicated hyperparasitemic falciparum malaria. *Am J Trop Med Hyg*. 1995;53(5):522-5.
109. Luxemburger C, Thwai KL, White NJ, et al. The epidemiology of malaria in a Karen population on the western border of Thailand. *Trans R Soc Trop Med Hyg*. 1996;90(2):105-11.
110. Luxemburger C, et al. Treatment of vivax malaria on the western border of Thailand. *Trans R Soc Trop Med Hyg*. 1999;93(4):433-8.
111. Mahalanabis D, Choudhuri AB, Bagchi NG, et al. Oral fluid therapy of cholera among Bangladesh refugees[1]. *WHO South-East Asia journal of public health*. 2012;1(1):105-12.
112. Makokha FM, Unshur A, Hussein A, et al. Coverage during an immunization campaign providing inactivated and oral polio vaccines in refugee camps and host communities, Kenya-December 2013. *Am J Trop Med Hyg*. 2014;91(5 SUPPL. 1):433.
113. Marfin AA, Moore J, Collins C, et al. Infectious disease surveillance during emergency relief to Bhutanese refugees in Nepal. *J Am Med Assoc*. 1994;272(5):377-81.
114. Matthys F, Male S, Z L. Cholera Outbreak among Rwandan Refugees — Democratic Republic of Congo, April 1997. *CDC*. 1998;47:389-91.
115. Mayaud P. The challenges of sexually transmitted infections control for HIV prevention in refugee settings: Rwandan refugees in Tanzania. *Trans R Soc Trop Med Hyg*. 2001;95:121-4.
116. Mbaeyi C, Ryan MJ, Smith P, et al. Response to a Large Polio Outbreak in a Setting of Conflict - Middle East, 2013-2015. *MMWR Morbidity and mortality weekly report*. 2017;66(8):227-31.
117. McGinn T, Allen K. Improving refugees' reproductive health through literacy in Guinea. *Global Public Health*. 2006;1:229-48.
118. McGready R, et al. Randomized comparison of mefloquine-artesunate versus quinine in the treatment of multidrug-resistant falciparum malaria in pregnancy. *Trans R Soc Trop Med Hyg*. 2000;94(6):689-93.
119. McGready R, Boel M, Rijken MJ, et al. Effect of early detection and treatment on malaria related maternal mortality on the north-western border of thailand 1986-2010. *PLoS One*. 2012;7(7):no pagination.
120. Mendelsohn JB, Schilperoord M, Spiegel P, et al. Is forced migration a barrier to treatment success? Similar HIV treatment outcomes among refugees and a surrounding host community in Kuala Lumpur, Malaysia. *AIDS Behav*. 2014;18(2):323-34.
121. Minetti A, Camelique O, Hsa Thaw K, et al. Tuberculosis treatment in a refugee and migrant population: 20 Years of experience on the Thai-Burmese border. *Int J Tuberc Lung Dis*. 2010;14(12):1589-95.
122. Morrison V. Contraceptive Need Among Cambodian Refugees In Khao Phlu Camp. *Guttmacher Institute*. 2000;26:188-92.
123. Msuya W, Mayaud P, Mkanje R, et al. Taking early action in emergencies to reduce the spread of STDs and HIV. *Africa health*. 1996;18(5):24.
124. Mupere E, Onek P, Babikako HM. Impact of emergency mass immunisations on measles control in displaced populations in Gulu district, Northern Uganda. *East Afr Med J*. 2005;82(8):403-8.
125. Navarro-Colorado C, Mahamud A, Burton A, et al. Measles outbreak response among adolescent and adult Somali refugees displaced by famine in Kenya and Ethiopia, 2011. *J Infect Dis*. 2014;210(12):1863-70.
126. Nnadi C, Damisa E, Esapa L, et al. Continued Endemic Wild Poliovirus Transmission in Security-Compromised Areas - Nigeria, 2016. *MMWR Morbidity and mortality weekly report*. 2017;66(7):190-3.
127. Nosten F, ter Kuile F, Maelankirri L, et al. Malaria during pregnancy in an area of unstable endemicity. *Trans R Soc Trop Med Hyg*. 1991;85(4):424-9.
128. Nosten F, et al. Randomised double-blind placebo-controlled trial of SPf66 malaria vaccine in children in northwestern Thailand. *Lancet*. 1999;348:701-7.
129. Nosten F, McGready R, Simpson JA, et al. Effects of Plasmodium vivax malaria in pregnancy. *Lancet*. 1999;354(9178):546-9.
130. Obol JH, Ononge S, Orach CG. Utilisation of insecticide treated nets among pregnant women in Gulu: A post conflict district in northern Uganda. *Afr Health Sci*. 2013;13(4):962-9.
131. O'Brien Dp MCHCFNPK. Universal access: the benefits and challenges in bringing integrated HIV care to isolated and conflict affected populations in the Republic of Congo. *Confl Health*. 2009;3:1-10.
132. O'Laughlin KN, Kasozi J, Walensky RP, et al. Clinic-based routine voluntary HIV testing in a refugee settlement in Uganda. *J Acquir Immune Defic Syndr*. 2014;67(4):409-13.
133. WHO. Oral cholera vaccine campaign among internally displaced persons in South Sudan. World Health Organization; 2014. Contract No.: 20.
134. Petersen E, Baekeland S, Memish ZA, et al. Infectious disease risk from the Syrian conflict. *Int J Infect Dis*. 2013;17(9):e666-e7.
135. Phares CR, Date K, Travers P, et al. Mass vaccination with a two-dose oral cholera vaccine in a long-standing refugee camp, Thailand. *Vaccine*. 2016;34(1):128-33.
136. Plewes K, Lee T, Kajeechewa L, et al. Low seroprevalence of HIV and syphilis in pregnant women in refugee camps on the Thai-Burma border. *Int J STD AIDS*. 2008;19(12):833-7.
137. WHO. Poliomyelitis outbreak in Somalia and Kenya, 2013. Switzerland; 2013. Contract No.: 33.
138. Porta MI, Lenglet A, De Weerdt S, et al. Feasibility of a preventive mass vaccination campaign with two doses of oral cholera vaccine during a humanitarian emergency in South Sudan. *Trans R Soc Trop Med Hyg*. 2014;108(12):810-5.
139. Porter JDH, Gastellu-Etchegorry M, Navarre I, et al. Measles outbreaks in the Mozambican refugee camps in malawi: The continued need for an effective vaccine. *Int J Epidemiol*. 1990;19(4):1072-7.
140. WHO. Prevention of measles deaths in Darfur, Sudan. Switzerland; 2004. Contract No.: 38.
141. Price R, et al. Artesunate versus artemether in combination with mefloquine for the treatment of multidrug-resistant falciparum malaria. *Trans R Soc Trop Med Hyg*. 1995;89(5):523-7.
142. Price R, et al. Effects of artemisinin derivatives on malaria transmissibility. *Lancet*. 1996;347(9016):1654-8.
143. Price R, et al. Artesunate/mefloquine treatment of multi-drug resistant falciparum malaria. *Trans R Soc Trop Med Hyg*. 1997;91(5):574-7.
144. Price R, et al. Artesunate and mefloquine in the treatment of uncomplicated multidrug-resistant hyperparasitaemic falciparum malaria. *Trans R Soc Trop Med Hyg*. 1998;92(2):207-11.
145. Price R, et al. Artesunate versus artemether for the treatment of recrudescent multidrug-resistant falciparum malaria. *Am J Trop Med Hyg*. 1998;59(6):883-8.

146. WHO. Progress toward poliomyelitis eradication--Democratic Republic of Congo, 1996-1999. United States; 2000. Contract No.: 12.
147. Protopopoff N, et al. Vector control in a malaria epidemic occurring within a complex emergency situation in Burundi: a case study. *Malar J*. 2007;6:93.
148. Raoult D, Ndihokubwayo JB, Tissot-Dupont H, et al. Outbreak of epidemic typhus associated with trench fever in Burundi. *Lancet*. 1998;352(9125):353-8.
149. Reid T, Engelgem IV, Telfer B, et al. Providing HIV care in the aftermath of Kenya's post-election violence Medecins Sans Frontieres' lessons learned January – March 2008. *Conflict and Health*. 2008;2:1-5.
150. Reynolds MG, Emerson GL, Pukuta E, et al. Short report: Detection of human monkeypox in the Republic of the Congo following intensive community education. *Am J Trop Med Hyg*. 2013;88(5):982-5.
151. Richards AK, Banek K, Mullany LC, et al. Cross-border malaria control for internally displaced persons: Observational results from a pilot programme in eastern Burma/Myanmar. *Trop Med Int Health*. 2009;14(5):512-21.
152. Richardson L, Bush A, G A. An Independent Review of UNHCR’s Response to the Somali Refugee Influx in Dollo Ado, Ethiopia, 2011. Geneva, Switzerland: UNHCR; 2013.
153. Roca MG, Charle P, Jimenez S, et al. A new malaria protocol in a Congolese refugee camp in West Tanzania. *Global Public Health*. 2011;6(4):398-406.
154. Rodger AJ, Toole M, Lalnuntluangi B, et al. DOTS-based tuberculosis treatment and control during civil conflict and an HIV epidemic, Churachandpur District, India. *Bull World Health Organ*. 2002;80(6):451-6.
155. Rosenberg JS, D B. Let's talk about sex work in humanitarian settings: piloting a rights-based approach to working with refugee women selling sex in Kampala. *Reproductive Health Matters*. 2017;25:95-102.
156. Rowland M, Hewitt S, Durrani N. Prevalence of malaria in Afghan refugee villages in Pakistan sprayed with lambdacyhalothrin or malathion. *Trans R Soc Trop Med Hyg*. 1994;88(4):378-9.
157. Rowland M, Bouma M, Ducornez D, et al. Pyrethroid-impregnated bed nets for personal protection against malaria for Afghan refugees. *Trans R Soc Trop Med Hyg*. 1996;90(4):357-61.
158. Rowland M, Hewitt S, Durrani N, et al. Sustainability of pyrethroid-impregnated bednets for malaria control in Afghan communities. *Bull World Health Organ*. 1997;75(1):23-9.
159. Rowland M, Durrani N, Hewitt S, et al. Permethrin-treated chaddars and top-sheets: Appropriate technology for protection against malaria in Afghanistan and other complex emergencies. *Trans R Soc Trop Med Hyg*. 1999;93(5):465-72.
160. Rowland M, Durrani N. Randomized controlled trials of 5- and 14-days primaquine therapy against relapses of vivax malaria in an Afghan refugee settlement in Pakistan. *Trans R Soc Trop Med Hyg*. 1999;93(6):641-3.
161. Rowland M, Durrani N, Kenward M, et al. Control of malaria in Pakistan by applying deltamethrin insecticide to cattle: A community-randomised trial. *Lancet*. 2001;357(9271):1837-41.
162. Rowland M, et al. Prevention of malaria in Afghanistan through social marketing of insecticide-treated nets:evaluation of coverage and effectiveness by cross-sectional surveys and passive surveillance. *Trop Med Int Health*. 2002;7(10):813-22.
163. Rowland M, Downey G, Rab A, et al. DEET mosquito repellent provides personal protection against malaria: A household randomized trial in an Afghan refugee camp in Pakistan. *Trop Med Int Health*. 2004;9(3):335-42.
164. Rowley EA, Spiegel PB, Tunze Z, et al. Differences in HIV-related behaviors at Lugufu refugee camp and surrounding host villages, Tanzania. *Conflict and Health*. 2008;2:1-14.
165. Ruckstuhl L, Lengeler C, Moyen JM, et al. Malaria case management by community health workers in the Central African Republic from 2009-2014: Overcoming challenges of access and instability due to conflict. *Malar J*. 2017;16(1):388.
166. Rull M, Masson S, Peyraud N, et al. The new WHO decision-making framework on vaccine use in acute humanitarian emergencies: MSF experience in Minkaman, South Sudan. *Conflict and Health* 2018;12:1-9.
167. Rutta E, Gongo R, Mwansasu A, et al. Prevention of mother-to-child transmission ofHIV in a refugee camp setting in Tanzania. 2008;3:62-76.
168. Rutta E, Kipingili R, Lukonge H, et al. Treatment outcome among Rwandan and Burundian refugees with sputum smear-positive tuberculosis in Ngara, Tanzania. *Int J Tuberc Lung Dis*. 2001;5(7):628-32.
169. Salami O, Buzu A, Nzeme C. High level of adherence to HAART among refugees and internally displaced persons on HAART in western equatorial region of Southern Sudan. *J Int AIDS Soc*. 2010;13.
170. Sallam FA, Albably K, Zvandaziva C, et al. Community engagement through local leadership: Increasing access to nutrition services in a conflict setting in Yemen. Sanaa, Yemen: Nutrition Exchange; 2018.
171. Salse N, T S, Xavier KF, et al. Effectiveness of nutritional supplementation (readyto-use therapeutic food and multi micronutrient) in preventing malnutrition in children 6-59 months with infection (malaria, pneumonia, diarrhoea) in Uganda. Geneva, Switzerland; 2013.
172. Santaniello-Newton A, Hunter PR. Management of an outbreak of meningococcal meningitis in a Sudanese refugee camp in Northern Uganda. *Epidemiol Infect*. 2000;124(1):75-81.
173. Saroufim M, Charafeddine K, Issa G, et al. Ongoing epidemic of cutaneous leishmaniasis among Syrian refugees, Lebanon. *Emerg Infect Dis*. 2014;20(10):1712-5.
174. Seaman J, Mercer AJ, Sondorp HE, et al. Epidemic visceral leishmaniasis in southern Sudan: Treatment of severely debilitated patients under wartime conditions and with limited resources. *Ann Intern Med*. 1996;124(7):664-72.
175. Shamomesh M, Shamanesh M. The prevalence of urogenital Chlamydia trachomatis infection in a refugee community in EI Salvador. *Department ofMedicine, Middlesex Hospital*. 1994;5:381-2.
176. Shuaibu FM, Birukila G, Usman S, et al. Mass immunization with inactivated polio vaccine in conflict zones--Experience from Borno and Yobe States, North-Eastern Nigeria. *J Public Health Policy*. 2016;37(1):36-50.
177. Smithuis FM, van Woensel JB, Nordlander E, et al. Comparison of two mefloquine regimens for treatment of Plasmodium falciparum malaria on the northeastern Thai-Cambodian border. *Antimicrob Agents Chemother*. 1993;37(9):1977-81.
178. Somigliana E, Sabino A, Schrettenbrunner C, et al. A comprehensive and integrated project to improve reproductive health at Oyam district, northern Uganda: Insights from maternal death review at the district hospital. *Arch Gynecol Obstet*. 2011;283(3):645-9.
179. Spencer S, Grant AD, Piola P, et al. Malaria in camps for internally-displaced persons in Uganda: Evaluation of an insecticide-treated bednet distribution programme. *Trans R Soc Trop Med Hyg*. 2004;98(12):719-27.
180. Sukrakanchana-Trikham P, Puechal X, Rigal J, et al. 10-year assessment of treatment outcome among Cambodian refugees with sputum smear-positive tuberculosis in Khao-1-Dang, Thailand. *Tuber Lung Dis*. 1992;73(6):384-7.

181. Swerdlow DL, Malenga G, Begkoyian G, et al. Epidemic cholera among refugees in Malawi, Africa: Treatment and transmission. *Epidemiol Infect*. 1997;118(3):207-14.
182. Tanaka Y, Kunii O, Hatano T, et al. Knowledge, attitude, and practice (KAP) of HIV prevention and HIV infection risks among Congolese refugees in Tanzania. *Health & Place*. 2008;14:434-52.
183. ter Kuile FO, Dolan G, Nosten F, et al. Halofantrine versus mefloquine in treatment of multidrug-resistant falciparum malaria. *Lancet (London, England)*. 1993;341(8852):1044-9.
184. UNICEF. The UNICEF Response to the Crisis in the Central African Republic. New York, NY: UNICEF; 2016.
185. Tomashek KM, Woodruff BA, Gotway CA, et al. Randomized intervention study comparing several regimens for the treatment of moderate anemia among refugee children in kigoma region, Tanzania. *Am J Trop Med Hyg*. 2001;64(3-4):164-71.
186. Toole MJ. Public health impact of Rwandan refugee crisis: What happened in Goma, Zaire, in July, 1994? *Lancet*. 1995;345(8946):339-44.
187. Tshipala D, Cornier N, Gounongbe M, et al. Ensuring continuity of antiretroviral therapy among displaced populations during Ivorian post-election violence, 2011. *J Int AIDS Soc*. 2012;15((Bilguissa) UNHCR Consultant, Public Health and HIV Section, Paris, France):266-7.
188. van Vugt M, et al. Randomized comparison of artemether-benflumetol and artesunate-mefloquine in treatment of multidrug-resistant falciparum malaria. *Antimicrob Agents Chemother*. 1998;42(1):135-9.
189. van Vugt M, et al. Efficacy of six doses of artemether-lumefantrine (benflumetol) in multidrug-resistant Plasmodium falciparum malaria. *Am J Trop Med Hyg*. 1999;60(6):936-42.
190. van Vugt M, et al. Artemether-lumefantrine for the treatment of multidrug-resistant falciparum malaria. *Trans R Soc Trop Med Hyg*. 2000;94(5):545-8.
191. van Vugt M, Leonardi E, Phaipun L, et al. Treatment of uncomplicated multidrug-resistant falciparum malaria with artesunate-atovaquone-proguanil. *Clinical infectious diseases: an official publication of the Infectious Diseases Society of America*. 2002;35(12):1498-504.
192. Vitek CR, Velibekov AS. Epidemic diphtheria in the 1990s: Azerbaijan. *J Infect Dis*. 2000;181(SUPPL. 1):S73-S9.
193. von Roenne A, von Roenne F, Kollie S, et al. Reproductive health services for refugees by refugees: an example from Guinea. *Disasters*. 2010;34:16-29.
194. Witzig R, Ismail A, Sayer A, et al. Portable HECT-CL thermotherapy for L. tropica cutaneous leishmaniasis in Aleppo, Syria during 2014. *Trop Med Int Health*. 2015;20:32.

Appendix D. List of Excluded Publications

Citation	Exclusion Reason
Anonymous. Outbreak of Rift Valley fever Yemen August-October 2000. Wkly Epidemiol Rec. 2000; 75(48):392-.	Duplicate
Anonymous. Aiding refugees in the aftermath of civil war. Country focus: Sudan. AIDS Anal Afr. 1996; 6(5):3.	Wrong study design
Anonymous. AIDS epidemic runs riot in South Africa. AIDS Wkly Plus. 1997(9889385):23-4.	Wrong study design
Anonymous. Angola launches rejuvenated programme. AIDS Anal Afr. 1996; 6(1):15.	Wrong topic
Anonymous. Burundi launches campaign against AIDS. AIDS Wkly Plus. 1999:10.	Wrong study design
Anonymous. Cases of polio detected in Yemen. Euro surveillance: bulletin europeen sur les maladies transmissibles = European communicable disease bulletin. 2005; 10(4):E050428.	Wrong setting
Anonymous. Cerebrospinal meningitis. Releve epidemiologique hebdomadaire / Section d'hygiene du Secretariat de la Societe des Nations = Weekly epidemiological record / Health Section of the Secretariat of the League of Nations. 1992; 67(40):297-8.	Wrong setting
Anonymous. Cholera in 1994. Part I. Releve epidemiologique hebdomadaire / Section d'hygiene du Secretariat de la Societe des Nations = Weekly epidemiological record / Health Section of the Secretariat of the League of Nations. 1995; 70(28):201-8.	Wrong study design
Anonymous. Cholera outbreak among Rwandan refugees--Democratic Republic of Congo April 1997. MMWR Morbidity and mortality weekly report. 1998; 47(19):389-91.	Duplicate
Anonymous. Cholera outbreak--southern Sudan 2007. MMWR Morbidity and mortality weekly report. 2009;58 (13):337-41.	Wrong setting
Anonymous. Cholera vaccines: WHO position paper - August 2017. Releve epidemiologique hebdomadaire. 2017;92(34):477-98.	Wrong study design
Anonymous. Cholera. Outbreak among Rwandan refugees. Releve epidemiologique hebdomadaire / Section d'hygiene du Secretariat de la Societe des Nations = Weekly epidemiological record / Health Section of the Secretariat of the League of Nations. 1994;69(30):221.	Wrong study design
Anonymous. Cholera: unjustified control measures. Releve epidemiologique hebdomadaire / Section d'hygiene du Secretariat de la Societe des Nations = Weekly epidemiological record / Health Section of the Secretariat of the League of Nations. 1994;69(45):337-8.	Wrong study design
Anonymous. Circulating vaccine-derived poliovirus outbreaks in 5 countries 2014-2015. Releve epidemiologique hebdomadaire. 2016;91(6):71-2.	Wrong setting
Anonymous. Communicable disease control in complex emergencies. Releve epidemiologique hebdomadaire / Section d'hygiene du Secretariat de la Societe des Nations = Weekly epidemiological record / Health Section of the Secretariat of the League of Nations. 2000;75(50):409-11.	Wrong study design
Anonymous. Communicable disease hazards facing refugees from Kosovo. Commun Dis Rep CDR Wkly. 1999;9(17):147-50.	Wrong study design
Anonymous. Confronting a calamity. UN chronicle. 1994;31(2):48-53.	Wrong study design
Anonymous. Control of a cerebrospinal meningitis epidemic. Releve epidemiologique hebdomadaire / Section d'hygiene du Secretariat de la Societe des Nations = Weekly epidemiological record / Health Section of the Secretariat of the League of Nations. 1993;68(33):237-8.	Wrong setting
Anonymous. Cryptosporidiosis. Commun Dis Rep CDR Wkly. 1993;3(20):89.	Wrong setting

Anonymous. Dengue and dengue haemorrhagic fever. 1990-1994. Releve epidemiologique hebdomadaire / Section d'hygiene du Secretariat de la Societe des Nations = Weekly epidemiological record / Health Section of the Secretariat of the League of Nations. 1995;70(47):334-5.	Wrong setting
Anonymous. Dengue and dengue haemorrhagic fever. Releve epidemiologique hebdomadaire / Section d'hygiene du Secretariat de la Societe des Nations = Weekly epidemiological record / Health Section of the Secretariat of the League of Nations. 1994;69(36):265-6.	Wrong setting
Anonymous. Dengue hemorrhagic fever in Venezuela. Epidemiol Bull. 1990;11(2):7-9.	Wrong setting
Anonymous. Diphtheria in the former Soviet Union: the epidemic continues. Soz Praventivmed. 1994;39(3):182.	Wrong study design
Anonymous. Diphtheria in Ukraine. Commun Dis Rep CDR Wkly. 1994;4(38):177.	Wrong study design
Anonymous. Emergency measles control activities--Darfur Sudan 2004. MMWR Morbidity and mortality weekly report. 2004;53(38):897-9.	Duplicate
Anonymous. Emerging infectious diseases. Releve epidemiologique hebdomadaire / Section d'hygiene du Secretariat de la Societe des Nations = Weekly epidemiological record / Health Section of the Secretariat of the League of Nations. 1993;68(49):364-7.	Wrong setting
Anonymous. Enhanced medical assessment strategy for Barawan Somali refugees--Kenya 1997. MMWR Morbidity and mortality weekly report. 1998;46(52-53):1250-4.	Duplicate
Anonymous. Enteroviral meningitis outbreak Kosovo July-September 2006. Euro surveillance: bulletin European sur les maladies transmissibles = European communicable disease bulletin. 2006;11(9):E060914.	Wrong setting
Anonymous. Epidemic typhus risk in Rwandan refugee camps. Releve epidemiologique hebdomadaire / Section d'hygiene du Secretariat de la Societe des Nations = Weekly epidemiological record / Health Section of the Secretariat of the League of Nations. 1994;69(34):259.	Wrong study design
Anonymous. ERJ July Podcast: tuberculosis prevention and control in refugees. The European respiratory journal. 2016;48(1):E67.	Wrong study design
Anonymous. Expanded programme on immunization (EPI). Immunization schedules in the WHO eastern Mediterranean region 1995. Releve epidemiologique hebdomadaire / Section d'hygiene du Secretariat de la Societe des Nations = Weekly epidemiological record / Health Section of the Secretariat of the League of Nations. 1996;71(23):173-6.	Wrong study design
Anonymous. Expanded programme on immunization (EPI). Update: diphtheria epidemic in the newly independent states of the former USSR January 1995-March 1996. Releve epidemiologique hebdomadaire / Section d'hygiene du Secretariat de la Societe des Nations = Weekly epidemiological record / Health Section of the Secretariat of the League of Nations. 1996;71(33):245-50.	Wrong setting
Anonymous. Expanded programme on immunization. Diphtheria epidemic. Releve epidemiologique hebdomadaire / Section d'hygiene du Secretariat de la Societe des Nations = Weekly epidemiological record / Health Section of the Secretariat of the League of Nations. 1994;69(34):253-8.	Wrong study design
Anonymous. Expanded programme on immunization. Measles 1994. Releve epidemiologique hebdomadaire / Section d'hygiene du Secretariat de la Societe des Nations = Weekly epidemiological record / Health Section of the Secretariat of the League of Nations. 1995;70(40):284-8.	Wrong setting

Anonymous. Expanded programme on immunization. Measles epidemic 1989-1990. Releve epidemiologique hebdomadaire / Section d'hygiene du Secretariat de la Societe des Nations = Weekly epidemiological record / Health Section of the Secretariat of the League of Nations. 1992;67(8):50-4.	Wrong setting
Anonymous. Expanded programme on immunization. Measles outbreak in N'Djamena. Releve epidemiologique hebdomadaire / Section d'hygiene du Secretariat de la Societe des Nations = Weekly epidemiological record / Health Section of the Secretariat of the League of Nations. 1995;70(5):31-5.	Wrong setting
Anonymous. Expanded programme on immunization. Measles outbreak Kampala. Releve epidemiologique hebdomadaire / Section d'hygiene du Secretariat de la Societe des Nations = Weekly epidemiological record / Health Section of the Secretariat of the League of Nations. 1991;66(49):364-7.	Wrong setting
Anonymous. Expanded programme on immunization. Measles outbreak. Releve epidemiologique hebdomadaire / Section d'hygiene du Secretariat de la Societe des Nations = Weekly epidemiological record / Health Section of the Secretariat of the League of Nations. 1990;65(49):379-81.	Wrong setting
Anonymous. Expanded programme on immunization. Measles outbreak. Releve epidemiologique hebdomadaire / Section d'hygiene du Secretariat de la Societe des Nations = Weekly epidemiological record / Health Section of the Secretariat of the League of Nations. 1993;68(7):45-7.	Wrong setting
Anonymous. Expanded programme on immunization. Outbreak of diphtheria update. Releve epidemiologique hebdomadaire / Section d'hygiene du Secretariat de la Societe des Nations = Weekly epidemiological record / Health Section of the Secretariat of the League of Nations. 1993; 68(19):134-40.	Wrong setting
Anonymous. Expanded programme on immunization. Outbreak of diphtheria. Releve epidemiologique hebdomadaire / Section d'hygiene du Secretariat de la Societe des Nations = Weekly epidemiological record / Health Section of the Secretariat of the League of Nations. 1991; 66(25):181-5.	Wrong setting
Anonymous. Expanded programme on immunization. Poliomyelitis outbreak 1992. Releve epidemiologique hebdomadaire / Section d'hygiene du Secretariat de la Societe des Nations = Weekly epidemiological record / Health Section of the Secretariat of the League of Nations. 1993;68(41):297-300.	Wrong setting
Anonymous. Expanded programme on immunization. Poliomyelitis outbreak. Releve epidemiologique hebdomadaire / Section d'hygiene du Secretariat de la Societe des Nations = Weekly epidemiological record / Health Section of the Secretariat of the League of Nations. 1992;67(46):341-4.	Wrong setting
Anonymous. Expanded programme on immunization. Releve epidemiologique hebdomadaire / Section d'hygiene du Secretariat de la Societe des Nations = Weekly epidemiological record / Health Section of the Secretariat of the League of Nations. 1993;68(36):261-4.	Wrong setting
Anonymous. Expanded programme on immunization. Rubella outbreak. Releve epidemiologique hebdomadaire / Section d'hygiene du Secretariat de la Societe des Nations = Weekly epidemiological record / Health Section of the Secretariat of the League of Nations. 1994;69(45):333-7.	Wrong setting
Anonymous. For 10 busy weeks action -- but little TB: Fort Dix is screening base for Kosovar refugees. TB Monitor. 2000;7(2):17-9.	Wrong study design

Anonymous. From the Centers for Disease Control and Prevention. Update: influenza activity 1992. JAMA : the journal of the American Medical Association. 1992;268(19):2632-4.	Wrong study design
Anonymous. Global. Refugees women must be included in national HIV/AIDS treatment. AIDS Policy Law. 2007;22(6):1-4.	Wrong study design
Anonymous. Health status of displaced persons following Civil War--Burundi December 1993-January 1994. MMWR Morbidity and mortality weekly report. 1994;43(38):701-3.	Wrong study design
Anonymous. Hepatitis e Chad. Wkly Epidemiol Rec. 2004;79(35):313-.	Wrong setting
Anonymous. HIV and refugees. AIDS Action. 1999(44):7.	Wrong study design
Anonymous. HIV risk to asylum seekers' babies. RCM Midwives Journal. 2002;5(4):114-.	Wrong setting
Anonymous. HIV treatment regimens can benefit refugees. AIDS Policy Law. 2006;21(17):1.	Wrong study design
Anonymous. HIV worldwide shows no slowing in infections deaths: more sub-Saharan African women are infected with HIV than men. AIDS Alert. 2000;15(2):1-2.	Wrong study design
Anonymous. Human monkeypox in Kasai Oriental Zaire (1996-1997). Releve epidemiologique hebdomadaire / Section d'hygiene du Secretariat de la Societe des Nations = Weekly epidemiological record / Health Section of the Secretariat of the League of Nations. 1997;72(15):101-4.	Wrong study design
Anonymous. Infectious diseases kill over 17 million people a year: WHO warns of global crisis. Indian Pediatr. 1996;33(7):617-23.	Wrong study design
Anonymous. Influenza. Commun Dis Rep CDR Wkly. 1992;2(7):30.	Wrong setting
Anonymous. Influenza. Releve epidemiologique hebdomadaire / Section d'hygiene du Secretariat de la Societe des Nations = Weekly epidemiological record / Health Section of the Secretariat of the League of Nations. 1995;70(49):348-50.	Wrong study design
Anonymous. International notes: Surveillance of health status of Bhutanese refugees - Nepal 1992. J Am Med Assoc. 1993;269(7):846.	Wrong study design
Anonymous. Iraq war is public health disaster report finds. Clin Infect Dis. 2005;40(2):iii-iv.	Wrong study design
Anonymous. Listeriosis update. Commun Dis Rep CDR Wkly. 1993;3(13):57.	Wrong setting
Anonymous. Lyme disease 1991-1992. Releve epidemiologique hebdomadaire / Section d'hygiene du Secretariat de la Societe des Nations = Weekly epidemiological record / Health Section of the Secretariat of the League of Nations. 1993;68(37):273-4.	Wrong setting
Anonymous. Malaria. Localized outbreak. Releve epidemiologique hebdomadaire / Section d'hygiene du Secretariat de la Societe des Nations = Weekly epidemiological record / Health Section of the Secretariat of the League of Nations. 1993;68(39):285-6.	Wrong setting
Anonymous. Mass immunization campaign launched to protect 15 million children from polio as outbreak in Nigeria spreads across West Africa. Releve epidemiologique hebdomadaire / Section d'hygiene du Secretariat de la Societe des Nations = Weekly epidemiological record / Health Section of the Secretariat of the League of Nations. 2003;78(44):385-7.	Wrong study design
Anonymous. Mass vaccination campaigns to control the 1991-1992 poliomyelitis outbreak. Releve epidemiologique hebdomadaire / Section d'hygiene du Secretariat de la Societe des Nations = Weekly epidemiological record / Health Section of the Secretariat of the League of Nations. 1993;68(30):222-3.	Wrong setting
Anonymous. Measles epidemic spreads straining nursing services. The American journal of nursing. 1990;90(7):67-70.	Wrong setting

Anonymous. Measles in 1992. Releve epidemiologique hebdomadaire / Section d'hygiene du Secretariat de la Societe des Nations = Weekly epidemiological record / Health Section of the Secretariat of the League of Nations. 1993;68(33):241-3.	Wrong setting
Anonymous. Measles in the Horn of Africa 2010-2011. Releve epidemiologique hebdomadaire / Section d'hygiene du Secretariat de la Societe des Nations = Weekly epidemiological record / Health Section of the Secretariat of the League of Nations. 2012;87(35):329-36.	Duplicate
Anonymous. Measles outbreak in Bulgaria. Releve epidemiologique hebdomadaire / Section d'hygiene du Secretariat de la Societe des Nations = Weekly epidemiological record / Health Section of the Secretariat of the League of Nations. 1992;67(12):84-5.	Wrong setting
Anonymous. Measles vaccines: WHO position paper -- April 2017. Wkly Epidemiol Rec. 2017;92(17):205-27.	Wrong study design
Anonymous. Meningococcal disease Ethiopia (update). Releve epidemiologique hebdomadaire / Section d'hygiene du Secretariat de la Societe des Nations = Weekly epidemiological record / Health Section of the Secretariat of the League of Nations. 2000;75(15):117-8.	Wrong setting
Anonymous. Migration and AIDS. International Migration. 1998;36(4):445-68.	Wrong study design
Anonymous. Mobile populations and AIDS: moving in the right direction. AIDS health promotion exchange / World Health Organization Global Programme on AIDS Health Promotion Unit. 1993(1):13-5.	Wrong study design
Anonymous. Monkeypox: virus outbreak in Africa biggest ever. Health Letter on the CDC. 1997:7-.	Wrong study design
Anonymous. Mortality among newly arrived Mozambican refugees--Zimbabwe and Malawi 1992. MMWR Morbidity and mortality weekly report. 1993;42(24):468-77.	Wrong study design
Anonymous. Mortality during a famine--Gode district Ethiopia July 2000. MMWR Morbidity and mortality weekly report. 2001;50(15):285-8.	Wrong study design
Anonymous. MSI develops refugee initiative. The First people. 1996(101085871):2.	Wrong topic
Anonymous. NGO action on refugees and displaced people. AIDS Anal Afr. 1998;8(3):9.	Wrong topic
Anonymous. Number of cases in Yemen polio outbreak increases dramatically. Euro surveillance : bulletin europeen sur les maladies transmissibles = European communicable disease bulletin. 2005;10(6):E050602.	Wrong setting
Anonymous. Nutritional and health assessment of Mozambican refugees in two districts of Malawi 1988. MMWR Morbidity and mortality weekly report. 1988;37(42):641-3.	Wrong setting
Anonymous. Ongoing outbreak of meningococcal disease in India. Euro surveillance : bulletin europeen sur les maladies transmissibles = European communicable disease bulletin. 2005;10(6):E050602.	Wrong study design
Anonymous. Oral cholera vaccine campaign among internally displaced persons in South Sudan. Releve epidemiologique hebdomadaire / Section d'hygiene du Secretariat de la Societe des Nations = Weekly epidemiological record / Health Section of the Secretariat of the League of Nations. 2014;89(20):214-20.	Duplicate
Anonymous. Outbreak news. Avian influenza Indonesia -- update. Wkly Epidemiol Rec. 2006;81(37):349-.	Wrong study design
Anonymous. Outbreak news. Avian influenza Iraq. Releve epidemiologique hebdomadaire / Section d'hygiene du Secretariat de la Societe des Nations = Weekly epidemiological record / Health Section of the Secretariat of the League of Nations. 2006;81(5):42.	Wrong study design

Anonymous. Outbreak news. Avian influenza Iraq--update. Releve epidemiologique hebdomadaire / Section d'hygiene du Secretariat de la Societe des Nations = Weekly epidemiological record / Health Section of the Secretariat of the League of Nations. 2006;81(6):50-1.	Wrong study design
Anonymous. Outbreak news. Confirmed international spread of wild poliovirus from Pakistan. Releve epidemiologique hebdomadaire / Section d'hygiene du Secretariat de la Societe des Nations = Weekly epidemiological record / Health Section of the Secretariat of the League of Nations. 2011;86(40):437-8.	Wrong setting
Anonymous. Outbreak news. Poliomyelitis Chad. Releve epidemiologique hebdomadaire / Section d'hygiene du Secretariat de la Societe des Nations = Weekly epidemiological record / Health Section of the Secretariat of the League of Nations. 2007;82(7):50.	Wrong setting
Anonymous. Outbreak news. Poliomyelitis Ethiopia and Somalia. Releve epidemiologique hebdomadaire / Section d'hygiene du Secretariat de la Societe des Nations = Weekly epidemiological record / Health Section of the Secretariat of the League of Nations. 2006;81(37):350.	Wrong study design
Anonymous. Outbreak news. Poliomyelitis Kenya. Releve epidemiologique hebdomadaire / Section d'hygiene du Secretariat de la Societe des Nations = Weekly epidemiological record / Health Section of the Secretariat of the League of Nations. 2006;81(43):410.	Wrong study design
Anonymous. Outbreak news. Poliomyelitis Niger. Releve epidemiologique hebdomadaire / Section d'hygiene du Secretariat de la Societe des Nations = Weekly epidemiological record / Health Section of the Secretariat of the League of Nations. 2013;88(7):73-4.	Wrong study design
Anonymous. Outbreak news. Poliomyelitis Somalia. Releve epidemiologique hebdomadaire / Section d'hygiene du Secretariat de la Societe des Nations = Weekly epidemiological record / Health Section of the Secretariat of the League of Nations. 2006;81(13):120.	Wrong study design
Anonymous. Outbreak news. Wild poliovirus in the Horn of Africa. Releve epidemiologique hebdomadaire / Section d'hygiene du Secretariat de la Societe des Nations = Weekly epidemiological record / Health Section of the Secretariat of the League of Nations. 2013;88(40):429-30.	Wrong study design
Anonymous. Outbreak news. Yellow fever Cote d'Ivoire. Releve epidemiologique hebdomadaire / Section d'hygiene du Secretariat de la Societe des Nations = Weekly epidemiological record / Health Section of the Secretariat of the League of Nations. 2006;81(43):410.	Wrong study design
Anonymous. Outbreak news. Yellow fever Sierra Leone. Releve epidemiologique hebdomadaire / Section d'hygiene du Secretariat de la Societe des Nations = Weekly epidemiological record / Health Section of the Secretariat of the League of Nations. 2011;86(12):101-2.	Wrong patient populati
Anonymous. Outbreak news.Outbreak of poliomyelitis Republic of the Congo September 2010-February 2011. Releve epidemiologique hebdomadaire / Section d'hygiene du Secretariat de la Societe des Nations = Weekly epidemiological record / Health Section of the Secretariat of the League of Nations. 2011;86(15):141-2.	Wrong setting
Anonymous. Outbreak of Ebola haemorrhagic fever in Gabon. Commun Dis Rep CDR Wkly. 1996;6(9):75-8.	Wrong study design
Anonymous. Outbreak of Ebola haemorrhagic fever Uganda August 2000-January 2001. Releve epidemiologique hebdomadaire / Section d'hygiene du Secretariat de la Societe des Nations = Weekly epidemiological record / Health Section of the Secretariat of the League of Nations. 2001;76(6):41-6.	Wrong setting

Anonymous. Outbreak of measles--Venezuela and Colombia 2001-2002. MMWR Morbidity and mortality weekly report. 2002;51(34):757-60.	Wrong setting
Anonymous. Outbreak of meningococcal meningitis. Releve epidemiologique hebdomadaire / Section d'hygiene du Secretariat de la Societe des Nations = Weekly epidemiological record / Health Section of the Secretariat of the League of Nations. 1994;69(5):35-6.	Wrong setting
Anonymous. Outbreak of poliomyelitis--Angola 1999. MMWR Morbidity and mortality weekly report. 1999;48(16):327-9.	Duplicate
Anonymous. Outbreak of Rift Valley fever Yemen August-October 2000. Releve epidemiologique hebdomadaire. 2000;75(48):392-5.	Wrong setting
Anonymous. Outbreak of Rift Valley fever Yemen August-October 2000. Wkly Epidemiol Rec. 2000;75(48):392-.	Duplicate
Anonymous. Outbreak(s) of Ebola hemorrhagic fever Congo and Gabon October 2001 to July 2002. Canada communicable disease report = Releve des maladies transmissibles au Canada. 2003;29(15):129-33.	Wrong setting
Anonymous. Outbreaks of measles in communication with low vaccine coverage. Commun Dis Rep CDR Wkly. 2000;10(4):29.	Wrong setting
Anonymous. Overseas screening for tuberculosis in U.S.-bound immigrants and refugees (New England Journal of Medicine (2009) 360 (2406-2415)). N Engl J Med. 2009;361(4):431.	Wrong study design
Anonymous. People on the move. AIDS Action. 1999(44):1.	Wrong study design
Anonymous. Poliomyelitis outbreak spreads across Yemen; case confirmed in Indonesia. Releve epidemiologique hebdomadaire / Section d'hygiene du Secretariat de la Societe des Nations = Weekly epidemiological record / Health Section of the Secretariat of the League of Nations. 2005;80(18):157-8.	Wrong study design
Anonymous. Progress toward poliomyelitis eradication--Afghanistan 1994-1999. MMWR Morbidity and mortality weekly report. 1999;48(37):825-8.	Duplicate
Anonymous. Progress toward poliomyelitis eradication--Nigeria January 2007-August 12 2008. MMWR Morbidity and mortality weekly report. 2008;57(34):942-6.	Duplicate
Anonymous. Progress towards polio eradication Nigeria 1996-1998. Releve epidemiologique hebdomadaire / Section d'hygiene du Secretariat de la Societe des Nations = Weekly epidemiological record / Health Section of the Secretariat of the League of Nations. 1999;74(16):121-4.	Wrong setting
Anonymous. Progress towards poliomyelitis eradication Afghanistan 1994-1999. Releve epidemiologique hebdomadaire / Section d'hygiene du Secretariat de la Societe des Nations = Weekly epidemiological record / Health Section of the Secretariat of the League of Nations. 1999;74(38):316-20.	Duplicate
Anonymous. Public health impact of Rwandan refugee crisis: what happened in Goma Zaire in July 1994? Goma Epidemiology Group. Lancet (London England). 1995;345(8946):339-44.	Wrong study design
Anonymous. Quarterly communicable disease review January to March 1992. From the PHLS Communicable Disease Surveillance Centre. J Public Health Med. 1992;14(3):328-38.	Wrong setting
Anonymous. Reducing the number of disaster refugees. The Lancet. 2014;384(9949):1160.	Wrong topic
Anonymous. Re-emergence of Bolivian hemorrhagic fever. Epidemiol Bull. 1994;15(4):4-5.	Wrong study design
Anonymous. Refugee care costs. New Scientist. 2014;222(2971):7-.	Wrong study design

Meteke S, *et al. BMJ Global Health* 2020; 5:e001967. doi: 10.1136/bmjgh-2019-001967

Anonymous. Refugees women must be included in national HIV/AIDS treatment. AIDS Policy Law. 2007;22(6):1-4.	Wrong study design
Anonymous. Rehabilitating afghanistan. CMAJ. 2002;166(3):293-5.	Wrong study design
Anonymous. Reintroduction of malaria into malaria-free areas. Commun Dis Rep CDR Wkly. 1993;3(40):181.	Wrong study design
Anonymous. Setting up an early warning system for epidemic-prone diseases in the Darfur humanitarian crisis. Releve epidemiologique hebdomadaire / Section d'hygiene du Secretariat de la Societe des Nations = Weekly epidemiological record / Health Section of the Secretariat of the League of Nations. 2004;79(27):246-7.	Wrong study design
Anonymous. Sexual violence against women is a weapon of war. Special feature -- mothers as refugees. Safe motherhood. 1997(23):8.	Wrong topic
Anonymous. Shigellosis. Shigella dysenteriae type 1. Releve epidemiologique hebdomadaire / Section d'hygiene du Secretariat de la Societe des Nations = Weekly epidemiological record / Health Section of the Secretariat of the League of Nations. 1991;66(36):270-1.	Wrong setting
Anonymous. Status of public health - Bosnia and Herzegovina August-September 1993. J Am Med Assoc. 1994;271(12):898-9.	Wrong study design
Anonymous. Surveillance for cholera--Cochabamba Department Bolivia January-June 1992. MMWR Morbidity and mortality weekly report. 1993;42(33):636-9.	Wrong setting
Anonymous. Task force for diphtheria in eastern Europe. Commun Dis Rep CDR Wkly. 1994;4(11):47.	Wrong study design
Anonymous. TDH says watch for Listeria infection. Tex Med. 1991;87(6):22-3.	Wrong setting
Anonymous. The Central African Republic - Still waiting for action. The Lancet. 2013;382(9904):1536.	Wrong study design
Anonymous. The disease problems of Kosovan refugees in Albania. Commun Dis Rep CDR Wkly. 1999;9(18):155.	Wrong study design
Anonymous. The Ebola fever epidemic officially declared over in Zaire. Canada communicable disease report = Releve des maladies transmissibles au Canada. 1995;21(18):164-7.	Wrong study design
Anonymous. The Manipur muddle. Addiction (Abingdon England). 1994;89(7):885.	Wrong topic
Anonymous. The war on Syrian civilians. The Lancet. 2014;383(9915):383.	Wrong study design
Anonymous. Tuberculosis among Indochinese refugees--an update. MMWR Morbidity and mortality weekly report. 1981;30(48):603-6.	Wrong setting
Anonymous. Tuberculosis: Refugees initially become infected while fleeing. Pharm Ztg. 2016;161(12):no pagination.	Non-English
Anonymous. U.S.-incurred costs of wild poliovirus infections in a camp with U.S.-bound refugees--Kenya 2006. MMWR Morbidity and mortality weekly report. 2008;57(9):232-5.	Wrong setting
Anonymous. U.N. details refugees' rights to antiretrovirals. AIDS Policy Law. 2007;22(6):4-.	Wrong study design
Anonymous. Vaccination campaign for Kosovar Albanian refugee children--former Yugoslav Republic of Macedonia April-May 1999. MMWR Morbidity and mortality weekly report. 1999;48(36):799-803.	Duplicate
Anonymous. Vaccination services in postwar Iraq May 2003. MMWR Morbidity and mortality weekly report. 2003;52(31):734-5.	Wrong topic
Anonymous. Vibrio cholerae O 139 and epidemic cholera. Commun Dis Rep CDR Wkly. 1993;3(38):173.	Wrong setting
Anonymous. Violence. Reproductive Health Matters. 2012;20(40):218-9.	Anonymous. Violence. Reproductive Health

	Matters. 2012;20(40):2[illegible] 9.
Anonymous. Violence against women in war: rape AIDS sex slavery. International. AIDS Wkly Plus. 1996(9889385):13-4.	Wrong topic
Anonymous. War oppression refugee camps fuel spread of HIV. Migration and HIV. Bridge (Washington DC : 1992). 1998(5):4-5.	Wrong topic
Anonymous. WHO and UN: AIDS not losing momentum. Public Health Rep. 2000;115(1):7-.	Wrong study design
Anonymous. World Refugee Day: Caring for the forcibly displaced. The Lancet. 2014;383(9935):2100.	Wrong study design
Anonymous. Yellow fever in 1989 and 1990. Releve epidemiologique hebdomadaire / Section d'hygiene du Secretariat de la Societe des Nations = Weekly epidemiological record / Health Section of the Secretariat of the League of Nations. 1992;67(33):245-51.	Wrong setting
Anonymous. Yellow fever in the WHO African and American Regions 2010. Releve epidemiologique hebdomadaire / Section d'hygiene du Secretariat de la Societe des Nations = Weekly epidemiological record / Health Section of the Secretariat of the League of Nations. 2011;86(34):370-6.	Wrong setting
Anonymous. Yellow fever. Epidemic in Cameroon 1990. Releve epidemiologique hebdomadaire / Section d'hygiene du Secretariat de la Societe des Nations = Weekly epidemiological record / Health Section of the Secretariat of the League of Nations. 1991;66(11):76-7.	Wrong setting
Anonymous. Yellow fever. Releve epidemiologique hebdomadaire / Section d'hygiene du Secretariat de la Societe des Nations = Weekly epidemiological record / Health Section of the Secretariat of the League of Nations. 1993;68(22):159-60.	Wrong setting
Anonymous. [Cholera in Goma July 1994. Bioforce]. Rev Epidemiol Sante Publique. 1996;44(4):358-63.	Non-English
Anonymous. [Not Available]. Nursing standard (Royal College of Nursing (Great Britain) : 1987). 1994;9(11):9.	Wrong setting
Anonymous. 250.000 HIV persons in Cameroon 78.000 in N.W. province. AIDS Illus. 1996;2(1):1-11.	Wrong setting
Aaby P Jensen H Garly ML et al. Routine vaccinations and child survival in a war situation with high mortality: Effect of gender. Vaccine. 2002;21(1-2):15-20.	Wrong study design
Aaby P Martins C Bale C et al. Assessing measles vaccination coverage by maternal recall in Guinea-Bissau [14]. Lancet. 1998;352(9135):1229.	Wrong study design
Aavitsland P. Infectious diseases in refugees from Kosovo. Tidsskr Nor Laegeforen. 1999;119(15):2153.	Non-English
Abbara A Al-Harbat N Karah N et al. Antimicrobial drug resistance among refugees from Syria Jordan. Emerg Infect Dis. 2017;23(5):885-6.	Wrong study design
Abbott P Sapsford R Rwirahira J. Rwanda's potential to achieve the millennium development goals for health. World Medical and Health Policy. 2015;7(2):101-20.	Wrong topic
Abdalla E Ekanem E Said D et al. The need for a comprehensive response to HIV/ AIDS in north-western Somalia: evidence from a seroprevalence survey. Eastern Mediterranean health journal = La revue de sante de la Mediterranee orientale = al-Majallah al-sihhiyah li-sharq al-mutawassit. 2010;16(2):141-5.	Wrong study design
Abdella YE Ashghar H Yu J et al. Status of blood safety and availability in the who Eastern Mediterranean Region. Vox Sang. 2016;111((Smit Sibinga) IQM Consulting Zuidhorn Netherlands):7.	Wrong topic

Abdelmoneium AOA. Policy and practice: non-governmental organisations and the health delivery system for displaced children in Khartoum Sudan. Child Abuse Review. 2010;19(3):203-17.	Wrong study design
Abdool-Karim Q Abouzahr C Dehne K et al. HIV and maternal mortality: turning the tide. Lancet. 2010;375 North American Edition(9730):1948-9.	Wrong study design
Abdulrazzaq HA Salwa S Sulaiman SAS et al. Measles outbreak and its contributing factors in an Iraq governorate Diyala. HealthMED. 2012;6(1):69-73.	Wrong study design
Abdur Rab M Freeman TW Rahim S et al. High altitude epidemic malaria in Bamian province central Afghanistan. Eastern Mediterranean Health Journal. 2003;9(3):232-9.	Wrong study design
Abera B Bezabih B Dessie A. Antimicrobial suceptibility of V. cholerae in north west Ethiopia. Ethiop Med J. 2010;48(1):23-8.	Wrong study design
Abera WK. Outbreak investigation of suspected hepatities E among South Sudan refugees Gambella regional state Ethiopia July 2014. Int J Infect Dis. 2016;45((Abera) Ethiopian Public Health Institute Addis Ababa Ethiopia):428.	Wrong study design
Abidi SH Ali F Shah F et al. Burden of communicable disease among the native and repatriating Afghans. PLoS Pathog. 2012;8(10):e1002926.	Wrong study design
Abouteir A El Yaagoubi F Bioh-Johnson I et al. Water access and attendance for diarrhea in primary health care centers Gaza strip. Trans R Soc Trop Med Hyg. 2011;105(10):555-60.	Wrong study design
Abu Mourad TA. Palestinian refugee conditions associated with intestinal parasites and diarrhoea: Nuseirat refugee camp as a case study. Public Health. 2004;118(2):131-42.	Wrong study design
Accorsi S Fabiani M Nattabi B et al. The disease profile of poverty: Morbidity and mortality in northern Uganda in the context of war population displacement and HIV/AIDS. Trans R Soc Trop Med Hyg. 2005;99(3):226-33.	Wrong study design
Acosta CD Kaluski DN Dara M. Conflict and drug-resistant tuberculosis in Ukraine. The Lancet. 2014;384(9953):1500-1.	Wrong study design
Adam AA Karsany MS Adam I. Manifestations of severe Rift Valley fever in Sudan. International journal of infectious diseases : IJID : official publication of the International Society for Infectious Diseases. 2010;14(2):e179-80.	Wrong study design
Adams K Assefi N. Primary care refugee medicine: General principles in the postimmigration care of Somali women. Prim Care Update Ob Gyns. 2002;9(6):210-7.	Wrong study design
Adams KM Gardiner LD Assefi N. Healthcare challenges from the developing world: Post-immigration refugee medicine. Br Med J. 2004;328(7455):1548-52.	Wrong study design
Adhikari BR Shakya G Shrestha S et al. Vibrio cholerae O1 Ogawa a major aetiological agent of diarrhoeal disease outbreak in Western Nepal. Clin Microbiol Infect. 2011;17((Adhikari Shakya	Wrong study design
Adhikari BR Shakya G Upadhyay BP et al. Outbreak of pandemic influenza A/H1N1 2009 in Nepal. Virol J. 2011;8(101231645):133.	Wrong study design
Adja AM Yobo MC Assi SB. Characterization of malaria transmission during military crisis in urban area of Bouake Central Cote d'Ivoire. Int J Infect Dis. 2014;21((Assi) Institut Pierre Richet Abidjan Cote D'Ivoire):103.	Wrong study design
Admasu K Haile-Mariam A Bailey P. Indicators for availability utilization and quality of emergency obstetric care in Ethiopia 2008. International journal of gynaecology and obstetrics: the official organ of the International Federation of Gynaecology and Obstetrics. 2011;115(1):101-5.	Wrong topic

Afghan AK Kassi M Kasi PM et al. Clinical manifestations and distribution of cutaneous leishmaniasis in Pakistan. J Trop Med. 2011((Afghan Kassi Kasi	Wrong study design
Agbaje FO Folayan OA Ifionu NI. Sexual attitude condom use and access among young refugees in oru refugee camp Nigeria. Sex Transm Infect. 2013;89((Agbaje Folayan Ifionu) OROL Youth Empowerment Initiative	Wrong topic
Agha Rodina AI Teoderescu I. Prevalence of intestinal parasites in three localities in Gaza Governorates - Palestine. Archives of Public Health. 2002;60(6):363-70.	Wrong study design
Agoti CN Mayieka LM Otieno JR et al. Examining strain diversity and phylogeography in relation to an unusual epidemic pattern of respiratory syncytial virus (RSV) in a long-term refugee camp in Kenya. BMC Infect Dis. 2014;14(1):no pagination.	Wrong study design
Agoti CN Mwihuri AG Sande CJ et al. Genetic relatedness of infecting and reinfecting respiratory syncytial virus strains identified in a birth cohort from rural Kenya. J Infect Dis. 2012;206(10):1532-41.	Wrong study design
Agua-Agum J Ariyarajah A Blake IM et al. Ebola virus disease among children in West Africa. Waltham Massachusetts: New England Journal of Medicine; 2015. p. 1274-7.	Wrong study design
Ahlawat S Kumar R Roy P et al. Meningococcal meningitis outbreak control strategies. The Journal of communicable diseases. 2000;32(4):264-74.	Wrong setting
Ahmad K. Fears that Afghan exodus threatens polio eradication. Lancet. 2001;358(9288):1161.	Wrong study design
Ahmad K. Measles epidemic sweeps through Afghanistan. Lancet. 2000;355(9213):1439.	Wrong study design
Ahmad K. Pakistan struggles to eradicate polio. The Lancet infectious diseases. 2007;7(4):247.	Wrong study design
Ahmad K. Poliomyelitis spreads in west and central Africa. Lancet Neurol. 2004;3(3):136.	Wrong study design
Ahmad K. Stop TB partnership to focus on Afghanistan and Pakistan. Lancet. 2001;358(9291):1434.	Wrong study design
Ahmad K. War and gerbils compound Afghan leishmaniasis epidemic. The Lancet infectious diseases. 2002;2(5):268.	Wrong study design
Ahmad W Ijaz B Javed F-T et al. HCV genotype distribution and possible transmission risks in Lahore Pakistan. World J Gastroenterol. 2010;16(34):4321-8.	Wrong study design
Ahmadzai TK Maburutse Z Miller L et al. Protecting public health in Yemen. The Lancet. 2016;388(10061):2739.	Wrong study design
Ahmed BH Giovagnoli MR Mahad H et al. Burden of HIV/AIDS infection before and during the civil war in Somalia. Eastern Mediterranean Health Journal. 2010;16(8):907-9.	Wrong study design
Ahmed HM Coulter JBS Nakagomi O et al. Molecular characterization of rotavirus gastroenteritis strains Iraqi Kurdistan. Emerg Infect Dis. 2006;12(5):824-6.	Wrong study design
Ahmed JA Katz MA Auko E et al. Epidemiology of respiratory viral infections in two long-term refugee camps in Kenya 2007-2010. BMC Infect Dis. 2012:7.	Wrong study design
Ahmed JA Katz MA Auko E et al. Epidemiology of respiratory viral infections in two long-term refugee camps in Kenya 2007-2010. BMC Infect Dis. 2012;12(1):7-.	Duplicate
Ahmed JA Moturi E Spiegel P et al. Hepatitis E outbreak Dadaab refugee camp Kenya	Wrong study design
Ahmed MO Daw MA. Mapping the travel route of African refugees who traverse Libya to determine public health implications for Libya and the North-African region. Travel Med Infect Dis. 2016;14(2):162-4.	Wrong study design
Ahmed SS Soghaier MA Mohammed S et al. Concomitant outbreaks of yellow fever and hepatitis E virus in Darfur States Sudan 2012. Journal of Infection in Developing Countries. 2016;10(1):24-9.	Wrong study design

Ahmed W Ahmad M Rafatullah et al. Pervasiveness of intestinal protozoan and worm incursion in IDP's (North waziristan agency KPK-Pakistan) children of 6-16 years. J Pak Med Assoc. 2015;65(9):943-5.	Wrong study design
Akano Y Ando H Tanaka A et al. Oral polio vaccines have not yet covered West Africa: Survey of immunization coverage conducted in Niger. Jpn J Infect Dis. 2004;57(3):121-3.	Wrong study design
Akatcherian C. Pediatrics in countries in war. Arch Pediatr. 2005;12(6):869-70.	Non-English
Akbarzada S Mackey TK. The Syrian public health and humanitarian crisis: A 'displacement' in global governance? Global Public Health. 2017((Mackey) Global Health Policy Institute San Diego CA USA):1-17.	Wrong study design
Akello-Ayebare G Richters JM Polderman AM et al. Healthcare-seeking strategies among displaced children in war-ridden northern Uganda: The case of malaria. Ann Trop Med Parasitol. 2010;104(5):369-76.	Wrong study design
Akil L Ahmad HA. The recent outbreaks and reemergence of poliovirus in war and conflict-affected areas. Int J Infect Dis. 2016;49((Akil Ahmad) Department of Biology/Environmental Science Jackson State University 1400 JR Lynch Street	Wrong study design
Akinyemi O. HIV knowledge and willingness to participate in new preventive technologies (NPT) trials among a Nigerian refugee population. J Acquir Immune Defic Syndr. 2009;51((Akinyemi) Department of Community Medicine University College Hospital Ibadan Nigeria):144.	Wrong study design
Al Faisal W Al Saleh Y Sen K. Syria: Public health achievements and sanctions. The Lancet. 2012;379(9833):2241.	Wrong study design
Al Gasser NA Dresden E Keeney GB et al. Status of women and infants in complex humanitarian emergencies. Journal of Midwifery and Women's Health. 2004;49(4 SUPPL.):7-13.	Wrong study design
Al Jarousha AMK El Qouqa IA El Jadba AHN et al. An outbreak of Serratia marcescens septicaemia in neonatal intensive care unit in Gaza City Palestine. J Hosp Infect. 2008;70(2):119-26.	Wrong study design
Al Qutob MF. WHO and the refugee crisis in Jordan and beyond. The Lancet Global Health. 2016;4(5):e304.	Wrong study design
Alaba OA Kashalala GT Fawole AA. Factors affecting malaria prevention and treatment decisions for children in the Democratic Republic of Congo. Am J Trop Med Hyg. 2009;81(5 SUPPL. 1):64.	Wrong study design
Alaba OA Tshiswaka-Kashalala G. Social and environmental determinants of childhood malaria and the use of itn: Implications for malaria control strategies in the democratic republic of congo. Am J Trop Med Hyg. 2010;83(5 SUPPL. 1):223.	Wrong study design
Al-Abbassi AM Ahmed S Al-Hadithi T. Cholera epidemic in Baghdad during 1999: Clinical and bacteriological profile of hospitalized cases. Eastern Mediterranean Health Journal. 2005;11(1-2):6-13.	Wrong study design
Alam Mehrjerdi Z Hosseinzadeh A Mansoori N et al. High risk sex behaviours among drug-using refugees: Implications for treatment. Sex Transm Infect. 2013;89((Mansoori) Family Research Institue Shahid Beheshti University Tehran	Wrong topic
Al-Ammouri I Ayoub F. Heart Disease in Syrian Refugee Children: Experience at Jordan University Hospital. Annals of Global Health. 2016;82(2):300-6.	Wrong study design
Alasaad S. War diseases revealed by the social media: Massive leishmaniasis outbreak in the Syrian Spring. Parasites and Vectors. 2013;6(1):no pagination.	Wrong study design
Alavi SM Alavi L Jaafari F. Outbreak investigation of needle sharing-induced malaria Ahvaz Iran. Int J Infect Dis. 2010;14(3):e240-e2.	Wrong patient populati

Alawieh A Musharrafieh U Jaber A et al. Revisiting leishmaniasis in the time of war: The Syrian conflict and the Lebanese outbreak. Int J Infect Dis. 2014;29((Ghosn) Epidemiological Surveillance Department Ministry of Public Health Beirut	Wrong study design
Alawieh A Sabra Z Langley EF et al. Assessing the impact of the Lebanese National Polio Immunization Campaign using a population-based computational model. BMC Public Health. 2017;17(1):902.	Wrong study design
al-Bargish KA. Outbreak of pertussis in Basra Iraq. Eastern Mediterranean health journal = La revue de sante de la Mediterranee orientale = al-Majallah al-ihhiyah li-sharq al-mutawassi. 1999;5(3):540-8.	Wrong setting
Albertyn C van der Plas H Hardie D et al. Silent casualties from the measles outbreak in South Africa. South African medical journal = Suid-Afrikaanse tydskrif vir geneeskunde. 2011;101(5):313-7.	Wrong study design
Alcorn T. Responding to sexual violence in armed conflict. The Lancet. 2014;383(9934):2034-7.	Wrong topic
Aldighieri S Suermondt G Toulemonde S et al. Indole non-production and antibiotic multiresistance of Vibrio cholerae 0:1 in Rwanda. Medecine tropicale : revue du Corps de sante colonial. 1997;57(3):309-10.	Wrong study design
Alemayehu A Gedefaw L Yemane T et al. Prevalence Severity and Determinant Factors of Anemia among Pregnant Women in South Sudanese Refugees	Wrong study design
Alexander Jr JP Zubair M Khan M et al. Progress and peril: poliomyelitis eradication efforts in Pakistan 1994-2013. J Infect Dis. 2014;210(suppl_1):S152-61.	Wrong study design
Al-Hindi AI Abu Shammala BM. Dientamoeba fragilis in gaza strip: A neglected protozoan parasite. Iranian Journal of Parasitology. 2013;8(2):249-55.	Wrong study design
Ali A Nisar M Idrees M et al. Prevalence of HBV infection in suspected population of conflict-affected area of war against terrorism in North Waziristan FATA Pakistan. Infect Genet Evol. 2012;12(8):1865-9.	Wrong study design
Ali A Ur Rehman T Qureshi NA et al. New endemic focus of cutaneous leishmaniasis in Pakistan and future epidemics threats. Asian Pacific Journal of Tropical Disease. 2016;6(2):155-9.	Wrong study design
Ali SI Ali SS Fesselet JF. Effectiveness of emergency water treatment practices in refugee camps in South Sudan. Bull World Health Organ. 2015;93(8):550-8.	Wrong study design
Aliev S Saparova N. Current malaria situation and its control in Tadjikistan. Med Parazitol (Mosk). 2001(1):35-7.	Wrong patient populati
Aliev SP. Malaria in the Republic of Tajikistan. Med Parazitol (Mosk). 2000(2):27-9.	Non-English
Ali-Shtayeh MS Arda HM Abu-Ghdeib SI. Epidemiological study of tinea capitis in schoolchildren in the Nablus area (West Bank). Mycoses. 1998;41(5-6):243-8.	Wrong study design
Ali-Shtayeh MS Salameh AAM Abu-Ghdeib SI et al. Prevalence of tinea capitis as well as of asymptomatic carriers in school children in Nablus area (Palestine). Mycoses. 2002;45(5-6):188-94.	Wrong study design
Al-Jawabreh A Barghuthy F Schnur LF et al. Epidemiology of cutaneous leishmaniasis in the endemic area of Jericho Palestine. Eastern Mediterranean Health Journal. 2003;9(4):805-15.	Wrong study design
Al-Kamel MA. Leishmaniasis in Yemen: A clinicoepidemiological study of leishmaniasis in central Yemen. Int J Dermatol. 2015((Al-Kamel) Regional Leishmaniasis Control Center Sana'a Yemen):no pagination.	Wrong study design
Al-Khatib IA Tabakhna H. Housing conditions and health in Jalazone Refugee Camp in Palestine. Eastern Mediterranean Health Journal. 2006;12(1-2):144-52.	Wrong study design

Al-Kubaisy WAAQ. Epidemiology of bloody diarrhoea among children less than ten years of age in BAGHD. Arch Dis Child. 2012;97((Al-Kubaisy) Community Medicine UiTM Faculty of Medicine Shah Alam Malaysia):A246.	Wrong study design
Allen DR Poirot E Wangroongsarb P et al. Findings from a rapid qualitative assessment of access to malaria prevention and treatment resources among burmese migrants in tak province thailand. Am J Trop Med Hyg. 2012;87(5 SUPPL. 1):119.	Wrong topic
Allen-Williams GM. Incidence of infections in war-time day nurseries. Lancet (London England). 1945;2(6382):825.	Wrong setting
Al-Nasrawi KK Al- Diwan JK Al-Hadithi TS et al. Viral hepatitis E outbreak in Al-Sadr city Baghdad Iraq. Eastern Mediterranean Health Journal. 2010;16(11):1128-32.	Wrong study design
Al-Saadi MAK Al-Khafaji YAK Al-Kaif LAIK. Isolation and identification of measles virus from refugees in Babylon Governorate-Iraq. Research Journal of Pharmaceutical Biological and Chemical Sciences. 2017;8(2):408-16.	Wrong topic
Al-Salem W Herricks JR Hotez PJ. A review of visceral leishmaniasis during the conflict in South Sudan and the consequences for East African countries. Parasites and Vectors. 2016;9(1):no pagination.	Wrong study design
Al-Salem WS Pigott DM Subramaniam K et al. Cutaneous Leishmaniasis and Conflict in Syria. Atlanta Georgia: Centers for Disease Control & Prevention (CDC); 2016. p. 931-3.	Wrong study design
Al-Salihi KA Hassan IQ. Lumpy Skin Disease in Iraq: Study of the Disease Emergence. Transbound Emerg Dis. 2015;62(5):457-62.	Wrong study design
Alvarez GG Clark M Altpeter E et al. Pediatric tuberculosis immigration screening in high-immigration low-incidence countries. Int J Tuberc Lung Dis. 2010;14(12):1530-7.	Wrong setting
Amela Heras C Pachon del Amo I Ibanez Marti C. Measures of the effect of vaccination in an outbreak of measles. Gaceta sanitaria / SESPAS. 1993;7(37):164-8.	Non-English
Ameratunga S Lennon D Croxson M. Rubella epidemic. N Z Med J. 1990;103(898):464.	Wrong setting
Ammar W Kdouh O Hammoud R et al. Health system resilience: Lebanon and the Syrian refugee crisis. Journal of Global Health. 2016;6(2):1-9.	Wrong study design
Anand JK. Cholera treatment in Goma. Lancet. 1995;345(8964):1568.	Wrong study design
Anderson J Doocy S Haskew C et al. The burden of malaria in post-emergency refugee sites: A retrospective study. Conflict and health. 2011;5(1):17.	Wrong study design
Andrews B Udhayashankar K Adu E et al. Intravenous artusenate vs. Quinine in the treatment of severe malaria in liberian children. Am J Trop Med Hyg. 2014;91(5 SUPPL. 1):139.	Wrong study design
Angeletti S Cella E Lai A et al. Whole-genome sequencing of Klebsiella pneumoniae MDR strain isolated in a Syrian refugee. Pathogens and Global Health. 2017((Spoto) Internal Medicine Department University Hospital Campus Bio-Medico Rome	Wrong study design
Angez M Shaukat S Alam MM et al. Genetic relationships and epidemiological links between wild type 1 poliovirus isolates in Pakistan and Afghanistan. Virol J. 2012;9((Angez Shaukat Alam	Wrong study design
Anguita M. Measles outbreak: Why now and who is most at risk. Nurse Prescribing. 2013;11(5):214-6.	Wrong setting
Ansari AS Khanani MR Abidi SH et al. Patterns of HIV infection among native and refugee Afghans. AIDS. 2011;25(11):1427-30.	Wrong study design
Antinori S Colombo V Corbellino M. Relapsing fever in young refugees from East Africa. Critical Care. 2017;21(1):205.	Wrong study design

Anuradha S Singh NP Rizvi SN et al. The 1996 outbreak of dengue hemorrhagic fever in Delhi India. The Southeast Asian journal of tropical medicine and public health. 1998;29(3):503-6.	Wrong setting
Aplogan A Batchassi E Yakoua Y et al. [An epidemic of meningococcal meningitis in the region of Savanes in Togo in 1997: research and control strategies]. Sante (Montrouge France). 1997;7(6):384-90.	Non-English
Aradaib IE Erickson BR Mustafa ME et al. Nosocomial outbreak of Crimean-Congo hemorrhagic fever Sudan. Emerg Infect Dis. 2010;16(5):837-9.	Wrong study design
Aragon M Barreto A Chambule J et al. Shigellosis in Mozambique: the 1993 outbreak rehabilitation--a follow-up study. Trop Doct. 1995;25(4):159-62.	Wrong study design
Araj GF Saade A Itani LY et al. Tuberculosis burden in Lebanon: Evolution and current status. Journal Medical Libanais. 2016;64(1):1-7.	Wrong study design
Arguedas AG Deveikis AA Marks MI. Measles. Am J Infect Control. 1991;19(6):290-8.	Wrong setting
Ari MD Guracha A Fadeel MA et al. Challenges of establishing the correct diagnosis of outbreaks of acute febrile illnesses in Africa: the case of a likely Brucella outbreak among nomadic pastoralists northeast Kenya March-July 2005. The American journal of tropical medicine and hygiene. 2011;85(5):909-12.	Wrong study design
Armstrong PK Anstey NM Kelly PM et al. Seroprevalence of Burkholderia pseudomallei in East Timorese refugees: Implications for healthcare in East Timor. Southeast Asian J Trop Med Public Health. 2005;36(6):1496-502.	Wrong setting
Arthur JD Bodhidatta L Echeverria P et al. Diarrheal disease in Cambodian children at a camp in Thailand. Am J Epidemiol. 1992;135(5):541-51.	Wrong study design
Arya N Zurbrigg S. Operation infinite injustice: Impact of sanctions and prospective war on the people of Iraq. Canadian Journal of Public Health. 2003;94(1):9-12.	Wrong topic
Arya SC. Outbreak of poliomyelitis in Angola. The Journal of infectious diseases. 2001;183(3):517-8.	Wrong setting
Assefa F Jabarkhil MZ Salama P et al. Malnutrition and mortality in Kohistan district Afghanistan April 2001. J Am Med Assoc. 2001;286(21):2723-8.	Wrong study design
Atwood KA Kennedy SB Shamblen S et al. Reducing sexual risk taking behaviors among adolescents who engage in transactional sex in post-conflict Liberia. Vulnerable Children and Youth Studies. 2012;7(1):55-65.	Wrong setting
Aziz C. Iraqi women: victims of war and sanctions. Planned parenthood challenges. 1997(1-2):34-6.	Wrong topic
Azman AS Bouhenia M Iyer AS et al. High hepatitis e seroprevalence among displaced persons in South Sudan. Am J Trop Med Hyg. 2017;96(6):1296-301.	Wrong study design
Babille M De Colombani P Guerra R et al. Post-emergency epidemiological surveillance in Iraqi-Kurdish refugee camps in Iran. Disasters. 1994;18(1):58-75.	Wrong study design
Bader JM. Listeriosis epidemic. Lancet. 1993;342(8871):607.	Wrong setting
Bahr G Costello AM Alahdab Y et al. Epidemic tuberculosis in north Lebanon. Lancet (London England). 1991;337(8747):983-4.	Wrong study design
Bailly JL Cardoso MC Labbe A et al. Isolation and identification of an enterovirus 77 recovered from a refugee child from Kosovo and characterization of the complete virus genome. Virus Res. 2004;99(2):147-55.	Wrong setting
Bajraktarevic A Maglajlija S Mahinic A et al. Specifity of tuberculosis and resistence of therapy between immigrants and bosnia-born children. Arch Dis Child. 2012;97((Mujicic Selimovic Sulejmanpasic) Pediatrics Department Clinical Medical Center	Wrong study design

Bajraktarevic A Mulalic Z Penava S et al. Assessment MMR vaccination in Bosnian refugees travellers preschool kids. Trop Med Int Health. 2009;14((Bajraktarevic Mulalic	Wrong study design
Bakacak M Serin S Aral M et al. Seroprevalance Differences of Toxoplasma Between Syrian Refugees Pregnants and Indigenous Turkish Pregnants in Kahramanmaras. Turkiye parazitolojii dergisi / Turkiye Parazitoloji Dernegi = Acta parasitologica Turcica / Turkish Society for Parasitology. 2015;39(2):94-7.	Non-English
Bamrah S Mbithi A Mermin JH et al. The impact of post-election violence on HIV and other clinical services and on mental health - Kenya 2008. Prehosp Disaster Med. 2013;28(1):43-51.	Wrong study design
Barclay WR. Indochinese refugees. JAMA : the journal of the American Medical Association. 1979;242(21):2327.	Wrong setting
Barnett B. Family planning rarely available for refugees. Network (Research Triangle Park NC). 1995;15(3):4-8.	Wrong topic
Barnett ED Christiansen D Figueira M. Seroprevalence of measles rubella and varicella in refugees. Clin Infect Dis. 2002;35(4):403-8.	Wrong study design
Barth J. Cholera in the time of civil war. Liberia. Links (New York NY). 1991;8(3):20-3.	Wrong study design
Bartkovjak M Krcmery V. Spectrum of infectious diseases among internally displaced refugees in South Sudan and north Kenya. Int J Infect Dis. 2014;21((Bartkovjak Krcmery) Mary Immaculate Clinic St. Elisabeth University (SEU) Tropical Program Yirol County Mapuordit	Wrong study design
Bartlett LA Purdin S McGinn T. Forced migrants - Turning rights into reproductive health. Lancet. 2004;363(9402):76-7.	Wrong topic
Basseri H Raeisi A Holakouie K et al. Knowledge attitudes and practices (KAP) study regarding to malaria transmission and protection among Afghan refugees and comparing with Iranian resident Southeastern Iran. Trop Med Int Health. 2009;14((Raeisi Holakouie	Wrong study design
Basseri HR Raeisi A Holakouie K et al. Malaria prevention among Afghani refugees in a malarious area southeastern Iran. Bulletin de la Societe de pathologie exotique (1990). 2010;103(5):340-5.	Wrong study design
Bassey BE Vaz RG Gasasira AN et al. Pattern of the meningococcal meningitis outbreak in Northern Nigeria 2009. International journal of infectious diseases : IJID : official publication of the International Society for Infectious Diseases. 2016;43(c3r 9610933):62-7.	Wrong study design
Batoev SD. [The organization of maternity and child care in the Buryat-Mongolskaya ASSR during the Great Patriotic War]. Problemy sotsialnoi gigieny zdravookhraneniia i istorii meditsiny / NII sotsialnoi gigieny ekonomiki i upravleniia zdravookhraneniem im NA Semashko RAMN ; AO "Assotsiatsiia 'Meditsinskaia literatura'". 2010(4):61-3.	Non-English
Baudin M Jumaa AM Jomma HJE et al. Association of Rift Valley fever virus infection with miscarriage in Sudanese women: a cross-sectional study. The Lancet Global health. 2016;4(11):e864-e71.	Wrong setting
Bayoh MN Akhwale W Ombok M et al. Malaria in Kakuma refugee camp Turkana Kenya: Facilitation of Anopheles arabiensis vector populations by installed water distribution and catchment systems. Malar J. 2011;10((Hamel) Centers for Disease Control and Prevention	Wrong topic
Bazroy J Panda P Purty AJ et al. Refugee children in India: A comparative study. Hong Kong Journal of Paediatrics. 2005;10(2):101.	Wrong study design
Bbaale E. Determinants of diarrhoea and acute respiratory infection among under-fives in uganda. Australasian Medical Journal. 2011;4(7):400-9.	Wrong study design
Bechen R Boutin JP Combes D et al. Cholera in Goma July 1994. Rev Epidemiol Sante Publique. 1996;44(4):358-63.	Non-English

Begg E Beattie S Hurley C. In the war zone. Interview by Pieter Tesch. Nursing standard (Royal College of Nursing (Great Britain) : 1987). 2006;20(24):26-7.	Wrong study design
Beldjebel I Krcmery V. Infectious diseases among Iraqi refugees in Lebanon. Int J Infect Dis. 2012;16((Krcmery) St. Elizabeth University College of Health and Social Sciences Bratislava Slovakia):e341-e2.	Duplicate
Beldjebel I. Infectious diseases among refugees in Beirut. Int J Infect Dis. 2012;16((Beldjebel) Charles Foucauld Health Ctr Beirut Lebanon):e6.	Wrong study design
Beldjebel I. Infectious diseases in refugees coming from Syria and Iraq to Lebanon. Int J Infect Dis. 2014;21((Beldjebel) St. Charles Foucauld Health Centre St. Elisabeth University Beirut Lebanon):26.	Wrong study design
Bell LM. Foreword: Primary care for refugee children. Curr Probl Pediatr Adolesc Health Care. 2014;44(7):185.	Wrong setting
Belongia EA Holtan N MacDonald KL et al. Absence of HTLV-1 infection in Southeast Asian refugees. The Southeast Asian journal of tropical medicine and public health. 1991;22(1):135-6.	Wrong setting
Benca J Kralova J Bukovinova P et al. Meningococcal meningitis among displaced and refugee camps in southern Sudan [2]. Neuroendocrinology Letters. 2007;28(SUPPL. 2):44.	Wrong study design
Benca J Kralova J Bukovinova P et al. Meningococcal meningitis among displaced and refugee camps in southern Sudan. Neuro Endocrinol Lett. 2007;28 Suppl 2(d1z 8008373):44.	Duplicate
Benjamin ER Clements C McCally M et al. The humanitarian cost of a war in Iraq. Lancet. 2003;361(9360):874.	Wrong study design
Bennett FJ Jelliffe DB Jelliffe EFP et al. The nutrition and disease pattern of children in a refugee settlement. 1968;45(5):229-46.	Wrong setting
Bergenström A. Afghanistan: HIV/AIDS vulnerability and prevention. Journal of Health Management. 2003;5(2):215-24.	Wrong study design
Berman J. Recent developments in leishmaniasis: Epidemiology diagnosis and treatment. Curr Infect Dis Rep. 2005;7(1):33-8.	Wrong study design
Bertherat E Mueller MJ Shako JC et al. Discovery of a leptospirosis cluster amidst a pneumonic plague outbreak in a miners' camp in the democratic republic of the Congo. Int J Environ Res Public Health. 2014;11(2):1824-33.	Wrong setting
Besa NC Coldiron ME Bakri A et al. Diphtheria outbreak with high mortality in northeastern Nigeria. Epidemiol Infect. 2014;142(4):797-802.	Wrong study design
Betsi N Koudou B Cissé G et al. Effect of an armed conflict on human resources and health systems in Côte d'Ivoire: prevention of and care for people with HIV/AIDS. AIDS Care. 2006;18(4):356-65.	Wrong study design
Bhatia S Dranyi T Rowley D. A social and demographic study of Tibetan refugees in India. Soc Sci Med. 2002;54(3):411-22.	Wrong study design
Bhatia S Dranyi T Rowley D. Tuberculosis among Tibetan refugees in India. Soc Sci Med. 2002;54(3):423-32.	Wrong study design
Bhootrani ML Tahir SM. Polio free Pakistan: Reality or dream? Journal of the Liaquat University of Medical and Health Sciences. 2012;11(3):122-3.	Wrong study design
Bhunia R Ramakrishnan R Hutin Y et al. Cholera outbreak secondary to contaminated pipe water in an urban area West Bengal India	Wrong setting
Biellik RJ Brown DW. Measles mortality reduction in Africa. Lancet. 2009;373 North American Edition(9668):984-5.	Wrong study design

Biko DM McQuillan BF Jesinger RA et al. Imaging of pediatric pathology during the Iraq and Afghanistan conflicts. Pediatr Radiol. 2015;45(3):439-48.	Wrong study design
Bile KM Shadoul AF Raaijmakers H et al. Learning through crisis: Development and implementation of a health cluster strategy for internally displaced persons. Eastern Mediterranean Health Journal. 2010;16(SUPPL.):S82-90.	Wrong study design
Bin Ghouth AS Amarasinghe A Letson GW. Dengue outbreak in Hadramout Yemen 2010: an epidemiological perspective. The American journal of tropical medicine and hygiene. 2012;86(6):1072-6.	Wrong study design
Birmingham ME Lee LA Ndayimirije N et al. Epidemic cholera in Burundi: Patterns of transmission in the Great Rift Valley Lake region. Lancet. 1997;349(9057):981-5.	Wrong setting
Birrie H Balcha F Bizuneh A et al. Susceptibility of Ethiopian bulinid snails to Schistosoma haematobium from Somalia. East Afr Med J. 1996;73(1):76-7.	Wrong topic
Bisaillon LM. Refugee + Support Project. Network Magazine of the Canadian Women's Health Network. 2008;10(2):25-6.	Wrong setting
Bishop D Altshuler M Scott K et al. The refugee medical exam: What you need to do. J Fam Pract. 2012;61(12):E1-E10.	Wrong setting
Bisrat F Berhane Y Mamo A et al. Morbidity pattern among refugees in Eastern Ethiopia. East Afr Med J. 1995;72(11):728-30.	Wrong study design
Blaylock JM Maranich A Bauer K et al. The seroprevalence and seroincidence of dengue virus infection in western Kenya. Travel Med Infect Dis. 2011;9(5):246-8.	Wrong study design
Blum LS Vujcic J Ram PK. Handwashing behavior change strategies in humanitarian emergency situations: The perspectives of experts. Am J Trop Med Hyg. 2014;91(5 SUPPL. 1):216.	Wrong study design
Blum LS Yemweni A Trinies V et al. A qualitative assessment of motivators and barriers to handwashing behaviors in an emergency setting in North Kivu Democratic Republic of Congo. Am J Trop Med Hyg. 2017;97(5 Supplement 1):401-2.	Extracted in WASH
Boccia D Guthmann JP Klovstad H et al. High mortality associated with an outbreak of hepatitis E among displaced persons in Darfur Sudan. Clin Infect Dis. 2006;42(12):1679-84.	Wrong study design
Boelaert M Suetens C van Soest M et al. Cholera treatment in Goma. Lancet. 1995;345(8964):1567.	Wrong study design
Bollag U. Practical evaluation of a pilot immunization campaign against typhoid fever in a Cambodian refugee camp. Int J Epidemiol. 1980;9(2):121-2.	Wrong setting
Bompangue D Giraudoux P Handschumacher P et al. Lakes as source of cholera outbreaks Democratic Republic of Congo. Emerg Infect Dis. 2008;14(5):798-800.	Wrong study design
Bompart F. [Vaccination strategies for the global eradication of poliomyelitis]. Bulletin de la Societe de pathologie exotique (1990). 2004;97(4):288-92.	Non-English
Bonn D. Infectious diseases threaten refugees entering Pakistan. The Lancet infectious diseases. 2001;1(4):214.	Wrong study design
Bonner PC Schmidt WP Belmain SR et al. Poor housing quality increases risk of rodent infestation and lassa fever in refugee camps of sierra leone. Am J Trop Med Hyg. 2007;77(1):169-75.	Wrong study design
Bos P Steele AD Peenze I et al. Sero-prevalence to hepatitis B and C virus infection in refugees from Mozambique in southern Africa. East Afr Med J. 1995;72(2):113-5.	Wrong study design
Boui M Lemnaouer A. Dermatological experience at the Moroccan mobile field hospital in Brazzaville Congo. Med Trop (Mars). 2009;69(1):13-7.	Non-English

Bouma MJ Goris M Akhtar T et al. Prevalence and clinical presentation of glucose-6-phosphate dehydrogenase deficiency in Pakistani Pathan and Afghan refugee communities in Pakistan; Implications for the use of primaquine in regional malaria control programmes. Trans R Soc Trop Med Hyg. 1995;89(1):62-4.	Wrong study design
Bouma MJ Nesbit R. Fenitrothion intoxication during spraying operations in the malaria programme for Afghan refugees in North West Frontier Province of Pakistan. Trop Geogr Med. 1995;47(1):12-4.	Wrong patient populati
Bouscaillou J Komas N Tricou V et al. Imported hepatitis e virus central african republic 2011. Emerg Infect Dis. 2013;19(2):336-7.	Wrong study design
Boussery G et al. Visceral leishmaniasis (kala-azar) outbreak in Somali refugees and Kenyan shepherds Kenya. Emerg Infect Dis. 2001;7(3 Suppl):603-4.	Wrong study design
Bower H. Arming the population against polio in Somalia. Lancet (London England). 2000;356(9225):229.	Wrong study design
Boyd AT. Tuberculosis. Refugee health care: An essential medical guide. 2014(WHO. (2012). WHO global tuberculosis report 2012. Geneva: World Health Organization: 2012. http://www.who.int/tb/publications/global_report/gtbrl2_main.pdf. Accessed Aug 2013.):53-62.	Wrong setting
Bradaric N Smoljanovic M Pavic S et al. Epidemiological and clinical characteristics of a typhoid fever outbreak in the Lasva Valley Central Bosnia spring 1994. Croat Med J. 1994;35(3):177-82.	Wrong study design
Bradaric N. Two outbreaks of typhoid fever related to the war in Bosnia and Herzegovina. Eur J Epidemiol. 1996;12(4):409-12.	Wrong study design
Breiman RF Shultz A Omollo JO et al. Cholera outbreak in kenyan refugee camp: Risk factors for illness and importance of sanitation. Am J Trop Med Hyg. 2009;80(4):640-5.	Wrong study design
Brinkmann F Benzrath S Forster J et al. Tuberculosis screening in refugees under the age of five-worth the effort? Eur Respir J. 2016;48:no pagination.	Wrong setting
Brock C Knowles M Goh S. A school and community outbreak of influenza A. Commun Dis Rep CDR Rev. 1995;5(12):R177-9.	Wrong setting
Brooks HM Paul MKJ Claude KM et al. Malaria in an internally displaced persons camp in the democratic Republic of Congo. Am J Trop Med Hyg. 2016;95(5 Supplement 1):99-100.	Wrong study design
Brown D Miller E. Facing the measles epidemic. The Practitioner. 1994;238(1544):778-81.	Wrong setting
Brown T Smith LS Oo EKS et al. Molecular surveillance for drug-resistant Plasmodium falciparum in clinical and subclinical populations from three border regions of Burma/Myanmar: Cross-sectional data and a systematic review of resistance studies. Malar J. 2012;11((Richards) Department of General Internal Medicine and Health Services Research University of California at Los Angeles 911 Broxton Ave.	Wrong study design
Brown V Abdir Issak M Rossi M et al. Epidemic of malaria in north-eastern Kenya. Lancet (London England). 1998;352(9137):1356-7.	Wrong setting
Brown V Jacquier G Bachy C et al. Management of cholera epidemics in a refugee camp. Bull Soc Pathol Exot. 2002;95(5):351-4.	Non-English
Brown VJ. Reconstructing the environment in Iraq. Environ Health Perspect. 2004;112(8):A464.	Wrong study design
Browne LB Menkir Z Kahi V et al. Notes from the field: hepatitis E outbreak among refugees from South Sudan - Gambella Ethiopia April 2014-January 2015. MMWR Morbidity and mortality weekly report. 2015;64(19):537.	Wrong study design

Bruns L Spiegel P. Displaced persons and HIV care: Challenges and solutions. South Afr J HIV Med. 2007(26):42-3.	Wrong study design
Brusin S. The Communicable Disease Surveillance System in the Kosovar refugee camps in the former Yugoslav Republic of Macedonia April-August 1999. J Epidemiol Community Health. 2000:52-7.	Duplicate
Brusin S. The Communicable Disease surveillance system in the Kosovar refugee camps in the former Yugoslav Republic of Macedonia April-August 1999. J Epidemiol Community Health. 2000;54(1):52-7.	Wrong study design
Buchwald D Collier AC Lukehart SA et al. Evaluation of cerebrospinal fluid in Southeast Asian refugees with reactive serologic tests for syphilis. West J Med. 1996;165(5):289-93.	Wrong study design
Buitrago LS Brochero HL McKeon SN et al. First published record of urban malaria in Puerto Gaitan Meta Colombia. Mem Inst Oswaldo Cruz. 2013;108(8):1045-50.	Wrong study design
Burans JP Sharp T Wallace M et al. Threat of hepatitis E virus infection in Somalia during Operation Restore Hope. Clin Infect Dis. 1994;18(1):100-2.	Wrong study design
Burki T. Infectious diseases in Malian and Syrian conflicts. The Lancet infectious diseases. 2013;13(4):296-7.	Wrong study design
Burki T. Yemen's neglected health and humanitarian crisis. The Lancet. 2016;387(10020):734-5.	Wrong study design
Burkle Jr FM. Pediatric issues in complex emergencies. Ambulatory Child Health. 2001;7(2):119-26.	Wrong study design
Burns DA Wood CB. Cholera treatment in Goma. Lancet. 1995;345(8964):1568.	Wrong study design
Burton A John-Leader F. Are we reaching refugees and internally displaced persons? Bull World Health Organ. 2009;87(8):638-9.	Wrong study design
Bush K. Polio war and peace. Bull World Health Organ. 2000;78(3):281-2.	Wrong study design
Busico KM Marshall KL Ksiazek TG et al. Prevalence of IgG antibodies to Ebola virus in individuals during an Ebola outbreak Democratic Republic of the Congo 1995. J Infect Dis. 1999;179(SUPPL. 1):S102-S7.	Wrong study design
Butcher K Biggs BA Leder K et al. Understanding of latent tuberculosis its treatment and treatment side effects in immigrant and refugee patients. BMC Res Notes. 2013;6((Butcher Biggs	Wrong setting
Bwire G Mwesawina M Baluku Y et al. Cross-border cholera outbreaks in Sub-Saharan Africa the mystery behind the silent illness: What needs to be done? PLoS One. 2016;11(6):no pagination.	Wrong study design
Bytchenko B. Poliomyelitis in the eastern European countries--achievements and remaining problems. Public Health Rev. 1993;21(1-2):51-63.	Wrong study design
Cain KP Marano N Kamene M et al. The movement of multidrug-resistant tuberculosis across borders in East Africa needs a regional and global solution. PLoS Med. 2015;12(2):e1001791.	Wrong study design
Caldwell JP Kain BF McDonald RC. A canadian medical team in ethiopia. Canadian family physician Medecin de famille canadien. 1985;31(blo 0120300):2115-7.	Wrong setting
Callaghan MP Immerman B. PHS mission to Goma Zaire. Public Health Rep. 1995;110(1):95-9.	Wrong study design
Campbell H Nair H. Humanitarian crises due to natural disasters and armed conflict. J R Coll Physicians Edinb. 2014;44(3):216-7.	Wrong study design
Campbell S. Reproductive health interventions for displaced populations. Primary Health Care. 2003;13(3):39-42.	Wrong study design
Caneco RA Ruiz RM Lopez HT. Sexual violence against women in armed conflict settings. Archives of Women's Mental Health. 2011;14((Ruiz Lopez) Hospital Clinico San Carlos Madrid Spain):S98.	Wrong topic

Caplan AL Curry DR. Refugees humanitarian aid and the right to decline vaccinations. J Med Ethics. 2015;41(3):276-7.	Wrong study design
Carlton-Ford S. The impact of war adult HIV/AIDS and militarization on young children's mortality. Sociological studies of children and youth: Special international volume Vol 10. 2005(Zwi A. (1996). Numbering the dead: Counting the casualties of war. In: H. Bradby (Ed.) Defining Violence: Understanding the Causes and Effects of Violence (pp. 99-124). Aldershot: Avebury.):231-55.	Wrong study design
Carrara VI Hogan C De Pree C et al. Improved pregnancy outcome in refugees and migrants despite low literacy on the Thai-Burmese border: Results of three cross-sectional surveys. BMC Pregnancy Childbirth. 2011;11((Nosten McGready) Faculty of Tropical Medicine Mahidol University	Wrong topic
Carrara VI Zwang J Ashley EA et al. Changes in the treatment responses to artesunate-mefloquine on the northwestern border of Thailand during 13 years of continuous deployment. PLoS One. 2009;4(2):e4551.	Wrong study design
Carrion Martin AI Bil K Salumu P et al. Mortality Rates above Emergency Threshold in Population Affected by Conflict in North Kivu Democratic Republic of Congo July 2012-April 2013. PLoS Negl Trop Dis. 2014;8(9):no pagination.	Wrong study design
Casey N. Caring for Romania appeal: against all the odds. Nursing standard (Royal College of Nursing (Great Britain) : 1987). 1990;4(38):17-9.	Wrong setting
Catchpole M Coulombier D. Refugee crisis demands European union-wide surveillance! Eurosurveillance. 2015;20(45):no pagination.	Wrong study design
Cavallo JD Niel L Talarmin A et al. Sensitivity to antibiotics of epidemic strains of Vibrio cholerae and Shigella dysenteriae 1 isolated in Rwanda refugee camps in Zaire. Med Trop (Mars). 1995;55(4):351-3.	Non-English
Cella E Ceccarelli G Vita S et al. First epidemiological and phylogenetic analysis of Hepatitis B virus infection in migrants from Mali. J Med Virol. 2017;89(4):639-46.	Wrong setting
Centers for Disease C Prevention. Accelerated measles control--Cambodia 1999-2002. MMWR Morbidity and mortality weekly report. 2003;52(1):4-6.	Wrong setting
Centers for Disease C Prevention. Cholera outbreak--southern Sudan 2007. MMWR Morbidity and mortality weekly report. 2009;58(13):337-41.	Wrong setting
Centers for Disease C Prevention. Enhanced medical assessment strategy for Barawan Somali refugees--Kenya 1997. MMWR Morbidity and mortality weekly report. 1998;46(52-53):1250-4.	Wrong setting
Centers for Disease C Prevention. Health status of displaced persons following Civil War--Burundi December 1993-January 1994. MMWR Morbidity and mortality weekly report. 1994;43(38):701-3.	Duplicate
Centers for Disease C Prevention. Progress toward poliomyelitis eradication--Nigeria January 2007-August 12 2008. MMWR Morbidity and mortality weekly report. 2008;57(34):942-6.	Wrong topic
Centers for Disease C Prevention. Vaccination services in postwar Iraq May 2003. MMWR Morbidity and mortality weekly report. 2003;52(31):734-5.	Wrong topic
Centers for Disease C Prevention. Investigation of hepatitis e outbreak among refugees - upper nile South Sudan 2012-2013. MMWR Morb Mortal Wkly Rep. 2013;62(29):581-6.	Wrong study design
Centers for Disease C Prevention. Measles mortality reduction -- West Africa 1996-2002. MMWR Morb Mortal Wkly Rep. 2004;53(2):28-30.	Wrong setting
Centers for Disease C Prevention. Notes from the field: outbreak of poliomyelitis - somalia and kenya may 2013. MMWR Morb Mortal Wkly Rep. 2013;62(23):484-.	Wrong study design

Centers for Disease C Prevention. Progress toward measles control -- African region 2001-2008. MMWR Morb Mortal Wkly Rep. 2009;58(37):1036-41.	Wrong setting
Centers for Disease C Prevention. Progress toward poliomyelitis eradication - Chad January 2011-August 2012. MMWR Morb Mortal Wkly Rep. 2012;61(42):858-62.	Wrong setting
Centers for Disease C Prevention. Progress toward poliomyelitis eradication -- Nigeria January 2008-July 2009. MMWR Morb Mortal Wkly Rep. 2009;58(41):1150-4.	Wrong setting
Centers for Disease C Prevention. Rift Valley fever -- East Africa 1997-1998. MMWR Morb Mortal Wkly Rep. 1998;47(13):261-4.	Wrong study design
Centers for Disease C Prevention. Rift Valley fever outbreak -- Kenya November 2006-January 2007. MMWR Morb Mortal Wkly Rep. 2007;56(4):73-6.	Wrong study design
Chaker E Kremer M Kien TT. [14 cases of Entamoeba polecki in refugees from South-East Asia: remarks on the morphological aspect of the parasite]. Bull Soc Pathol Exot Filiales. 1982;75(5):484-90.	Wrong setting
Challa AA. Tuberculosis Incidence in Immigrants and Refugees. Philadelphia Pennsylvania: American College of Physicians; 2015. p. 149-50.	Wrong setting
Challa AA. Tuberculosis incidence in immigrants and refugees: To the editor. Ann Intern Med. 2015;163(2):149-50.	Wrong study design
Chaloner EJ Duckett J Lewin J. Paediatric rectal prolapse in Rwanda. J R Soc Med. 1996;89(12):688-9.	Wrong study design
Chan YC Salahuddin NI Khan J et al. Dengue haemorrhagic fever outbreak in Karachi Pakistan 1994. Trans R Soc Trop Med Hyg. 1995;89(6):619-20.	Wrong study design
Chanda E Govere JM Macdonald MB et al. Integrated vector management: A critical strategy for combating vector-borne diseases in South Sudan. Malar J. 2013;12(1):no pagination.	Wrong study design
Chandrasena TGAN Hapuarachchi HC Dayanath MYD et al. Intestinal parasites and the growth status of internally displaced children in Sri Lanka. Trop Doct. 2007;37(3):163-5.	Wrong study design
Chaparro P Padilla J Vallejo AF et al. Characterization of a malaria outbreak in Colombia in 2010. Malar J. 2013;12(1):no pagination.	Wrong study design
Charchuk R Paul MKJ Claude KM et al. Burden of malaria is higher among children in an internal displacement camp compared to a neighbouring village in the Democratic Republic of the Congo. Malar J. 2016;15(1):no pagination.	Wrong study design
Chaveepojnkamjorn W Pichainarong N. Malaria infection among the migrant population along the Thai-Myanmar border area. The Southeast Asian journal of tropical medicine and public health. 2004;35(1):48-52.	Wrong patient populati
Chaves NJ Mc Grath CM Walker KM et al. Approaches to management of refugees from ebola affected areas. Intern Med J. 2015;45((Paxton) Department of Immigrant Health Royal Children's Hospital Melbourne	Wrong study design
Cheah PY Lwin KM Nosten F et al. Key success indicators of a community engagement strategy: The case of a tropical medicine research unit on the Thai-Myanmar border. Am J Trop Med Hyg. 2015;93(4 Supplement):408-9.	Wrong topic
Checchi F Cox J Balkan S et al. Malaria epidemics and interventions Kenya Burundi	Wrong study design
Chelwa NM Likwa RN Banda J. Under-five mortality among displaced populations in Meheba refugee camp Zambia 2008-2014. Archives of public health = Archives belges de sante publique. 2016;74(9208826):49.	Wrong study design

Chen C. Rebellion against the polio vaccine in Nigeria: implications for humanitarian policy. Afr Health Sci. 2004;4(3):205-7.	Wrong study design
Chen MI von Roenne A Souare Y et al. Reproductive health for refugees by refugees in Guinea II: sexually transmitted infections. Conflict and health. 2008;2(101286573):14.	Wrong study design
Cherian P Junckerstorff RK Rosen D et al. Late-stage human African trypanosomiasis in a Sudanese refugee. Med J Aust. 2010;192(7):417-9.	Wrong study design
Cherian S Burgner DP Cook AG et al. Associations between helicobacter pylori infection co-morbid infections gastrointestinal symptoms	Wrong setting
Cherian S Fagan JM Thambiran A et al. Severe Plasmodium falciparum malaria in refugee children despite reported predeparture antimalarial treatment. The Medical journal of Australia. 2006;185(11-12):611.	Wrong setting
Cherkaoui I Fakhreddine A El Massaoudi K et al. Investigation of a measles epidemic in Kouf Tetouan Province Morocco. Eastern Mediterranean Health Journal. 1997;3(3):493-500.	Non-English
Chi PC Bulage P Urdal H et al. Perceptions of the effects of armed conflict on maternal and reproductive health services and outcomes in Burundi and Northern Uganda: A qualitative study. BMC Int Health Hum Rights. 2015;15(1):no pagination.	Wrong study design
Chironna M Germinario C Lopalco PL et al. Prevalence rates of viral hepatitis infections in refugee Kurds from Iraq and Turkey. Infection. 2003;31(2):70-4.	Wrong study design
Chironna M Germinario C Lupalco PL et al. Prevalence of hepatitis virus infections in Kosovar refugees. Int J Infect Dis. 2001;5(4):209-13.	Wrong setting
Cholera outbreak -- southern Sudan 2007. MMWR Morb Mortal Wkly Rep. 2009;58(13):337-41.	Wrong study design
Cholera outbreak among Rwandan refugees -- Democratic Republic of Congo April 1997. MMWR Morb Mortal Wkly Rep. 1998;47(19):389-91.	Wrong study design
Cholera outbreak. Improved surveillance care could have prevented many deaths said report. Health Letter on the CDC. 1998:7-.	Wrong study design
Clark C. Treatment (malaria): refugee screening and therapy is broadened from individual to population. Health Letter on the CDC. 1998:5-6.	Wrong setting
Cohen-Dar M Ore L Levi H et al. The IDF evacuation from Southern Lebanon in 2000: Impact on the delivery of health care to displaced Lebanese population. Harefuah. 2003;142(7):520.	Non-English
Communicable disease control in complex emergencies. Wkly Epidemiol Rec. 2000;75(50):409-.	Duplicate
Coninx R. Tuberculosis in complex emergencies. Bull World Health Organ. 2007;85(8):637-40.	Wrong study design
Conly JM Johnston BL. The infectious diseases implications of the "Lost Boys and Girls of Sudan". Canadian Journal of Infectious Diseases and Medical Microbiology. 2008;19(3):215-6.	Wrong study design
Cookson S Waldman R Gushulak B et al. Immigrant and refugee health. Emerg Infect Dis. 1998;4(3):427-8.	Wrong study design
Cooper K. A New Zealand doctor visits a Syrian refugee camp in Iraqi Kurdistan. N Z Med J. 2013;126(1382):113-5.	Wrong study design
Coppola CP Leininger BE Rasmussen TE et al. Children treated at an expeditionary military hospital in Iraq. Arch Pediatr Adolesc Med. 2006;160(9):972-6.	Wrong topic
Cornier N Schilperoord M Spiegel P et al. Access to HIV prevention care and treatment in refugee camps setting in 21 countries: Review of key indicators. J Int AIDS Soc. 2012;15((Doraiswamy) UNHCR Nairobi	Wrong study design

Coronado F Musa N Ahmed El Tayeb ES et al. Retrospective measles outbreak investigation: Sudan 2004. J Trop Pediatr. 2006;52(5):329-34.	Wrong study design
Cousins S. Experts disagree over necessity of hepatitis E vaccine in Nepal. BMJ (Clinical research ed). 2015;350((Cousins) Assam):h3393.	Wrong study design
Cousins S. Experts sound alarm as Syrian crisis fuels spread of tuberculosis. BMJ (Online). 2014;349((Cousins) BeirutLebanon):no pagination.	Wrong setting
Couto TB Farhat SC Reid T et al. Mortality in a pediatric secondary-care hospital in post-conflict Liberia in 2009. Einstein (Sao Paulo Brazil). 2013;11(4):413-20.	Wrong study design
Coutts A Fouad FM Abbara A et al. Responding to the Syrian health crisis: The need for data and research. The Lancet Respiratory Medicine. 2015;3(3):e8-e9.	Wrong study design
Coutts A Fouad FM Batniji R. Assessing the Syrian health crisis: The case of Lebanon. The Lancet. 2013;381(9875):e9.	Wrong study design
Coutts A Fouad FM. Response to Syria's health crisis-Poor and uncoordinated. The Lancet. 2013;381(9885):2242-3.	Wrong study design
Coutts A McKee M Stuckler D. The emerging Syrian health crisis. The Lancet. 2013;381(9865):e6-e7.	Wrong study design
Crabbe M. Taking services to refugees in Ghana. Planned parenthood challenges / International Planned Parenthood Federation. 1994(1):26-7.	Wrong topic
Creamer KM Edwards MJ Shields CH et al. Pediatric wartime admissions to US military combat support hospitals in Afghanistan and Iraq: Learning from the first 2000 admissions. Journal of Trauma - Injury Infection and Critical Care. 2009;67(4):762-8.	Wrong topic
Creasey A Giha H Hamad AA et al. Eleven years of malaria surveillance in a Sudanese village highlights unexpected variation in individual disease susceptibility and outbreak severity. Parasitology. 2004;129(3):263-71.	Wrong setting
Crogan J Gunasekera H Wood N et al. Management of old world cutaneous leishmaniasis in refugee children. Pediatr Infect Dis J. 2010;29(4):357-9.	Wrong setting
Cronin AA Shrestha D Spiegel P et al. Quantifying the burden of disease associated with inadequate provision of water and sanitation in selected sub-Saharan refugee camps. Journal of Water and Health. 2009;7(4):557-68.	Wrong study design
Cropley L. Malaria treatment seeking practices among mothers in rural refugee villages in Belize Central America: a qualitative study. Int Q Community Health Educ. 2003;22(1&2):3-16.	Wrong study design
Crowe S. Malaria outbreak hits refugees in Tanzania. Lancet. 1997;350(9070):41.	Wrong study design
Cummins P. Access to health care in the Western Cape. Lancet. 2002;360(SUPPL. 1):s49-s50.	Wrong topic
Dahab M Spiegel PB Njogu PM et al. Changes in HIV-related behaviours knowledge and testing among refugees and surrounding national populations: A multicountry study. AIDS Care - Psychological and Socio-Medical Aspects of AIDS/HIV. 2013;25(8):998-1009.	Wrong topic
Dajer T. The deadliest wake-up call. Cholera. Links (New York NY). 1992;9(2):7-25.	Wrong setting
Dalhat MM Isa AN Nguku P et al. Descriptive characterization of the 2010 cholera outbreak in Nigeria. BMC Public Health. 2014;14((Dalhat Isa Nguku	Wrong setting
Daniels NA Simons SL Rodrigues A et al. First do no harm: making oral rehydration solution safer in a cholera epidemic. The American journal of tropical medicine and hygiene. 1999;60(6):1051-5.	Wrong setting
Dao S Konate I Oumar AA et al. Cholera epidemics in Mali between 1995 and 2004. Sante publique (Vandoeuvre-les-Nancy France). 2009;21(3):263-9.	Non-English

Daoud W. Control of an outbreak of BCG complications in Gaza. Respirology (Carlton Vic). 2003;8(3):376-8.	Wrong study design
Daum LT Shaw MW Klimov AI et al. Influenza A (H3N2) outbreak Nepal. Emerg Infect Dis. 2005;11(8):1186-91.	Wrong study design
David AM Steering Committee for P Control of Infectious D. Hepatitis A outbreaks--methods of intervention in South-East Asian countries. International journal of infectious diseases : IJID : official publication of the International Society for Infectious Diseases. 2004;8(4):201-9.	Wrong setting
De La Fuente IG Wagner N Siegrist CA et al. Tetanus immunity as a surrogate for past diphtheria-tetanus-pertussis immunization in migrant children. Pediatr Infect Dis J. 2013;32(3):274-7.	Wrong setting
De La Hoz F Osorio B Narvaez J. Vaccination coverage in colombia opportunity and factors associated: Results from a national survey. Value Health. 2013;16(3):A97.	Wrong study design
de Serres G Boulianne N Ratnam S et al. Effectiveness of vaccination at 6 to 11 months of age during an outbreak of measles. Pediatrics. 1996;97(2):232-5.	Wrong setting
de Souza DK Sesay S Moore MG et al. No evidence for lymphatic filariasis transmission in big cities affected by conflict related rural-urban migration in Sierra Leone and Liberia. PLoS Negl Trop Dis. 2014;8(2):e2700.	Wrong setting
Debes JD. Hepatitis B in refugees guessing the prevalence. Hepatology. 2010;52(2):802-3.	Wrong setting
Dechen T Jaffe L. Reproductive health naivety and perceived gender inequities among Tibetan refugee adolescent girls in India. J Adolesc Health. 2011;48(2 SUPPL. 1):S113.	Wrong study design
Decludt B Pecoul B Biberson P et al. Malaria surveillance among the displaced Karen population in Thailand April 1984 to February 1989 Mae Sot Thailand. The Southeast Asian journal of tropical medicine and public health. 1991;22(4):504-8.	Wrong study design
Del Viso N. UNDP supports HIV / AIDS / STD project for war-torn south Sudan -- a special report. UNDP news : networking publication of UNDP staff worldwide United Nations Development Programme. 1997(101088299):21.	Wrong study design
Demirtas U Ozden A. Syrian refugees: Health services support and hospitality in Turkey. Public Health. 2015;129(11):1549-50.	Wrong study design
Denburg A Rashid M Brophy J et al. Initial health screening results for Karen refugees: a retrospective review. Canada communicable disease report = Releve des maladies transmissibles au Canada. 2007;33(13):16-22.	Wrong setting
Desenclos JC Michel D Tholly F et al. Mortality trends among refugees in Honduras 1984-1987. Int J Epidemiol. 1990;19(2):367-73.	Wrong study design
Devi S. Health services overwhelmed in northern Iraq. The Lancet. 2014;384(9944):650.	Wrong study design
Di Perri G Cazzadori A Vento S et al. Tuberculosis among refugees and displaced people at the Burundi-Rwanda border. Clin Infect Dis. 1998;26(2):500-1.	Wrong study design
Diallo MP. Child and war: A challenge for paediatricians. Arch Pediatr. 2005;12(6):877-9.	Non-English
Dodd R. Rwanda one year on... malnutrition war zones. Nurs Times.91(19):42-3.	Wrong setting
Dodge CP. Health implications of war in Uganda and Sudan. Soc Sci Med. 1990;31(6):691-8.	Wrong study design
Doganay M Demiraslan H. Refugees of the Syrian Civil War: Impact on Reemerging Infections Health Services and Biosecurity in Turkey. Health security. 2016;14(4):220-5.	Wrong study design
Doocy S Burnham G. Point-of-use water treatment and diarrhoea reduction in the emergency context: An effectiveness trial in Liberia. Trop Med Int Health. 2006;11(10):1542-52.	Extracted in WASH

Doocy S Lyles E Akhu-Zaheya L et al. Health service access and utilization among Syrian refugees in Jordan. International Journal for Equity in Health. 2016;15(1):no pagination.	Wrong study design
Doocy S Lyles E Akhu-Zaheya L et al. Health service access and utilization among Syrian refugees in Jordan. International Journal for Equity in Health. 2016;16:1-15.	Duplicate
Doocy S Lyles E Hanquart B et al. Prevalence care-seeking and health service utilization for non-communicable diseases among Syrian refugees and host communities in Lebanon. Conflict and health. 2016;10(101286573):21.	Wrong study design
Dorlencourt F Legros D Paquet C et al. Effectiveness of mass vaccination with WC/rBS cholera vaccine during an epidemic in Adjumani district Uganda. Bull World Health Organ. 1999;77(11):949-50.	Wrong study design
Doshi RH Mukadi P Shidi C et al. Field evaluation of measles vaccine effectiveness among children in the Democratic Republic of Congo. Vaccine. 2015;33(29):3407-14.	Wrong study design
Dost AG Muslim M. Malaria in Afghanistan. Med Parazitol (Mosk). 2001(1):42-3.	Non-English
Dracunculiasis eradication: global surveillance summary 2016. Wkly Epidemiol Rec. 2017;92(20):269-86.	Wrong study design
Draebel T Gueth Kueil B. Lay perceptions of malaria and therapeutic itinerary of resettled pregnant women in South Sudan. International Health. 2014;6(4):317-21.	Wrong study design
Draebel T Kueil BG Meyrowitsch DW. Prevalence of malaria and use of malaria risk reduction measures among resettled pregnant women in South Sudan. International Health. 2013;5(3):211-6.	Wrong study design
Du R Hotez PJ Al-Salem WS et al. Old World Cutaneous Leishmaniasis and Refugee Crises in the Middle East and North Africa. PLoS Negl Trop Dis. 2016;10(5):no pagination.	Wrong study design
Duffy PE Le Guillouzic H Gass RF et al. Murine typhus identified as a major cause of febrile illness in a camp for displaced Khmers in Thailand. Am J Trop Med Hyg. 1990;43(5):520-6.	Wrong study design
Dunn G. The impact of climate variability and conflict on childhood diarrhea and malnutrition in West Africa. Dissertation Abstracts International: Section B: The Sciences and Engineering. 2016;77(6-B(E)):No-Specified.	Wrong study design
Dyer O. Aid agencies launch appeal for Iraqi refugees while cholera spreads in northern Iraq. BMJ (Clinical research ed). 2007;335(7621):637.	Wrong study design
Dyer O. Cholera epidemic threatens Sierra Leone. BMJ (Clinical research ed). 1995;311(6997):77.	Wrong study design
Dyer O. Health risks emerge as ceasefire allows some humanitarian relief. BMJ: British Medical Journal (International Edition). 2006;333(7564):369-.	Wrong study design
Elduma AH Zein MMA Karlsson M et al. A Single Lineage of Hepatitis E Virus Causes Both Outbreaks and Sporadic Hepatitis in Sudan. Viruses. 2016;8(10).	Wrong study design
Elhag WI Saeed HA Omer EFE et al. Prevalence of rotavirus and adenovirus associated with diarrhea among displaced communities in Khartoum Sudan. BMC Infect Dis. 2013;13(1):no pagination.	Wrong study design
Elias CJ Alexander BH Sokly T. Infectious disease control in a long-term refugee camp: The role of epidemiologic surveillance and investigation. Am J Public Health. 1990;80(7):824-8.	Wrong study design
Engels D Madaras T Nyandwi S et al. Epidemic dysentery caused by Shigella dysenteriae type 1: A sentinel site surveillance of antimicrobial resistance patterns in Burundi. Bull World Health Organ. 1995;73(6):787-91.	Wrong study design
Enserink M. Infectious diseases. Guinea worm eradication at risk in South Sudanese war. Science (New York NY). 2014;343(6168):236.	Wrong study design

Enwereji EE. Assessing interventions available to internally displaced persons in Abia State Nigeria. Libyan J Med. 2009;4(1):17-22.	Wrong study design
Eono P Migliani R Philippe B et al. Burundi: Humanitary mission (January-April 1994). Med Trop (Mars). 1995;55(2):172-7.	Non-English
Fabiani M Ayella EO Ble C et al. Increasing HIV-1 prevalence among pregnant women living in rural areas of the Gulu district (North Uganda). AIDS. 2001;15(17):2330-1.	Wrong study design
Fan CK Liao CW Wu MS et al. Seroepidemiology of Toxoplasma gondii Infection among Chinese Aboriginal and Han People Residing in Mountainous Areas of Northern Thailand. J Parasitol. 2003;89(6):1239-42.	Wrong study design
Faulde MK Heyl G Amirih ML. Zoonotic cutaneous leishmaniasis Afghanistan [16]. Emerg Infect Dis. 2006;12(10):1623-4.	Wrong study design
Faulde MK Hoffmann R Fazilat KM et al. Epidemiology of Plasmodium falciparum and P. vivax malaria endemic in northern Afghanistan. J Egypt Soc Parasitol. 2008;38(3):679-92.	Wrong study design
Feikin DR Adazu K Obor D et al. Mortality and health among internally displaced persons in western Kenya following post-election violence 2008: Novel use of demographic surveillance. Bull World Health Organ. 2010;88(8):601-8.	Wrong study design
Fish SR. Federal district court frees Haitian refugees. The Journal of law medicine & ethics : a journal of the American Society of Law Medicine & Ethics. 1993;21(2):258-60.	Wrong setting
Fontanet AL McCauley RGK Coyette Y et al. Incidence management and outcome of childhood empyema: A prospective study of children in Cambodian refugee camps. Am J Trop Med Hyg. 1993;49(6):789-98.	Wrong study design
Ford-Jones L Law B. Issues related to infectious disease and immunization status of immigrant children including immigrants refugees and international adoptees. The Canadian journal of infectious diseases = Journal canadien des maladies infectieuses. 1993;4(2):75-8.	Wrong setting
Formenty P Libama F Epelboin A et al. Outbreak of Ebola hemorrhagic fever in the Republic of the Congo 2003: a new strategy? Medecine tropicale : revue du Corps de sante colonial. 2003;63(3):291-5.	Non-English
Formenty P Muntasir MO Damon I et al. Human monkeypox outbreak caused by novel virus belonging to Congo Basin clade Sudan 2005. Emerg Infect Dis. 2010;16(10):1539-45.	Wrong study design
Fournier AM Dodard M. The health care delivery crisis in Haiti. Fam Med. 1997;29(9):666-9.	Wrong setting
Fowler J. Beyond humanitarian bandages - Confronting genocide in Sudan. N Engl J Med. 2004;351(25):2574-6.	Wrong study design
Fox PG Kumchum S. Caring for Myanmar refugees in Thailand. Int Nurs Rev. 1996;43(5):154-8.	Wrong topic
Francis J Mutch RC Rutherford DM et al. Universal paediatric refugee health screening. J Paediatr Child Health. 2012;48(11):1048-9.	Wrong setting
Frew EMS. The case against HIV antibody testing of refugees and immigrants (II). CMAJ. 1990;142(10):1037.	Wrong setting
Furst T Raso G Acka CA et al. Dynamics of socioeconomic risk factors for neglected tropical diseases and malaria in an armed conflict. PLoS Negl Trop Dis. 2009;3(9):no pagination.	Wrong study design
Gargano LM Hajjeh R Cookson ST. Pneumonia prevention: Cost-effectiveness analyses of two vaccines among refugee children aged under two years Haemophilus influenzae type b-containing and pneumococcal conjugate vaccines during a humanitarian emergency Yida camp	Wrong study design

Gargano LM Tate JE Parashar UD et al. Comparison of impact and cost-effectiveness of rotavirus supplementary and routine immunization in a complex humanitarian emergency Somali case study. Conflict and health. 2015;9(101286573):5.	Wrong study design
Gaspar M Leite F Brumana L et al. Epidemiology of meningococcal meningitis in Angola 1994-2000. Epidemiol Infect. 2001;127(3):421-4.	Wrong study design
Gauker ED Covey DC Emens-Hesslink KE et al. A descriptive analysis of patient encounter data from the Fleet Hospital FIVE humanitarian relief mission in Haiti. Mil Med. 2000;165(5):337-45.	Wrong study design
Gayer M Watson JT Connolly MA. Displaced populations and pandemic influenza. Lancet Infect Dis. 2006;6(12):755-6.	Wrong study design
Gbakima AA Konteh R Kallon M et al. Intestinal protozoa and intestinal helminthic infections in displacement camps in Sierra Leone. Afr J Med Med Sci. 2007;36(1):1-9.	Wrong study design
Germani Y Quilici ML Glaziou P et al. Emergence of cholera in the Central African Republic. Eur J Clin Microbiol Infect Dis. 1998;17(12):888-90.	Wrong study design
Gessner BD. Mortality rates causes of death and health status among displaced and resident populations of Kabul Afghanistan. J Am Med Assoc. 1994;272(5):382-5.	Wrong study design
Ghanchi NK Shakoor S Thaver AM et al. Current situation and challenges in implementing Malaria control strategies in Pakistan. Crit Rev Microbiol. 2016;42(4):588-93.	Wrong study design
Ghebreyesus TA Witten KH Getachew A et al. The community-based malaria control programme in Tigray northern Ethiopia. A review of programme set-up activities	Wrong setting
Gilder ME Zin TW Wai NS et al. Gestational diabetes mellitus prevalence in Maela refugee camp on the Thai-Myanmar border: a clinical report. Global health action. 2014;7((Nosten McGready) Shoklo Malaria Research Unit Mahidol-Oxford Tropical Medicine Research Unit	Wrong setting
Ginosar Y Shapira SC. The role of an anaesthetist in a field hospital during the cholera epidemic among Rwandan refugees in Goma. Br J Anaesth. 1995;75(6):810-6.	Wrong topic
Girdauskas GM. The case against HIV antibody testing of refugees and immigrants (I). CMAJ. 1990;142(10):1037.	Wrong study design
Githui WA Hawken MP Juma ES et al. Surveillance of drug-resistant tuberculosis and molecular evaluation of transmission of resistant strains in refugee and non-refugee populations in North-Eastern Kenya. Int J Tuberc Lung Dis. 2000;4(10):947-55.	Wrong study design
Godue CB Gyorkos TW. Intestinal parasites in refugee claimants: A case study for selective screening. Canadian Journal of Public Health. 1990;81(3):191-5.	Wrong study design
Gould LH Osman MS Farnon EC et al. An outbreak of yellow fever with concurrent chikungunya virus transmission in South Kordofan Sudan 2005. Trans R Soc Trop Med Hyg. 2008;102(12):1247-54.	Wrong setting
Graham K Rehman H Ahmad M et al. Tents pre-treated with insecticide for malaria control in refugee camps: An entomological evaluation. Malar J. 2004;3((Graham Rowland) Infect. and Trop. Dis. Department London Sch. of Hyg. and Trop. Med.	Wrong study design
Graham K. New tools to control malaria in refugee camps. Journal of The Royal Society for the Promotion of Health. 2004;124(6):253-5.	Wrong study design
Grais RF Dubray C Gerstl S et al. Unacceptably high mortality related to measles epidemics in Niger Nigeria and Chad. PLoS Med. 2007;4(1):e16.	Wrong study design

Grandesso F Sanderson F Kruijt J et al. Mortality and malnutrition among populations living in South Darfur Sudan: results of 3 surveys September 2004. JAMA : the journal of the American Medical Association. 2005;293(12):1490-4.	Wrong study design
Gray GC Rodier GR Matras-Maslin VC et al. Serologic evidence of respiratory and rickettsial infections among Somali refugees. Am J Trop Med Hyg. 1995;52(4):349-53.	Wrong study design
Grbic M Ilic VL Baros S et al. Vulnerability to HIV of internally displaced persons in the Republic of Serbia. BMC Infect Dis. 2014;14(Supplement 2).	Wrong study design
Green A. Aid groups warn of "catastrophic" health crisis in CAR. The Lancet. 2014;383(9920):860.	Wrong study design
Green A. Violence in Burundi triggers refugee crisis. The Lancet. 2015;386(9994):639-40.	Wrong study design
Grout L Minetti A Hurtado N et al. Measles in Democratic Republic of Congo: an outbreak description from Katanga 2010-2011. BMC Infect Dis. 2013;13(1):232-.	Wrong study design
Guerin PJ Brasher C Baron E et al. Case management of a multidrug-resistant Shigella dysenteriae serotype 1 outbreak in a crisis context in Sierra Leone 1999-2000. Trans R Soc Trop Med Hyg. 2004;98(11):635-43.	Wrong setting
Gupta RK Van Vugt M Paiphun L et al. Short report: No evidence of cardiotoxicity of atovaquone-proguanil alone or in combination with artesunate. Am J Trop Med Hyg. 2005;73(2):267-8.	Wrong setting
Güris D Bayazit Y Özdemirer Ü et al. Measles epidemiology and elimination strategies in Turkey. J Infect Dis. 2003;187:S230-4.	Wrong study design
Gustafson P et al. Tuberculosis mortality during a civil war in Guinea-Bissau. JAMA : the journal of the American Medical Association. 2001;286(5):599-603.	Wrong setting
Guthmann JP Klovstad H Boccia D et al. A large outbreak of hepatitis E among a displaced population in Darfur Sudan 2004: The role of water treatment methods. Clin Infect Dis. 2006;42(12):1685-91.	Wrong study design
Haaser F. Guidance for effective elimination of cholera epidemics in a sustainable manner in the Democratic Republic of Congo and other high risk countries. Int J Infect Dis. 2014;21((Haaser) Veolia Environment Foundation Nanterre France):70.	Wrong study design
Habek D. Maternal care under minimal conditions during the war in Croatia. International Journal of Gynecology and Obstetrics. 2009;107(1):60-1.	Wrong study design
Habib M Soofi S Ali N et al. A study evaluating poliovirus antibodies and risk factors associated with polio seropositivity in low socioeconomic areas of Pakistan. Vaccine. 2013;31(15):1987-93.	Wrong topic
Hale K Wood NJ Sheikh-Mohammed M. Camp to clinic: A refugee journey. Med J Aust. 2006;185(11-12):589-90.	Wrong setting
Hale P Bouhenia M Couturier BA et al. Enteropathogens distribution and burden within oral cholera vaccine recipients in south sudan. Am J Trop Med Hyg. 2016;95(5 Supplement 1):135.	Wrong study design
Hall PF. The case against HIV antibody testing of reguees and immigrants (I). CMAJ. 1990;143(3):172.	Wrong study design
Hargreaves S Holmes A Friedland JS. Refugees asylum seekers and general practice: Room for improvement? Br J Gen Pract. 2000;50(456):531-2.	Wrong study design
Harrison KM Claass J Spiegel PB et al. HIV behavioural surveillance among refugees and surrounding host communities in Uganda 2006. Afr J AIDS Res. 2009;8(1):29-41.	Wrong study design
Hasegawa G Kyaw Y Danjuan L et al. Influenza virus infections in Yangon Myanmar. Journal of clinical virology : the official publication of the Pan American Society for Clinical Virology. 2006;37(3):233-4.	Wrong study design
Hashizume M Kondo H Murakami T et al. Use of rapid diagnostic tests for malaria in an emergency situation after the flood disaster in Mozambique. Public Health. 2006;120(5):444-7.	Wrong study design

Hatch C Sneddon J Jalloh G. A descriptive study of urban rabies during the civil war in Sierra Leone: 1995-2001. Trop Anim Health Prod. 2004;36(4):321-34.	Wrong setting
Hatch DL Waldman RJ Lungu GW et al. Epidemic cholera during refugee resettlement in Malawi. Int J Epidemiol. 1994;23(6):1292-9.	Wrong study design
Hawkes M Katsuva JP Masumbuko CK. Use and limitations of malaria rapid diagnostic testing by community health workers in war-torn Democratic Republic of Congo. Malar J. 2009;8(101139802):308.	Wrong study design
Hayani KC Pickering LK. Screening of immigrant children for infectious diseases. Adv Pediatr Infect Dis. 1991;6((Hayani Pickering) Department of Pediatrics University of Texas Medical School Houston.):91-110.	Wrong setting
Heppner Jr DG Magill AJ Gasser Jr RA et al. The threat of infectious diseases in Somalia. N Engl J Med. 1993;328(14):1061-6.	Wrong study design
Hershey CL Doocy S Anderson J et al. Incidence and risk factors for Malaria pneumonia and diarrhea in children under 5 in UNHCR refugee camps: A retrospective study. Conflict and health. 2011;5(1):24.	Wrong study design
Heudtlass P Speybroeck N Guha-Sapir D. Excess mortality in refugees internally displaced persons and resident populations in complex humanitarian emergencies (1998-2012) - insights from operational data. Conflict and health. 2016;10(101286573):15.	Wrong study design
Hewitt SE Farhan M Urhaman H et al. Self-protection from malaria vectors in Pakistan: An evaluation of popular existing methods and appropriate new techniques in Afghan refugee communities. Ann Trop Med Parasitol. 1996;90(3):337-44.	Wrong study design
Heyman SN Ginosar Y Niel L et al. Meningococcal meningitis among Rwandan refugees: Diagnosis management and outcome in a field hospital. Int J Infect Dis. 1998;2(3):137-42.	Wrong study design
Heyman SN Ginosar Y Shapiro M et al. Diarrheal epidemics among Rwandan refugees in 1994: Management and outcome in a field hospital. J Clin Gastroenterol. 1997;25(4):595-601.	Wrong study design
Heymann DL. Population movements and infectious diseases. J Med Microbiol. 1998;47(10):847-8.	Wrong study design
Hickey JE Gagnon AJ Jitthai N. Knowledge about pandemic influenza preparedness among vulnerable migrants in Thailand. Health Promot Int. 2016;31(1):124-32.	Wrong study design
Hill LL Hovell M Benenson AS. Prevention of hepatitis B transmission in indo-Chinese refugees with active and passive immunization. Am J Prev Med. 1991;7(1):29-32.	Wrong study design
Hodes RM Wolday D Kibreab T. Sensitivities of malaria in Zaire. Trop Doct. 1997;27(3):190.	Wrong study design
Holt BY Brady W Belay E et al. Planning STI/HIV prevention among refugees and mobile populations: Situation assessment of Sudanese refugees. Disasters. 2003;27(1):1-15.	Wrong study design
Houston S. Tuberculosis in refugees and displaced persons. Int J Tuberc Lung Dis. 1998;2(9 SUPPL. 1):S94-S7.	Wrong study design
Hu KK Maung C Katz DL. Clinical diagnosis of malaria on the Thai-Myanmar border. Yale J Biol Med. 2001;74(5):303-8.	Wrong study design
Huhn GD Brown J Perea W et al. Vaccination coverage survey versus administrative data in the assessment of mass yellow fever immunization in internally displaced persons - Liberia 2004. Vaccine. 2006;24(6):730-7.	Wrong study design
Huhn GD Brown J Perea W et al. Vaccination coverage survey versus administrative data in the assessment of mass yellow fever immunization in internally displaced persons--Liberia 2004. Vaccine. 2006;24(6):730-7.	Wrong study design

Meteke S, *et al. BMJ Global Health* 2020; 5:e001967. doi: 10.1136/bmjgh-2019-001967

Hukic M Hubschen JM Seremet M et al. An outbreak of rubella in the Federation of Bosnia and Herzegovina between December 2009 and May 2010 indicates failure to vaccinate during wartime (1992-1995). Epidemiol Infect. 2012;140(3):447-53.	Duplicate
Husain F Hardy C Zekele L et al. A pilot study of a portable hand washing station for recently displaced refugees during an acute emergency in Benishangul-Gumuz Regional State Ethiopia. Conflict and health. 2015;9(101286573):26.	Wrong study design
Hussain M Munir S Jamal MA et al. Epidemic outbreak of anthroponotic cutaneous leishmaniasis in Kohat District Khyber Pakhtunkhwa Pakistan. Acta Trop. 2017;172((Mohamed) UMR	Extracted in WASH
Hussain S Ali Z. Prevalence of hepatitis B virus in the Kurram Agency Pakistan: A 5-year observational study in a war-affected region. Journal of clinical virology : the official publication of the Pan American Society for Clinical Virology. 2016;82(cx0 9815671):17-9.	Wrong study design
Hussein AA Abdel Rahman SI. Meningococcal meningitis epidemic. A new role for single-dose oily chloramphenicol. Saudi Med J. 2002;23(7):797-801.	Wrong study design
Hyjazi Y Aribot J Waxman R et al. The impact of the Ebola virus epidemic on reproductive and maternal health care services in Guinea. International Journal of Gynecology and Obstetrics. 2015;131((Waxman Pleah Dao) Jhpiego	Wrong study design
Ibrahim KM Laaser U. Resistance and refugees in Pakistan: Challenges ahead in tuberculosis control. Lancet Infect Dis. 2002;2(5):270-2.	Non-English
Ibrahim MM Omar HM Persson LA et al. Child mortality in a collapsing African society. Bull World Health Organ. 1996;74(5):547-52.	Wrong study design
Iijima Y Oundo JO Taga K et al. Simultaneous outbreak due to Vibrio cholerae and Shigella dysenteriae in Kenya. Lancet (London England). 1995;345(8941):69-70.	Wrong study design
Inci A Sarici IS Caliskan G et al. Investigation of frequency of HBSAG anti HBS anti HCV and anti HIV in refugee patients from Syria who admit to a training and research hospital department of surgery. Acta Medica Mediterranea. 2017;33(1):59-63.	Wrong study design
Ingram M. Syphilis soars in Russia. BMJ (Clinical research ed). 1995;311(6997):78.	Wrong study design
Irajian GR Nassaji M Ranjbar R et al. Implementation of directly observed short course therapy for tuberculosis. J Biol Sci. 2008;8(1):217-20.	Wrong study design
Iralu JV Maguire JH. Pulmonary infections in immigrants and refugees. Semin Respir Infect. 1991;6(4):235-46.	Wrong study design
Iriso R Ocakacon R Acayo JA et al. Bacterial meningitis following introduction of Hib conjugate vaccine in northern Uganda. Ann Trop Paediatr. 2008;28(3):211-6.	Wrong setting
Isaacs D. Refugees and good Samaritans. J Paediatr Child Health. 2011;47(5):247-8.	Wrong study design
Ishiwada N Addae MM Tetteh JK et al. Vaccine-modified measles in previously immunized children in Accra Ghana: clinical virological and serological parameters. Tropical medicine & international health : TM & IH. 2001;6(9):694-8.	Wrong study design
Ismael AF El-Gilany AH. Pattern of skin diseases among central african refugees in chad. TAF Preventive Medicine Bulletin. 2015;14(4):324-8.	Wrong setting
Ismail SA Abbara A Collin SM et al. Communicable disease surveillance and control in the context of conflict and mass displacement in Syria. International journal of infectious diseases : IJID : official publication of the International Society for Infectious Diseases. 2016;47(c3r 9610933):15-22.	Wrong study design

Ito EE Egwunyenga AO. Schistosomiasis: The Aftermath of 2012 floods in delta state Southern Nigeria. International Medical Journal. 2015;22(4):218-23.	Wrong study design
Iyer AS Bouhenia M Rumunu J et al. Immune response to oral cholera vaccine (shanchol) in internally displaced persons in South Sudan. Am J Trop Med Hyg. 2016;95(5 Supplement 1):4-5.	Wrong setting
Jacoby H Rawling RA Granato PA. Cutaneous Leishmaniasis in a Central American Refugee. Clin Microbiol Newsl. 2014;36(3):22-4.	Wrong setting
Jaffer A Hotez PJ. Somalia: A Nation at the Crossroads of Extreme Poverty Conflict and Neglected Tropical Diseases. PLoS Negl Trop Dis. 2016;10(9):no pagination.	Wrong study design
Jakobsen M Sodemann M Nylen G et al. Breastfeeding status as a predictor of mortality among refugee children in an emergency situation in Guinea-Bissau. Trop Med Int Health. 2003;8(11):992-6.	Wrong study design
Jamali S. Role of pyrethroids in control of malaria amongst refugee population. J Pak Med Assoc. 2011;61(5):486-90.	Wrong study design
Jansen VAA Stollenwerk N Jensen HJ et al. Measles outbreaks in a population with declining vaccine uptake. Science (New York NY). 2003;301(5634):804.	Wrong topic
Javed S Said F Eqani SAMAS et al. Bordetella parapertussis outbreak in Bisham Pakistan in 2009-2010: Fallout of the 9/11 syndrome. Epidemiol Infect. 2015;143(12):2619-23.	Wrong setting
Jawaid A Zafar AM Mahmood SF. Impact of Afghan refugees on the infectious disease profile of Pakistan: beyond economy. Int J Infect Dis. 2008;12(6):e131-e2.	Wrong study design
Jeandron A Saidi JM Kapama A et al. Water supply interruptions and suspected cholera incidence: a time-series regression in the Democratic Republic of the Congo. PLoS Med. 2015;12(10):1-16.	Wrong study design
Jelcic D Grle M Strinic T. Respiratory infections in children hospitalized at the University Hospital Mostar during war and post-war period. Coll Antropol. 2010;34 Suppl 1((Jelcic Grle Strinic) Unit for Gynecology and Obstetrics Health Center Metkovic	Wrong study design
Jemal Y Haidar J Makau WK. The magnitude and determinants of anaemia among refugee preschool children from the Kebribeyah refugee camp Somali region Ethiopia. South Afr J Clin Nutr. 2017;30(1):17-23.	Wrong study design
Jensen P Hovig B. [Lupus vulgaris. Cutaneous tuberculosis in a Vietnamese refugee]. Tidsskrift for den Norske laegeforening : tidsskrift for praktisk medicin ny raekke. 1993;113(25):3136-7.	Wrong study design
Joshi AB Luman ET Nandy R et al. Measles deaths in Nepal: estimating the national case-fatality ratio. Bull World Health Organ. 2009;87(6):456-65.	Wrong setting
Kaadan A. The effect of civil war on cutaneous leishmaniasis "aleppo button" in aleppo city. Am J Trop Med Hyg. 2015;93(4 Supplement):128-9.	Wrong study design
Kaadan A. The effect of civil war on cutaneous leishmaniasis ("aleppo button") in aleppo city syria. Am J Trop Med Hyg. 2014;91(5 SUPPL. 1):330.	Duplicate
Kakar RM Mojadidi MK Mofleh J. Pertussis in afghanistan 2007-2008. Emerg Infect Dis. 2009;15(3):501-.	Wrong study design
Kalipeni E Oppong J. The refugee crisis in Africa and implications for health and disease: A political ecology approach. Soc Sci Med. 1998;46(12):1637-53.	Wrong study design
Kalisya LM Salmon M Manwa K et al. The state of emergency care in Democratic Republic of Congo. African Journal of Emergency Medicine. 2015;5(4):153-8.	Wrong study design
Kamath SS. Child protection during disasters. Indian Pediatr. 2015;52(6):467-8.	Wrong study design

Kamugisha C Cairns KL Akim C. An outbreak of measles in Tanzanian refugee camps. J Infect Dis. 2003;187(SUPPL. 1):S58-S62.	Wrong study design
Kandala NB Mandungu TP Mbela K et al. Child mortality in the Democratic Republic of Congo: cross-sectional evidence of the effect of geographic location and prolonged conflict from a national household survey. BMC Public Health. 2014;14((Kandala Mandungu Mbela	Wrong study design
Kaninda AV Legros D Jataou IM et al. Measles vaccine effectiveness in standard and early immunization strategies Niger 1995. The Pediatric infectious disease journal. 1998;17(11):1034-9.	Wrong study design
Kaplan EL. Rheumatic fever. Curr Opin Rheumatol. 1990;2(5):836-8.	Wrong study design
Kapp C. Zimbabwe's humanitarian crisis worsens. Lancet. 2009;373(9662):447.	Wrong study design
Karim AM Hussain I Malik SK et al. Epidemiology and Clinical Burden of Malaria in the War-Torn Area Orakzai Agency in Pakistan. PLoS Negl Trop Dis. 2016;10(1):no pagination.	Wrong study design
Karkee R Shrestha DB. HIV and conflict in Nepal: Relation and strategy for response. Kathmandu University Medical Journal. 2006;4 NO. 3(15):363-7.	Wrong study design
Karki A Tiwari BR. Prevalence of acute diarrhoea in Kathmandu valley. JNMA; journal of the Nepal Medical Association. 2007;46(168):175-9.	Wrong study design
Karsany MS Elshayeb AA Saeed ES et al. Patterns of meningococcal infection in Sudan with emergence of Neisseria meningitidis serogroup W135. Eastern Mediterranean Health Journal. 2013;19(10):843-6.	Wrong study design
Katungi A Redeker S Kiiza P et al. REASSESSMENT OF HELMINTH INFECTIONS IN GULU MUNICIPALITY NOTHERN UGANDA AFTER THE TWENTY YEARS OF INSURGENCY: USING THREE DIAGONOSTIC METHODS TO COMPARE THEIR SENSITIVITY. East Afr Med J. 2013;90(3):95-103.	Wrong study design
Kazmi JH Pandit K. Disease and dislocation: The impact of refugee movements on the geography of malaria in NWFP Pakistan. Soc Sci Med. 2001;52(7):1043-55.	Wrong study design
Kelly M. Breastfeeding in emergencies. Dialogue on diarrhoea. 1995(59):7.	Wrong study design
Kemp C Roberts A. Infectious diseases of refugees and immigrants. Viral hemorrhagic fevers. J Am Acad Nurse Pract. 2002;14(4):146-9.	Wrong study design
Kennedy M Van Houten C. Providing AIDS related services to recently arrived immigrant and refugee youth. AIDS education and prevention : official publication of the International Society for AIDS Education. 1992;Suppl((Kennedy Van Houten) Larkin Street Youth Center San Francisco CA.):83-6.	Wrong study design
Kessler C Connolly M Levy M et al. Tuberculosis control in refugees and displaced persons. Revista Panamericana de Salud Publica/Pan American Journal of Public Health. 1997;2(4):295-8.	Non-English
Keus K Houston S Melaku Y et al. Treatment of a cohort of tuberculosis patients using the Manyatta regimen in a conflict zone in South Sudan. Trans R Soc Trop Med Hyg. 2003;97(6):614-8.	Wrong study design
Khan F Akbar H Idrees M et al. The prevalence of HBV infection in the cohort of IDPs of war against terrorism in Malakand Division of Northern Pakistan. BMC Infect Dis. 2011;11((Akbar Shahzad) Department of Animal Sciences University of Illinoise Urbana Champaign	Wrong study design
Khan IM Laaser U. Burden of tuberculosis in Afghanistan: Update on a war-stricken country. Croat Med J. 2002;43(2):245-7.	Wrong study design
Khan MI Ochiai RL Hamzal HB et al. Lessons and implications from a mass immunization campaign in squatter settlements of Karachi Pakistan: An experience from a cluster-randomized double-blinded vaccine trial [NCT00125047]. Trials. 2006;7((Donner) University of Western Ontario London	Wrong setting

Khan MI Soofi SB Ochiai RL et al. Effectiveness of Vi capsular polysaccharide typhoid vaccine among children: A cluster randomized trial in Karachi Pakistan. Vaccine. 2012;30(36):5389-95.	Wrong setting
Khan S Hesketh T. Deteriorating situation for street children in Pakistan: A consequence of war. Arch Dis Child. 2010;95(8):655-7.	Wrong study design
Khan WA. Single-dose azithromycin for childhood cholera. Indian Pediatr. 2010;47(4):305-6.	Wrong study design
Khanani MR Ansari AS Khan S et al. Concentrated epidemics of HIV HCV and HBV among Afghan refugees. J Infect. 2010;61(5):434-7.	Wrong study design
Khazaei S Salehiniya H Pakzad R et al. The status of Iran measles surveillance system in 2014. Journal of Isfahan Medical School. 2015;33(349):no pagination.	Non-English
Khetsuriani N Imnadze P Dekanosidze N. Diphtheria epidemic in the Republic of Georgia 1993-1997. J Infect Dis. 2000;181(SUPPL. 1):S80-S5.	Wrong setting
Khoury S Graczyk T Burnham G et al. Drinking water system treatment and contamination in shatila refugee camp in Beirut Lebanon. Eastern Mediterranean Health Journal. 2016;22(8):568-78.	Wrong topic
Khuri-Bulos NA. Measles in Jordan: a prototype of the problems with measles in developing countries. The Pediatric infectious disease journal. 1995;14(1):22-6.	Wrong study design
Kiang K. Predicted increase in need for comprehensive refugee/migrant health services as climate change provokes further population displacement. J Paediatr Child Health. 2013;49(2):159-60.	Wrong study design
Kibadi K Tsakala M Mputu-Yamba J-B et al. [Buruli ulcer in Angolese refugees in the Kimpese area Lower Congo D.R. Congo]. Sante (Montrouge	Wrong study design
Kibadi K Tsakala M Mputu-Yamba JB et al. Buruli ulcer in Angolese refugees in the Kimpese area Lauer Congo D.R. Congo. Cahiers Sante. 2003;13(1):39-41.	Duplicate
Kiboneka A Nyatia RJ Nabiryo C et al. Pediatric HIV therapy in armed conflict. AIDS. 2008;22(9):1097-8.	Wrong study design
Kiemanh P Chambers Sharpe E Weiss WM et al. The use of a lot quality assurance sampling methodology to assess and manage primary health interventions in conflict-affected West Darfur Sudan. Population Health Metrics. 2016;14:1-12.	Wrong study design
Kim C Nyoka R Ahmed JA et al. Epidemiology of respiratory infections caused by atypical bacteria in two Kenyan refugee camps. Journal of immigrant and minority health / Center for Minority Public Health. 2012;14(1):140-5.	Wrong study design
Kirkbya K Galappaththy GNL Kurinczuk JJ et al. Knowledge attitudes and practices relevant to malaria elimination amongst resettled populations in a post-conflict district of northern Sri Lanka. Trans R Soc Trop Med Hyg. 2013;107(2):110-8.	Wrong study design
Kis D. Transylvanian refugees and the plague in 1708-1709. Orvostorteneti kozlemenyek. 1993;39(1-4):83-105.	Wrong study design
Kitara DL Amone C Okello C. Knowledge and misconceptions about HIV counseling and testing (HCT) among the post-conflict youths of Gulu Northern Uganda. A prospective study design. The Pan African medical journal. 2012;12((Kitara) Gulu University Faculty of Medicine Gulu	Wrong study design
Kolaczinski J Brooker S Reyburn H et al. Epidemiology of anthroponotic cutaneous leishmaniasis in Afghan refugee camps in northwest Pakistan. Trans R Soc Trop Med Hyg. 2004;98(6):373-8.	Wrong study design
Kolaczinski J. Roll Back Malaria in the aftermath of complex emergencies: The example of Afghanistan. Trop Med Int Health. 2005;10(9):888-93.	Wrong setting

Kolaczinski JH Muhammad N Khan QS et al. Subsidized sales of insecticide-treated nets in Afghan refugee camps demonstrate the feasibility of a transition from humanitarian aid towards sustainability. Malar J. 2004;3((Rehman) Diplomatic Enclave-2 Quaid-e-Azam University Road	Wrong study design
Kolaczinski JH Ojok N Opwonya J et al. Adherence of community caretakers of children to pre-packaged antimalarial medicines (HOMAPAK) among internally displaced people in Gulu district Uganda. Malar J. 2006;5((Meek) Malaria Consortium Head Office	Wrong study design
Kondaj R. Management of refugee crisis in Albania during the 1999 Kosovo conflict. Croat Med J. 2002;43(2):190-4.	Wrong study design
Kopel E Amitai Z Sprecher H et al. Tinea capitis outbreak in a paediatric refugee population Tel Aviv Israel. Mycoses. 2012;55(2):e36-e9.	Wrong setting
Kose S Odemis I Celik D et al. Hepatitis A B C and HIV seroprevalence among syrian refugee children admitted to outpatient clinics. Infezioni in Medicina. 2017;25(4):339-43.	Wrong study design
Krause SK Jones RK Purdin SJ. Programmatic responses to refugees' reproductive health needs. Int Fam Plan Perspect. 2000;26(4):181-7.	Wrong topic
Krause SK Otieno M Lee C. Reproductive health for refugees. Lancet. 2002;360(SUPPL. 1):s15-s6.	Wrong topic
Krcmery V. Infections in the disaster setting: Famine. Experience from Darfour Sudan. Clin Microbiol Infect. 2009;15((Krcmery) BratislavaSlovakia):S3.	Wrong topic
Krishna BVS Patil AB Chandrasekhar MR. Fluoroquinolone-resistant Vibrio cholerae isolated during a cholera outbreak in India. Trans R Soc Trop Med Hyg. 2006;100(3):224-6.	Wrong setting
Kulstrunk M Evequoz D Dubach VC et al. Prevalence of hepatitis B virus in Kurdish refugees. J Hepatol. 1992;15(3):418-9.	Wrong setting
Kyawt Kyawt S Pearson A. Knowledge attitudes and practices with regard to malaria control in an endemic rural area of Myanmar. The Southeast Asian journal of tropical medicine and public health. 2004;35(1):53-62.	Wrong study design
Kyronseppa H. Health care of refugees. Duodecim; laaketieteellinen aikakauskirja. 1992;108(1):13-5.	Non-English
Lafta R Aflouk NA Dhiaa S et al. Needs of internally displaced women and children in Baghdad Karbala and Kirkuk	Wrong study design
Laifer G Widmer AF Frei R et al. Polymerase chain reaction for Mycobacterium tuberculosis: Impact on clinical management of refugees with pulmonary infiltrates. Chest. 2004;125(3):981-6.	Wrong setting
Lainez YB Todd CS Ahmadzai A et al. Prevalence of respiratory symptoms and cases suspicious for tuberculosis among public health clinic patients in Afghanistan 2005-2006: Perspectives on recognition and referral of tuberculosis cases. Trop Med Int Health. 2009;14(5):564-70.	Wrong study design
Lal S Mertens TE. HIV and AIDS. Indian J Public Health. 1995;39(3):77-8.	Wrong study design
Lam E McCarthy A Brennan M. Vaccine-preventable diseases in humanitarian emergencies among refugee and internally-displaced populations. Human Vaccines and Immunotherapeutics. 2015;11(11):2627-36.	Wrong study design
Lamour P Bouree P Hennequin C et al. [Blind treatment or treatment oriented to intestinal parasitoses in a Parisian health center for refugees]. Sante (Montrouge France). 1994;4(1):21-6.	Wrong setting
Lamour P Bouree P Hennequin C et al. Diagnostic screening evaluation for parasitic infections in a health center for refugees. Cahiers Sante. 1994;4(1):21-6.	Wrong setting
Landazabal Garcia N Burgos Rodriguez MM Pastor D. Diphtheria outbreak in Cali Colombia August-October 2000. Epidemiol Bull. 2001;22(3):13-5.	Wrong setting

Lawn JE Reef S Baffoe-Bonnie B et al. Unseen blindness unheard deafness and unrecorded death and disability: congenital rubella in Kumasi	Wrong study design
Leblebicioglu H Ozaras R. Syrian refugees and infectious disease challenges. Travel Med Infect Dis. 2015;13(6):443-4.	Wrong study design
Lee CI Smith LS Shwe Oo EK et al. Internally displaced human resources for health: villager health worker partnerships to scale up a malaria control programme in active conflict areas of eastern Burma. Global Public Health. 2009;4(3):229-41.	Wrong study design
Lee MS King CC Chen CJ et al. Epidemiology of measles in Taiwan: Dynamics of transmission and timeliness of reporting during an epidemic in 1988-9. Epidemiol Infect. 1995;114(2):345-59.	Wrong study design
Lee TJ Mullany LC Richards AK et al. Mortality rates in conflict zones in Karen Karenni and Mon states in eastern Burma. Trop Med Int Health. 2006;11(7):1119-27.	Wrong study design
Lengeler C Kessler W Daugla D. The 1990 meningococcal meningitis epidemic of Sarh (Chad): how useful was an earlier mass vaccination? Acta Trop. 1995;59(3):211-22.	Wrong study design
Lepow ML. Measles vaccine and measles control. Pediatr Ann. 1990;19(9):543-50.	Wrong setting
Leslie T Briceno M Mayan I et al. The impact of phenotypic and genotypic G6PD deficiency on risk of Plasmodium vivax infection: A case-control study amongst Afghan refugees in Pakistan. PLoS Med. 2010;7(5):no pagination.	Wrong study design
Lienhardt C Ghebray R Candolfi E et al. Malaria in refugee camps in eastern Sudan: A sero-epidemiological approach. Ann Trop Med Parasitol. 1990;84(3):215-22.	Wrong study design
Little M Hodge JV. Operation habitat: Humanitarian aid to the Kurdish refugees in northern Iraq. Med J Aust. 1991;155(11-12):807-12.	Wrong study design
Litvinjenko S. Migration and health. Srp Arh Celok Lek. 1997;125(7-8):191-6.	Non-English
Liu Y Posey DL Cetron MS et al. Tuberculosis Incidence in Immigrants and Refugees. In Response. Philadelphia Pennsylvania: American College of Physicians; 2015. p. 150-1.	Wrong setting
Loncarevic N Dzelinovic J Alajbegovic A et al. Acute polyradiculoneuritis--clinical course and outcome during the war and postwar period. Med Arh. 2004;58(4):218-9.	Wrong topic
London L Zweigenthal V. Malaria among exiles returning to South Africa [13]. S Afr Med J. 1993;83(9):694-5.	Wrong study design
Longini IM Jr. Nizam A Ali M et al. Controlling endemic cholera with oral vaccines. PLoS Med. 2007;4(11):e336.	Wrong study design
Longini IM Jr. Nizam A Xu S et al. Containing pandemic influenza at the source. Science (New York NY). 2005;309(5737):1083-7.	Wrong study design
López Torres Z Ochoa Marín SC López GA et al. Sexually transmitted diseases and AIDS vulnerability in women in forced displacement situation. Medellin Columbia. Investigacion & Educacion en Enfermeria. 2010;28(1):11-22.	Wrong setting
Lopez-Gonzalez JM Tuells J. Vaccinology in armed conflicts: A punctual intervention in poliomyelitis eradication in Afghanistan. Gac Sanit. 2006;20(3):244-7.	Wrong setting
Lucas M Nicol P McKinnon E et al. A prospective large-scale study of methods for the detection of latent Mycobacterium tuberculosis infection in refugee children. Thorax. 2010;65(5):442-8.	Non-English
Lucas SE. AIDS: Refugees and the homeless. AIDS Care - Psychological and Socio-Medical Aspects of AIDS/HIV. 1991;3(4):443-6.	Wrong study design

Lurio J Verson H Karp S. Intestinal parasites in Cambodians: Comparison of diagnostic methods used in screening refugees with implications for treatment of populations with high rates of infestation. J Am Board Fam Pract. 1991;4(2):71-8.	Wrong setting
Luthi JC Kessler W Boelaert M. [A survey on vaccine efficacy in the city of Bongor (Chad) and its operational consequences for the vaccination program]. Bull World Health Organ. 1997;75(5):427-33.	Non-English
Luxemburger C Rigal J Nosten F. Health care in refugee camps. Trans R Soc Trop Med Hyg. 1998;92(2):129-30.	Wrong study design
Luxemburger C White NJ ter Kuile F et al. Beri-beri: The major cause of infant mortality in Karen refugees. Trans R Soc Trop Med Hyg. 2003;97(2):251-5.	Wrong study design
Ma C Claude KM Kibendelwa ZT et al. Is maternal education a social vaccine for childhood malaria infection? A cross-sectional study from war-torn Democratic Republic of Congo. Pathogens and global health. 2017;111(2):98-106.	Wrong study design
Maalim AM Zachariah R Khogali M et al. Supporting 'medicine at a distance' for delivery of hospital services in war-torn somalia: How well are we doing? International Health. 2014;6(1):70-3.	Wrong study design
Maayan S Marks N Viterbro A et al. HIV infection and susceptibility to epidemic bacterial infections among Rwandan refugees. Int J Infect Dis. 1997;1(4):199-201.	Wrong study design
Macassa G Ghilagaber G Bernhardt E et al. Trends in infant and child mortality in Mozambique during and after a period of conflict. Public Health. 2003;117(4):221-7.	Wrong study design
Mach A. Congo polio immunisation campaign gets go ahead. BMJ (Clinical research ed). 1999;318(7186):756.	Wrong study design
Maganga GD Kapetshi J Berthet N et al. Ebola virus disease in the Democratic Republic of Congo. N Engl J Med. 2014;371(22):2083-91.	Wrong study design
Mahalanabis D Choudhuri AB Bagchi NG et al. Oral fluid therapy of cholera among Bangladesh refugees. Bull World Health Organ. 2001;79(5):473-9.	Wrong setting
Mahamud A Ahmed J Nyoka R et al. Epidemic cholera in kakuma refugee camp kenya: the importance of sanitation and soap. Am J Trop Med Hyg. 2010;83(5 SUPPL. 1):191.	Wrong study design
Mahamud A Burton A Hassan M et al. Risk factors for measles mortality among hospitalized Somali refugees displaced by famine Kenya 2011. Clin Infect Dis. 2013;57(8):e160-e6.	Wrong study design
Mahamud A Kamadjeu R Webeck J et al. Effectiveness of oral polio vaccination against paralytic poliomyelitis: a matched case-control study in Somalia. J Infect Dis. 2014;210(suppl_1):S187-93.	Wrong study design
Mahmood J. Women's health in crisis situations. International Journal of Gynecology and Obstetrics. 2012;119:S217.	Wrong study design
Mahmoud EA Sheikh AH Domeika MA et al. Prevalence of trachoma among displaced persons in The Sudan: A clinical and sero-epidemiological study. Eye. 1994;8(1):130-3.	Wrong study design
Mala P Ghada M Wasan A et al. Establishment of EWARN system for the Syrian crisis: Experiences and challenges. Int J Infect Dis. 2014;21((Mansour Rady) World Health Organization Beirut	Wrong study design
Malamba S Muyinda H Blair AH et al. "The Cango lyec project-healing the elephant": Risk factors for HIV infection among post conflict populations in Northern Uganda. Canadian Journal of Infectious Diseases and Medical Microbiology. 2014;25((Patel) OttawaONCanada):22A-3A.	Wrong study design
Mamova A Balazova M Dibusova L et al. Severe malaria among 3707 admissions in south Sudanese hospital for internally displaced population. Am J Trop Med Hyg. 2014;91(5 SUPPL. 1):463.	Wrong study design

Mancini S Coldiron ME Nicholas S et al. Physiotherapy for poliomyelitis: a descriptive study in the Republic of Congo. BMC Res Notes. 2014;7((Mancini Coldiron Nicholas	Wrong setting
Mangoud AM. Effect of parasite screening on refugee health. J Egypt Soc Parasitol. 2000;30(1):1-10.	Wrong study design
mariam MW Gelaw B Assefa A. Seroprevalence of typhus fever at the Kality Prison Addis Ababa Ethiopia. Biomedical Research and Therapy. 2015;2(7):no pagination.	Wrong patient populati
Marlet MVL Wuillaume F Jacquet D et al. A neglected disease of humans: A new focus of visceral leishmaniasis in Bakool Somalia. Trans R Soc Trop Med Hyg. 2003;97(6):667-71.	Wrong study design
Marnell F Guillet A Holland C. A survey of the intestinal helminths of refugees in Juba Sudan. Ann Trop Med Parasitol. 1992;86(4):387-93.	Wrong study design
Marshall R Barkess-Jones L Sivayoham S. An outbreak of scabies in a school for children with learning disabilities. Commun Dis Rep CDR Rev. 1995;5(6):R90-2.	Wrong study design
Martin Diaz MJ Espejo Moreno L Hernandez Caballero MJ et al. Measles outbreak in Saharan refugee camps [4]. Anales de Pediatria. 2004;60(5):483-4.	Non-English
Martins N et al. Tuberculosis control in conflict-affected East Timor 1996-2004. Int J Tuberc Lung Dis. 2006;10(9):975-81.	Wrong study design
Martins N Kelly PM Grace JA et al. Reconstructing tuberculosis services after major conflict: experiences and lessons learned in East Timor. PLoS Med. 2006;3(10):e383.	Wrong study design
Mateen FJ Carone M Al-Saedy H et al. Medical conditions among Iraqi refugees in Jordan: Data from the United Nations refugee assistance information system. Bull World Health Organ. 2012;90(6):444-51.	Wrong study design
Mauch V et al. Structure and management of tuberculosis control programs in fragile states--Afghanistan DR Congo Haiti Somalia. Health Policy. 2010;96(2):118-27.	Wrong study design
Mbaeyi C Kamadjeu R Mahamud A et al. Progress toward polio eradication--Somalia 1998-2013. J Infect Dis. 2014;210(suppl_1):S173-80.	Wrong study design
Mbaeyi C Saatcioglu A Tangermann RH et al. Progress towards poliomyelitis eradication: Afghanistan January 2014-August 2015. Wkly Epidemiol Rec. 2015;90(43):581-8.	Wrong study design
M'Boussa J Yokolo D Pereira B et al. A flare-up of tuberculosis due to war in Congo Brazzaville. Int J Tuberc Lung Dis. 2002;6(6):475-8.	Wrong study design
McCarthy AE Weld LH Barnett ED et al. Spectrum of illness in international migrants seen at geosentinel clinics in 1997-2009 part 2: Migrants resettled internationally and evaluated for specific health concerns. Clin Infect Dis. 2013;56(7):925-33.	Wrong study design
McCleery EJ Patchanee P Pongsopawijit P et al. Taeniasis among Refugees Living on Thailand-Myanmar Border 2012. Emerg Infect Dis. 2015;21(10):1824-6.	Wrong study design
McFee RB. Adolescent health and terrorism - The role of the adolescent medicine specialist. J Adolesc Health. 2002;30(5):300-1.	Wrong study design
McGready R Ashley EA Wuthiekanun V et al. Arthropod borne disease: The leading cause of fever in pregnancy on the thai-burmese border. PLoS Negl Trop Dis. 2010;4(11):no pagination.	Wrong study design
McGready R. Artemisinin derivatives in the treatment of falciparum malaria in pregnancy. Trans R Soc Trop Med Hyg. 1998;92(4):430-3.	Wrong study design
Medlock JM Aryemo M Bean J. Impact of mosquito proofing of night shelters in refugee camps in Kitgum northern Uganda. Trop Med Int Health. 2007;12(3):370-6.	Wrong study design
Meek S Rowland M. Malaria in emergency situations. World Health. 1998;51(3):22-3.	Wrong study design

Meijman HJ Blok ML Griekspoor A. 'Medecins sans Frontieres' and diarrhea as cause of disease and mortality in refugee camps. Ned Tijdschr Geneeskd. 1996;140(32):1658-62.	Non-English
Meiqari L Hoetjes M Baxter L et al. Impact of war on child health in northern Syria: the experience of Medecins Sans Frontieres. Eur J Pediatr. 2018;177(3):371-80.	Wrong study design
Mendelsohn JB Schilperoord M Spiegel P et al. Poor treatment outcomes among both refugees and host community accessing highly active antiretroviral therapy (HAART) from Kakuma refugee camp in northwestern Kenya. J Int AIDS Soc. 2012;15((Ross) London School of Hygiene and Tropical Medicine MRC Tropical Epidemiology Group Department of Infectious Disease Epidemiology	Wrong study design
Mendelsohn JB Spiegel P Schilperoord M et al. Acceptable adherence and treatment outcomes among refugees and host community on highly active antiretroviral therapy (HAART) in an urban refugee setting in Kuala Lumpur Malaysia. J Int AIDS Soc. 2012;15((Grant) London School of Hygiene and Tropical Medicine Department of Clinical Research	Wrong study design
Mendelsohn JB Spiegel P Schilperoord M et al. Antiretroviral Therapy for Refugees and Internally Displaced Persons: A Call for Equity. PLoS Med. 2014;11(6):1-6.	Wrong study design
Mendelsohn JB Spiegel P Schilperoord M et al. Managing antitretroviral therapy in urban and camp refugee settings: Challenges in monitoring adherence and virologic outcomes. Am J Trop Med Hyg. 2013;89(5 SUPPL. 1):22.	Wrong topic
Merlin M Martet G Debonne JM et al. [Control of an epidemic of meningococcal meningitis in Central Africa]. Sante (Montrouge France). 1996;6(2):87-95.	Non-English
Meyer H Perrichot M Stemmler M et al. Outbreaks of disease suspected of being due to human monkeypox virus infection in the Democratic Republic of Congo in 2001. J Clin Microbiol. 2002;40(8):2919-21.	Wrong setting
Milleliri JM Soares JL Bunzele G et al. Collective alimentary toxic infection in a camp for non-accompanied refugee children in the city of Goma Zaire September 1994. Cahiers Sante. 1995;5(4):253-7.	Non-English
Milleliri JM Soares JL Signoret J et al. Epidemic of bacillary dysentery in the Rwanda refugee camps of the Goma region (Zaire North Kivu) in August 1994. Ann Soc Belg Med Trop. 1995;75(3):201-10.	Non-English
Minetti A Bopp C Fermon F et al. Measles outbreak response immunization is context-specific: insight from the recent experience of Medecins Sans Frontieres. PLoS Med. 2013;10(11):e1001544.	Duplicate
Minetti A Bopp C Fermon F et al. Measles outbreak response immunization is context-specific: insight from the recent experience of médecins sans frontières. PLoS Med. 2013;10(11):e1001544-e.	Wrong study design
Mintz M Boland M O'Hara MJ et al. Pediatric HIV infection in Elista Russia: interventional strategies. Am J Public Health. 1995;85(4):586-8.	Wrong study design
Mishra A Mishra S Jain P et al. Measles related complications and the role of vitamin A supplementation. Indian J Pediatr. 2008;75(9):887-90.	Wrong setting
Mishra AK Gorbacheva O Hasan MMT et al. Varicella (Chickenpox) outbreak in Bhutanese refugee camps in Eastern Nepal. Int J Infect Dis. 2010;14((Rimal) Association of Medical Doctors of Asia (AMDA) Jhapa Nepal):e134.	Wrong study design
Mitchell T Dalal W Klosovsky A et al. An expanded immunization program for US-bound refugees: Ethiopia Kenya Malaysia	Wrong setting

Mittal PK Sreehari U Razdan RK et al. Evaluation of the impact of zerofly an insecticide incorporated plastic sheeting on malaria incidence in two temporary labour shelters in India. J Vector Borne Dis. 2011;48(3):138-43.	Wrong setting
Mohamed GA Ahmed JA Marano N et al. Etiology and incidence of viral Acute Respiratory infections among refugees aged 5 years and older in Hagadera Camp Dadaab Kenya. Am J Trop Med Hyg. 2015;93(6):1371-6.	Wrong setting
Mohammed I Nasidi A Alkali AS et al. A severe epidemic of meningococcal meningitis in Nigeria 1996. Trans R Soc Trop Med Hyg. 2000;94(3):265-70.	Wrong study design
Mohan A Murhekar MV Wairgkar NS et al. Measles transmission following the tsunami in a population with a high one-dose vaccination coverage Tamil Nadu India 2004-2005. BMC Infect Dis. 2006;6((Wairgkar) National Institute of Virology (ICMR)	Wrong study design
Moons P Thallinger M. High incidence of subcutaneous emphysema in children in a somali refugee camp during measles outbreak. Pediatr Infect Dis J. 2014;33(1):96-8.	Wrong study design
Moore PS Marfin AA Quenemoen LE et al. Mortality rates in displaced and resident populations of central Somalia during 1992 famine. Lancet. 1993;341(8850):935-8.	Wrong study design
Moore PS Toole MJ Nieburg P et al. Surveillance and control of meningococcal meningitis epidemics in refugee populations. Bull World Health Organ. 1990;68(5):587-96.	Wrong study design
Moore-Gillon J. Issues facing TB control (2.2) - TB control in refugee populations. Scott Med J. 2000;45(5 SUPPL.):29.	Wrong study design
Moren A Stefanaggi S Antona D et al. Practical field epidemiology to investigate a cholera outbreak in a Mozambican refugee camp in Malawi 1988. J Trop Med Hyg. 1991;94(1):1-7.	Wrong study design
Moreno A Crosby S Labelle C et al. Health assessment of HIV-infected refugees [6]. J Acquir Immune Defic Syndr. 2003;34(2):251-4.	Wrong setting
Moreton J. MMR vaccination: protecting our children. The journal of family health care. 2002;12(2):31.	Wrong setting
Morikawa M. Upper respiratory infection in acute pediatric care in internal conflict Kosovo 1999. J Trop Pediatr. 2001;47(6):379-82.	Wrong study design
Moss N Stone MC Smith JB. Child health outcomes among Central American refugees and immigrants in Belize. Soc Sci Med. 1992;34(2):161-7.	Wrong study design
Moszynski P. Bird flu adds to Sudans woes as UN warns of risk to aid operations. BMJ (Clinical research ed). 2006;332(7548):993.	Wrong study design
Moszynski P. Cholera outbreak in Darfur is made worse by advent of rains. BMJ (Clinical research ed). 2006;332(7556):1472.	Wrong study design
Moszynski P. Medicine and conflict: Humanitarianism on trial in Sudan. BMJ (Online). 2009;338(7700):916-7.	Wrong study design
Mughal A Kazi YF Bukhari HA et al. Pertussis resurgence among vaccinated children in Khairpur Sindh Pakistan. Public Health (Elsevier). 2012;126(6):518-22.	Wrong study design
Mugoya I Kariuki S Galgalo T et al. Rapid spread of Vibrio cholerae O1 throughout Kenya 2005. Am J Trop Med Hyg. 2008;78(3):527-33.	Wrong study design
Muhjazi G Bashour H Abourshaid N et al. An early warning and response system for Syria. The Lancet. 2013;382(9910):2066.	Wrong study design

Mullany LC Lee TJ Yone L et al. Impact of community-based maternal health workers on coverage of essential maternal health interventions among internally displaced communities in eastern Burma: The MOM project. PLoS Med. 2010;7(8):no pagination.	Wrong topic
Mullany LC Richards AK Lee CI et al. Population-based survey methods to quantify associations between human rights violations and health outcomes among internally displaced persons in eastern Burma. J Epidemiol Community Health. 2007;61(10):908-14.	Wrong study design
Muller SM Gysin S Schweitzer M et al. An algorithm for the management of scabies mass outbreaks. Exp Dermatol. 2018;27(3):e20.	Wrong setting
Murray J McFarland DA Waldman RJ. Cost-effectiveness of oral cholera vaccine in a stable refugee population at risk for epidemic cholera and in a population with endemic cholera. Bull World Health Organ. 1998;76(4):343-52.	Wrong topic
Murray M Rasmussen Z. Measles outbreak in a northern Pakistani village: epidemiology and vaccine effectiveness. Am J Epidemiol. 2000;151(8):811-9.	Wrong study design
Murshidi MM Hijjawi MQB Jeriesat S et al. Syrian refugees and Jordan's health sector. The Lancet. 2013;382(9888):206-7.	Wrong study design
Muyinda H Malamba S Tebere L et al. "The cango lyec project-healing the elephant-": Women at risk-differences in HIV risk factors between men and women in post conflict Northern Uganda. Canadian Journal of Infectious Diseases and Medical Microbiology. 2014;25((Patel) OttawaONCanada):30A.	Wrong study design
Mworozi EA. AIDS and civil war: a devil's alliance. Dislocation caused by civil strife in Africa provides fertile ground for the spread of HIV. AIDS Anal Afr. 1993;3(6):8-10.	Wrong study design
Nabeth P Vasset B Guerin P et al. Health situation of refugees in Eastern Zaire [14]. Lancet. 1997;349(9057):1031-2.	Wrong study design
Nacher M Carrara VI Ashley E et al. Seasonal variation in hyperparasitaemia and gametocyte carriage in patients with Plasmodiom falciparum malaria on the Thai - Burmese border. Trans R Soc Trop Med Hyg. 2004;98(5):322-8.	Wrong study design
Naficy A Rao MR Paquet C et al. Treatment and vaccination strategies to control cholera in sub-Saharan refugee settings: A cost-effectiveness analysis. J Am Med Assoc. 1998;279(7):521-5.	Wrong study design
Naidoo A Patric K. Cholera: A continuous epidemic in Africa. Journal of The Royal Society for the Promotion of Health. 2002;122(2):89-94.	Wrong study design
Ndihokubwayo JB Nyongabo T Nkurikiye S et al. [A new epidemic of meningococcal meningitis in Burundi]. Medecine tropicale : revue du Corps de sante colonial. 1997;57(1):98-9.	Non-English
Ndihokubwayo JB Nyongabo T Nkurikiye S et al. A new epidemic of meningococcal meningitis in Burundi. Medecine tropicale : revue du Corps de sante colonial. 1997;57(1):98-9.	Non-English
Ndikuyeze A Cook A Cutts FT et al. Priorities in global measles control: report of an outbreak in N'Djamena Chad. Epidemiol Infect. 1995;115(2):309-14.	Wrong study design
Ndongosieme A et al. Collaboration between a TB control programme and NGOs during humanitarian crisis:Democratic Republic of the Congo. Bull World Health Organ. 2007;85(8):642-3.	Wrong study design
Ndounga M Casimiro PN Miakassissa-Mpassi V et al. [Malaria in health centres in the southern districts of Brazzaville Congo]. Bulletin de la Societe de pathologie exotique (1990). 2008;101(4):329-35.	Non-English
Nelson LJ Naik Y Tsering K et al. Population-based risk factors for tuberculosis and adverse outcomes among Tibetan refugees in India 1994-1996. Int J Tuberc Lung Dis. 2005;9(9):1018-26.	Wrong study design

Nemsadze K Beleshadze M Beitrishvili L et al. Ensuring timely and continued breastfeeding in emergency in Georgia. Breastfeed Med. 2009;4(4):236.	Wrong topic
Ngondi J Ole-Sempele F Onsarigo A et al. Blinding trachoma in postconflict southern Sudan. PLoS Med. 2006;3(12):2424-30.	Wrong study design
N'Goran AA Ilunga N Coldiron ME et al. Community-based measles mortality surveillance in two districts of Katanga Province Democratic Republic of Congo. BMC Res Notes. 2013;6(101462768):537.	Wrong study design
Niel L Lamarque D Coue JC et al. [Chronical of a declared meningococcal meningitis epidemic (Goma Zaire August 1994)]. Bulletin de la Societe de pathologie exotique (1990). 1997;90(5):299-302.	Non-English
Nigatu W Nokes DJ Afework A et al. Serological and molecular epidemiology of measles virus outbreaks reported in Ethiopia during 2000-2004. J Med Virol. 2006;78(12):1648-55.	Wrong patient populati
Nimri LF Hijazi S. Rotavirus-associated diarrhoea in children in a refugee camp in Jordan. J Diarrhoeal Dis Res. 1996;14(1):1-4.	Wrong study design
Nkoghe D Kone ML Yada A et al. A limited outbreak of Ebola haemorrhagic fever in Etoumbi Republic of Congo 2005. Trans R Soc Trop Med Hyg. 2011;105(8):466-72.	Wrong setting
Noden BH Pearson RJC Gomes A. Age-specific mortality patterns in Central Mozambique during and after the end of the Civil War. Conflict and health. 2011;5(1):8.	Wrong study design
Nogodalla MA. Yellow fever outbreak investigation and response Darfur State Sudan September-November 2012. Int J Infect Dis. 2014;21((Nogodalla) Federal Ministry of Health Khartoum Sudan):259.	Wrong study design
Noiszewska H Niscigorska J. Development of infectious diseases treatment in Szczecin following the World War Two through the prism of infectious diseases ward. Archiwum historii i filozofii medycyny / Polskii Towarzystwo Historii Medycyny i Farmacji. 1996;59(1):103-6.	Non-English
Nosten F Hien TT White NJ. Use of artemisinin derivatives for the control of malaria. Medecine tropicale : revue du Corps de sante colonial. 1998;58(3 Suppl):45-9.	Wrong study design
Nosten F ter Kuile F Chongsuphajaisiddhi T et al. Mefloquine-resistant falciparum malaria on the Thai-Burmese border. Lancet (London England). 1991;337(8750):1140-3.	Wrong study design
Nosten F Van Vugt M Price R et al. Effects of artesunate-mefloquine combination on incidence of Plasmodium falciparum malaria and mefloquine resistance in western Thailand: A prospective study. Lancet. 2000;356(9226):297-302.	Wrong study design
Nsambu MN Bazira L Coulibaly T et al. [Investigation and response to an outbreak of wild poliovirus in Kinshasa]. The Pan African medical journal. 2013;15(101517926):37.	Non-English
Nur YA Groen J Heuvelmans H et al. An outbreak of West Nile fever among migrants in Kisangani Democratic Republic of Congo. The American journal of tropical medicine and hygiene. 1999;61(6):885-8.	Wrong patient populati
Obadare E. A crisis of trust: History politics religion and the polio controversy in Northern Nigeria. Patterns of Prejudice. 2005;39(3):265-84.	Wrong study design
Oberhofer E. Refugee crisis - Migration - Long-distance journeys: Now your knowledge of infectiology is needed! MMW-Fortschritte der Medizin. 2015;157(21-22):10-4.	Non-English
Obol J David Lagoro K Christopher Garimoi O. Knowledge and Misconceptions about Malaria among Pregnant Women in a Post-Conflict Internally Displaced Persons' Camps in Gulu District Northern Uganda. Malaria research and treatment. 2011;2011(101568072):107987.	Wrong study design
O'Brien Dp VSGJSLETSKFLMC. Provision of antiretroviral treatment in conflict settings: the experience of Médecins Sans Frontières. Confl Health. 2010;4:12-0.	Wrong setting

Ocampo CB Mina NJ Carabali M et al. Reduction in dengue cases observed during mass control of Aedes (Stegomyia) in street catch basins in an endemic urban area in Colombia. Acta Trop. 2014;132(0370374):15-22.	Wrong setting
Ochieng RM. Gender and HIV/AIDS education in the multi-cultural context of schools at Kakuma Refugee Camp in Kenya. J Int AIDS Soc. 2012;15((Ochieng) Kenyatta University Educational Foundations Nairobi Kenya):168.	Wrong study design
Ochola E Bayo P Ogwang MD et al. HIV prevalence trend in the conflict to post-conflict transition period in Gulu District Northern Uganda. Sex Transm Infect. 2013;89((Fabiani) Italian National Institute for Health Epidemiology and Communicable Disease Unit	Wrong study design
Okware SI Omaswa F Talisuna A et al. Managing ebola from rural to urban slum settings: Experiences from Uganda. Afr Health Sci. 2015;15(1):312-21.	Wrong study design
Olaseha IO Sridhar MKC. Participatory action research: community diagnosis and intervention in controlling urinary schistosomiasis in an urban community in Ibadan Nigeria. Int Q Community Health Educ. 2005;24(2):153-60.	Wrong study design
O'Laughlin K Kasozi J Rabideau D et al. The cascade of HIV care among refugees and nationals in Nakivale Refugee Settlement in Uganda. HIV Med. 2016((Walensky) Division of Infectious Disease Brigham and Women's Hospital Boston MA USA):no pagination.	Wrong study design
O'Laughlin K Rouhani S Faustin Z et al. Adherence in mobile populations: Qualitative study of ART for refugees in sub-Saharan Africa. J Int Assoc Physicians AIDS Care. 2012;11(6):395.	Wrong study design
O'Laughlin KN Faustin ZM Rouhani SA et al. Increasing HIV testing among African refugees in Africa: Intervening in the daily survival cycle to encourage priority shifting. J Int AIDS Soc. 2012;15((Ware) Brigham and Women's Hospital Boston United States):176-7.	Wrong study design
O'Laughlin KN Kasozi J Bassett IV et al. Predictors of HIV-infection during routine clinic-based HIV testing in Nakivale Refugee settlement in SW Uganda. Annals of Global Health. 2015;81(1):132-3.	Wrong study design
O'Laughlin KN Kasozi J Rabideau DJ et al. The cascade of HIV care among refugees and nationals in Nakivale Refugee Settlement in Uganda. HIV Med. 2017;18(7):513-8.	Wrong study design
O'Laughlin KN Kasozi J Rabideau DJ et al. The refugee cascade of care: Prospective assessment reveals attrition from HIV care in nakivale refugee settlement in Uganda. Ann Emerg Med. 2015;66(4 SUPPL. 1):S78.	Wrong study design
O'Laughlin KN Rabideau DJ Kasozi J et al. Predictors of HIV infection: A prospective HIV screening study in a Ugandan refugee settlement. BMC Infect Dis. 2016;16(1):no pagination.	Wrong study design
Olugasa BO Dogba JB. Mapping of Lassa fever cases in post-conflict Liberia 2008-2012: a descriptive and categorical analysis of age gender and seasonal pattern. Ann Afr Med. 2015;14(2):120-2.	Wrong study design
Oner AF Bay A Arslan S et al. Avian influenza A (H5N1) infection in eastern Turkey in 2006. N Engl J Med. 2006;355(21):2179-85.	Wrong study design
Onyango CO Ofula VO Sang RC et al. Yellow fever outbreak Imatong southern Sudan. Emerg Infect Dis. 2004;10(6):1063-8.	Wrong study design
Ope M Nyoka R Unshur A et al. Evaluation of the field performance of ImmunoCard STAT! rapid diagnostic test for rotavirus in Dadaab refugee camp and at the Kenya-Somalia Border. Am J Trop Med Hyg. 2017;96(6):1302-6.	Wrong study design
Orach CG Dubourg D De Brouwere V. Costs and coverage of reproductive health interventions in three rural refugee-affected districts Uganda. Trop Med Int Health. 2007;12(3):459-69.	Wrong topic

O'Reilly KM et al. The effect of mass immunisation campaigns and new oral poliovirus vaccines on the incidence of poliomyelitis in Pakistan and Afghanistan 2001-11: a retrospective analysis. Lancet. 2012;380:491-8.	Wrong study design
Otshudiema JO Ndakala NG Mawanda ETK et al. Yellow Fever Outbreak - Kongo Central Province Democratic Republic of the Congo August 2016. MMWR Morbidity and mortality weekly report. 2017;66(12):335-8.	Wrong study design
Oucho JO Ama NO. Immigrants' and refugees' unmet reproductive health demands in Botswana: Perceptions of public healthcare providers. South African Family Practice. 2009;51(3):237-43.	Wrong study design
Ozaras R Balkan II Yemisen M. Prejudice and reality about infection risk among Syrian refugees. The Lancet Infectious Diseases. 2016;16(11):1222-3.	Wrong study design
Ozaras R Leblebicioglu H Sunbul M et al. The Syrian conflict and infectious diseases. Expert Rev Anti Infect Ther. 2016;14(6):547-55.	Wrong study design
Page A-L Coldiron ME Gamougam K et al. Four years of case-based surveillance of meningitis following the introduction of MenAfriVac in Moissala Chad: lessons learned. Tropical medicine & international health : TM & IH. 2017;22(12):1561-8.	Wrong study design
Paquet C Hanquet G. Control of infectious diseases in refugee and displaced populations in developing countries. Bulletin de l'Institut Pasteur. 1998;96(1):3-14.	Wrong study design
Paquet C Leborgne P Sasse A et al. [An outbreak of Shigella dysenteriae type 1 dysentery in a refugee camp in Rwanda]. Sante (Montrouge France). 1995;5(3):181-4.	Non-English
Paquet C Leborgne P Sasse A et al. An outbreak of dysentry due to Shigella dysenteriae type 1 in a refugees camp in Rwanda. Cahiers Sante. 1995;5(3):181-4.	Duplicate
Paquet C. Vaccination in emergencies. Vaccine. 1999;17(SUPPL. 3):S116-S9.	Wrong study design
Pasquale H Jarvese M Julla A et al. Malaria control in South Sudan 2006-2013: Strategies progress and challenges. Malar J. 2013;12(1):no pagination.	Wrong study design
Patel S Schechter MT Sewankambo NK et al. Lost in transition: Hiv prevalence and correlates of infection among young people living in post-emergency phase transit camps in gulu district northern uganda. PLoS One. 2014;9(2):no pagination.	Wrong study design
Patel SH Spittal PM Schechter MT et al. Lost in transition: Determining HIV prevalence and related vulnerabilities among young people in post-conflict Northern Uganda. Canadian Journal of Infectious Diseases and Medical Microbiology. 2011;22((Muyinda Sewankambo Kiwanuka) KampalaUganda):24B.	Wrong study design
Pavlovic M Simic D Krstic-Buric M et al. Wartime migration and the incidence of tuberculosis in the Zagreb region Croatia. Eur Respir J. 1998;12(6):1380-3.	Wrong study design
Pelly MD Besse C. Cholera treatment in Goma. Lancet. 1995;345(8964):1567-8.	Wrong study design
Perry RT Plowe CV Koumare B et al. A single dose of live oral cholera vaccine CVD 103-HgR is safe and immunogenic in HIV-infected and HIV-noninfected adults in Mali. Bull World Health Organ. 1998;76(1):63-71.	Wrong setting
Peter KB Ademola AS Oyeku OA. Effects of supplemental measles immunization on cases of measles admitted at the Wesley Guild Hospital Ilesa Nigeria. Afr Health Sci. 2014;14(1):131-5.	Wrong study design
Peterson EA Roberts L Toole MJ et al. The effect of soap distribution on diarrhoea: Nyamithuthu Refugee Camp. Int J Epidemiol. 1998;27(3):520-4.	Extracted in WASH

Pham K Sharpe EC Weiss WM et al. The use of a lot quality assurance sampling methodology to assess and manage primary health interventions in conflictaffected West Darfur Sudan. Population Health Metrics. 2016;14(1):no pagination.	Wrong study design
Phillips CB Patel M. The switch to new conjugated vaccines may compromise immunisation coverage for refugees [1]. Med J Aust. 2006;184(9):473.	Wrong topic
Phillips RM Vujcic J Boscoe A et al. Soap is not enough: handwashing practices and knowledge in refugee camps Maban County South Sudan. Conflict and health. 2015;9(101286573):39.	Wrong study design
Pigeolet M Hababeh M Khader A et al. The effect of continuity of care on antibiotics prescription for Palestinian refugees in UNRWA health centres: A cross-sectional study. The Lancet. 2018;391(SPEC.ISS 1):S19.	Wrong study design
Pinto A Saeed M El Sakka H et al. Setting up an early warning system for epidemic-prone diseases in Darfur: A participative approach. Disasters. 2005;29(4):310-22.	Wrong study design
Pokhrel BM Kubo T. Outbreaks of cholera in Nepal. The Southeast Asian journal of tropical medicine and public health. 1996;27(3):574-9.	Wrong topic
Popal GR. Impact of sanctions on the population of Iraq. Eastern Mediterranean health journal = La revue de sante de la Mediterranee orientale = al-Majallah al-ihhiyah li-sharq al-mutawassi. 2000;6(4):791-5.	Wrong topic
Porter J Kessler C. Tuberculosis in refugees: A neglected dimension of the 'global epidemic of tuberculosis'. Trans R Soc Trop Med Hyg. 1995;89(3):241-2.	Wrong study design
Pottie K Greenaway C Feightner J et al. Evidence-based clinical guidelines for immigrants and refugees. CMAJ. 2011;183(12):E824-E925.	Wrong study design
Pourkarim MR Zandi K Davani NA et al. An aberrant high prevalence of hepatitis B infection among Afghans residing in one of the Bushehr refugee camps (Dalaki camp) in the southwest of Iran. Int J Infect Dis. 2008;12(1):101-2.	Wrong study design
Price R et al. Adverse effects in patients with acute falciparum malaria treated with artemisinin derivatives. Am J Trop Med Hyg. 1999;60(4):547-55.	Wrong study design
Puvacic Z Weinberg J. Impact of war on infectious disease in Bosnia-Hercegovina. Br Med J. 1994;309(6963):1207-8.	Wrong study design
Qayum M Anwar S Raza UA et al. Assessment of health services on relevant primary health care principles in internally displaced people of Pakistan basedon SPHERE standards and indicators. Journal of the College of Physicians and Surgeons Pakistan. 2011;21(5):315-6.	Wrong study design
Quddus A Luby SP Jamal Z et al. Prevalence of hepatitis B among Afghan refugees living in Balochistan Pakistan. Int J Infect Dis. 2006;10(3):242-7.	Wrong study design
Querido J. Emergency initiative to reduce leishmaniasis in Afghanistan. The Lancet infectious diseases. 2004;4(10):599.	Wrong study design
Quick RE Gerber ML Palacios AM et al. Using a knowledge attitudes and practices survey to supplement findings of an outbreak investigation: cholera prevention measures during the 1991 epidemic in Peru. Int J Epidemiol. 1996;25(4):872-8.	Wrong study design
Qurei L Seto D Salah Z et al. A molecular epidemiology survey of respiratory adenoviruses circulating in children residing in Southern Palestine. PLoS One. 2012;7(8):no pagination.	Wrong study design
Raad II Sherertz RJ Russell BA et al. Uncontrolled nosocomial rotavirus transmission during a community outbreak. Am J Infect Control. 1990;18(1):24-8.	Wrong study design

Rajabali A Moin O Ansari AS et al. Communicable disease among displaced Afghans: Refuge without shelter. Nature Reviews Microbiology. 2009;7(8):609-14.	Wrong study design
Ramathal DC Ngassapa OD. Medicinal plants used by Rwandese traditional healers in refugee camps in Tanzania. Pharm Biol. 2001;39(2):132-7.	Wrong study design
Ratho RK Mishra B Singh T et al. Measles outbreak in a migrant population. Indian J Pediatr. 2005;72(10):893-4.	Wrong study design
Rauf A Nadeem MS Ali A et al. Prevalence of hepatitis B and C in internally displaced persons of war against terrorism in Swat Pakistan. Eur J Public Health. 2011;21(5):638-42.	Wrong study design
Ravensbergen SJ Lokate M Cornish D et al. High Prevalence of Infectious Diseases and Drug-Resistant Microorganisms in Asylum Seekers Admitted to Hospital; No Carbapenemase Producing Enterobacteriaceae until September 2015. PLoS One. 2016;11(5):e0154791.	Wrong setting
Refaat MM Mohanna K. Syrian refugees in Lebanon: Facts and solutions. The Lancet. 2013;382(9894):763-4.	Wrong study design
Reichler MR Abbas A Kharabsheh S et al. Outbreak of paralytic poliomyelitis in a highly immunized population in Jordan. The Journal of infectious diseases. 1997;175 Suppl 1(ih3 0413675):S62-70.	Wrong setting
Reisinger EC Grasmug E Krejs GJ. Antibody response after vaccination against typhoid fever in Kurdish refugee camp. Lancet (London England). 1994;343(8902):918-9.	Duplicate
Rey JL Cavallo JD Milleliri JM et al. [Fever of unknown origin (FUO) in the camps of Rwandan refugees in the Goma region of in Zaire (September 1994)]. Bulletin de la Societe de pathologie exotique (1990). 1996;89(3):204-8.	Non-English
Rey JL Milleliri JM Soares JL et al. HIV seropositivity and cholera in refugee children from Rwanda. AIDS (London England). 1995;9(10):1203-4.	Wrong study design
Rey JL. The Bioforce teams in Goma 1994. Medecine de Catastrophe Urgences Collectives. 2000;3(1):36-9.	Non-English
Reyna TM. Observations of a pediatric surgeon in the Persian Gulf War. J Pediatr Surg. 1993;28(2):209-13.	Wrong study design
Riccardo F El Jaish A Shahin Y et al. Warning system in Gaza Strip post conflict 2009. Int J Infect Dis. 2010;14((Pinto) World Health Organization SEARO DSE subunit Bangkok	Wrong topic
Richards C Alonso-Echanove J Caicedo Y et al. Klebsiella pneumoniae bloodstream infections among neonates in a high-risk nursery in Cali Colombia. Infect Control Hosp Epidemiol. 2004;25(3):221-5.	Wrong setting
Rieger M. AIDS and Conflict: Micro Evidence from Burundi. Forum for Health Economics & Policy. 2013;16(1):NA-NA.	Wrong study design
Roberton T Weiss W Oweis A et al. Challenges in estimating vaccine coverage in refugee and displaced populations: Results from household surveys in Jordan and Lebanon. Vaccines. 2017;5(3):22.	Wrong study design
Roberts A Kemp C. Infectious disease. Infectious diseases of refugees and immigrants: dengue fever. J Am Acad Nurse Pract. 2001;13(6):243-5.	Wrong study design
Roberts A Kemp C. Infectious diseases of refugees and immigrants. J Am Acad Nurse Pract. 2001;13(1):7-9.	Wrong study design
Roberts A Kemp C. Infectious diseases of refugees and immigrants. Lassa fever. J Am Acad Nurse Pract. 2002;14(7):289-90.	Wrong study design
Roberts A Kemp C. Infectious diseases of refugees and immigrants: giardiasis (Giardia lamblia). J Am Acad Nurse Pract. 2001;13(12):532-3.	Duplicate

Roberts A Kemp C. Infectious diseases of refugees and immigrants: hookworm. J Am Acad Nurse Pract. 2002;14(5):194.	Wrong study design
Roberts A Kemp C. Infectious diseases. Infectious diseases of refugees and immigrants: giardiasis (Giardia lamblia). J Am Acad Nurse Pract. 2001;13(12):532-3.	Wrong study design
Roberts L Chartier Y Chartier O et al. Keeping clean water clean in a Malawi refugee camp: A randomized intervention trial. Bull World Health Organ. 2001;79(4):280-7.	Extracted in WASH
Roberts L Toole MJ. Cholera deaths in Goma. Lancet (London England). 1995;346(8987):1431.	Wrong study design
Roberts L. Infectious diseases. Polio eradicators struggle to prevent the next outbreak. Science (New York NY). 2014;344(6189):1212-3.	Wrong study design
Robinson C. UNHCR: new strategy for provision of antiretroviral therapy to refugees. HIV/AIDS policy & law review / Canadian HIV/AIDS Legal Network. 2007;12(1):36-8.	Wrong study design
Roggen I van Berlaer G Gijs G et al. Clinical characteristics of the inhabitants of an internally displaced persons camp in Brazzaville Republic of Congo after the arms dump blast on March 4 2012. Prehosp Disaster Med. 2014;29(5):516-20.	Wrong study design
Rojas JC Prieto FE. National immunization day evaluation in Colombia 2001: an ecological approach. Revista de salud publica (Bogota Colombia). 2004;6(1):44-62.	Non-English
Rosen L Steinberg GM. Comments on 'Palestinian refugee conditions associated with intestinal parasites and diarrhoea: Nuseirat refugee camp as a case study' by T.A. Abu Mourad (Public Health 2004; 118:131-42). Public Health (Elsevier). 2004;118(4):307-9.	Wrong study design
Rotavirus infections subject of CDC study vaccine trials. Hosp Infect Control. 1989;16(8):97-100.	Wrong setting
Rotavirus vaccines. Wkly Epidemiol Rec. 1999;74(5):33-.	Wrong study design
Rouhani SA O'Laughlin KN Faustin ZM et al. The role of social support on HIV testing and treatment adherence: A qualitative study of HIV-infected refugees in southwestern Uganda. Global Public Health. 2016((Ware) Department of Global Health and Social Medicine Harvard Medical School Boston	Wrong study design
Rouhani SA O'Laughlin KN Faustin ZM et al. The role of social support on HIV testing and treatment adherence: A qualitative study of HIV-infected refugees in southwestern Uganda. Global Public Health. 2017;12(8):1051-64.	Wrong study design
Rowland M Hewitt S Durrani N et al. Transmission and control of vivax malaria in Afghan refugee settlements in Pakistan. Trans R Soc Trop Med Hyg. 1997;91(3):252-5.	Wrong study design
Rowland M Munir A Durrani N et al. An outbreak of cutaneous leishmaniasis in an Afghan refugee settlement in north-west Pakistan. Trans R Soc Trop Med Hyg. 1999;93(2):133-6.	Wrong study design
Rowland M Nosten F. Malaria epidemiology and control in refugee camps and complex emergencies. Ann Trop Med Parasitol. 2001;95(8):741-54.	Wrong study design
Rowland M Rab MA Freeman T et al. Afghan refugees and the temporal and spatial distribution of malaria in Pakistan. Soc Sci Med. 2002;55(11):2061-72.	Wrong study design
Rowland M. Malaria control in Afghan refugee camps: Novel solutions. Trans R Soc Trop Med Hyg. 2001;95(2):125-6.	Duplicate
Rowland M. Malaria control in the Afghan refugee camps of western Pakistan. Trans R Soc Trop Med Hyg. 1999;93(5):458-9.	Wrong study design
Rowland M. Malaria control: bednets or spraying? Malaria control in the Afghan refugee camps of western Pakistan. Trans R Soc Trop Med Hyg. 1999;93(5):458-9.	Duplicate
Ruef C. Infections in refugees. Infection. 2003;31(2):69.	Wrong study design

Ruiz E Puges X Artigues J et al. [Health intervention in an Albanian-Kosovar refugees camp]. Gac Sanit. 2001;15(4):356-8.	Non-English
Saab MW Hoteit R Shammaa D et al. Diagnosis of cutaneous leishmaniasis: Why punch when you can scrape? Lab Invest. 2014;94((Saab Hoteit Shammaa	Wrong study design
Sachdeva A Kukreja S Jain V et al. Meningococcal disease--outbreak in Delhi. Indian Pediatr. 2005;42(6):547-56.	Wrong study design
Sack DA Naficy A Rao MR et al. Cholera vaccine in refugee settings [3] (multiple letters). J Am Med Assoc. 1998;280(7):600-2.	Wrong study design
Saeed IE Ahmed ES. Determinants of malaria mortality among displaced people in Khartoum state Sudan. Eastern Mediterranean Health Journal. 2003;9(4):593-600.	Wrong study design
Safi N Davis GD Nadir M et al. Evaluation of thermotherapy for the treatment of cutaneous leishmaniasis in Kabul Afghanistan: a randomized controlled trial. Mil Med. 2012;177(3):345-51.	Wrong setting
Saks E Tanabe K Garcia C et al. HPV vaccination of adolescents in a dedicated refugee clinic. Gynecol Oncol. 2017;145(Supplement 1):185-6.	Wrong study design
Salama P Dondero TJ. HIV surveillance in complex emergencies. AIDS (London England). 2001;15 Suppl 3(aid 8710219):S4-12.	Wrong study design
Salloum T Khalifeh I Tokajian S. Detection molecular typing and phylogenetic analysis of Leishmania isolated from cases of leishmaniasis among Syrian refugees in Lebanon. Parasite Epidemiology and Control. 2016;1(2):159-68.	Wrong study design
Salman IS Vural A Unver A et al. Cutaneous leishmaniasis cases in Nizip Turkey after the Syrian civil war. Mikrobiyol Bul. 2014;48(1):106-13.	Non-English
Santantonio T Lo Caputo S Germinario C et al. Prevalence of hepatitis virus infections in Albanian refugees. Eur J Epidemiol. 1993;9(5):537-40.	Wrong study design
Satti GMSHE Ibrahim SA. The efficacy of artemether versus quinine in the treatment of cerebral malaria. J Egypt Soc Parasitol. 2002;32(2):611-23.	Wrong study design
Schimmer B Ihekweazu C. Polio eradication and measles immunisation in Nigeria. The Lancet Infectious diseases. 2006;6(2):63-5.	Wrong setting
Schluterman N. Adherence and safety of the hpv vaccine in bamako mali. Dissertation Abstracts International: Section B: The Sciences and Engineering. 2015;76(6-B(E)):No-Specified.	Wrong study design
Schwartz J. Screening tests for parasites in refugees...Am Fam Physician. 2011 Feb 15;83(4):429-36. Am Fam Physician. 2012;86(7):596-8.	Wrong study design
Scobie H Phares C Taylor E et al. Knowledge attitudes and practices regarding cholera safe water	Wrong study design
Scobie HM Phares CR Wannemuehler KA et al. Correction: Use of Oral Cholera Vaccine and Knowledge Attitudes and Practices Regarding Safe Water	Wrong study design
Scobie HM Phares CR Wannemuehler KA et al. Use of Oral Cholera Vaccine and Knowledge Attitudes and Practices Regarding Safe Water	Wrong topic
Senessie C Gage GN von Elm E. Delays in childhood immunization in a conflict area: a study from Sierra Leone during civil war. Conflict and health. 2007;1(101286573):14.	Wrong study design
Seufi AM Galal FH. Role of Culex and Anopheles mosquito species as potential vectors of rift valley fever virus in Sudan outbreak 2007. BMC Infect Dis. 2010;10(1):65-.	Wrong study design

Shah I Rowland M Mehmood P et al. Chloroquine resistance in Pakistan and the upsurge of falciparum malaria in Pakistani and Afghan refugee populations. Ann Trop Med Parasitol. 1997;91(6):591-602.	Wrong study design
Shahandeh K Basseri HR Sharifzadeh Y. An application of cultural model to assess and compare malaria prevention among Afghani migrant and Baluchi resident in the endemic area southeastern Iran. Journal of immigrant and minority health / Center for Minority Public Health. 2014;16(1):102-10.	Wrong study design
Sheikh MA Makokha F Hussein AM et al. Combined use of inactivated and oral poliovirus vaccines in refugee camps and surrounding communities - Kenya December 2013. MMWR Morbidity and mortality weekly report. 2014;63(11):237-41.	Duplicate
Shikanga OT Mutonga D Abade M et al. High mortality in a cholera outbreak in western Kenya after post-election violence in 2008. The American journal of tropical medicine and hygiene. 2009;81(6):1085-90.	Wrong study design
Shimakawa Y Camelique O Ariyoshi K. Outbreak of chickenpox in a refugee camp of northern Thailand. Conflict and health. 2010;4(101286573):4.	Wrong study design
Shook G Fos P. An environmental health evaulation tool for locating and assessing disaster relief and refugee camps. J Environ Health. 1993;55(7):21-3.	Wrong study design
Siddique AK. Failure of treatment centres to prevent cholera deaths in Goma. Lancet (London England). 1995;346(8971):379.	Wrong study design
Siegel B. Meningitis outbreak: Mother Nature sends a scary message. Med Econ. 1995;72(16):173-passim.	Wrong study design
Simanjuntak CH Larasati W Arjoso S et al. Cholera in Indonesia in 1993-1999. The American journal of tropical medicine and hygiene. 2001;65(6):788-97.	Wrong study design
Simetka O Reilley B Joseph M et al. Obstetrics during Civil War: six months on a maternity ward in Mallavi northern Sri Lanka. Medicine conflict	Wrong setting
Singh J Gupta RS Bora D et al. Epidemiologic consequences of moderate coverage levels of measles vaccine in a district headquarter town (Alwar) in India 1996. J Trop Pediatr. 1998;44(6):369-71.	Wrong setting
Singh NP Jhamb R Agarwal SK et al. The 2003 outbreak of dengue fever in Delhi India. Southeast Asian J Trop Med Public Health. 2005;36(5):1174-8.	Wrong setting
Sinlaparatsamee S Nuniem J Kankao J et al. An outbreak of hepatitis A in school children at Nakhon Si Thammarat southern Thailand. The Southeast Asian journal of tropical medicine and public health. 1995;26(1):104-8.	Wrong setting
Sirajul Islam M Siddique AKM Salam A et al. Microbiological investigation of diarrhoea epidemics among Rwandan refugees in Zaire. Trans R Soc Trop Med Hyg. 1995;89(5):506.	Wrong study design
Slutsker L Tipple M Keane V et al. Malaria in East African refugees resettling to the United States: Development of strategies to reduce the risk of imported malaria. J Infect Dis. 1995;171(2):489-93.	Wrong setting
Smith M Lo W Bindra J. Prescribing for refugees. Australian Prescriber. 2013;36(5):146-7.	Wrong setting
Soares JL Milleliri JM Pigny N et al. Efficacy of bacillare dysentery's treatment by lomefloxacine amongst Rwandese refugees in North Zaire [5]. Med Mal Infect. 1996;26(2):141-4.	Non-English
Somerville MA. The case against HIV antibody testing of refugees and immigrants (I: Reply). CMAJ. 1990;143(3):172-3.	Wrong study design
Soydan L Demir AA Tunaci A. Frequency of abnormal pulmonary computed tomography findings in asylum seeking refugees in Turkey. International Health. 2017;9(2):118-23.	Wrong setting

Meteke S, *et al. BMJ Global Health* 2020; 5:e001967. doi: 10.1136/bmjgh-2019-001967

Spiegel A Greindl Y Lippeveld T et al. [Effect of 2 vaccination strategies on developments during the epidemic of meningococcal A meningitis in N'Djamena (Chad) in 1988]. Bull World Health Organ. 1993;71(3-4):311-5.	Non-English
Spiegel P Hering H Paik E et al. Conflict-affected displaced persons need to benefit more from HIV Global Fund grants and national strategic plans. J Int AIDS Soc. 2012;15((Paik) Independent Consultant Paris France):284.	Wrong study design
Spiegel P Sheik M Gotway-Crawford C et al. Health programmes and policies associated with decreased mortality in displaced people in postemergency phase camps: a retrospective study. Lancet. 2002;360 North American Edition(9349):1927-34.	Wrong study design
Spiegel P Sheik M Gotway-Crawford C et al. Health programmes and policies associated with decreased mortality in displaced people in postemergency phase camps: A retrospective study. Lancet. 2002;360(9349):1927-34.	Duplicate
Spiegel PB Hering H Paik E et al. Conflict-affected displaced persons need to benefit more from HIV and malaria national strategic plans and Global Fund grants. Conflict and health. 2010;4(101286573):2.	Wrong study design
Spiegel PB. HIV/AIDS among conflict-affected and displaced populations: Dispelling myths and taking action. Disasters. 2004;28(3):322-39.	Wrong study design
Stadnichenko AV Bazdyrev VP Voronenko NV et al. A cholera outbreak in Nikolaev Province. Likars'ka sprava / Ministerstvo okhorony zdorov'ia Ukrainy. 1992(11-12):107.	Duplicate
Steele A Clarke B Watkins O. Impact of jerry can disinfection in a camp environment - experiences in an IDP camp in Northern Uganda. Journal of water and health. 2008;6(4):559-64.	Wrong topic
Stephen H Roberts B. A case study of the provision of antiretroviral therapy for refugees in Tanzania. Medicine conflict and survival. 2009;25(2):134-47.	Wrong patient populati
Stevens JC Reilley B Hargreaves S et al. A year in Afghanistan - Herat hospital paediatric ward. Br J Gen Pract. 2004;54(507):794-5.	Wrong study design
Sullivan P. Poor conditions in refugee camps make malaria screening difficult: Expert. CMAJ. 2000;163(8):1036.	Wrong setting
Sutter RW Haefliger E. Tuberculosis morbidity and infection in Vietnamese in Southeast Asian refugee camps. Am Rev Respir Dis. 1990;141(6):1483-6.	Wrong study design
Swaddiwudhipong W Ngamsaithong C Peanumlom P et al. An outbreak of cholera among migrants living in a Thai-Myanmar border area. Journal of the Medical Association of Thailand = Chotmaihet thangphaet. 2008;91(9):1433-40.	Wrong patient populati
Swanson SJ Phares CR Mamo B et al. Albendazole therapy and enteric parasites in United States-bound refugees. N Engl J Med. 2012;366(16):1498-507.	Wrong setting
Swerdlow DL Levine O Toole MJ et al. Cholera control among Rwandan refugees in Zaire. Lancet (London England). 1994;344(8932):1302-3.	Duplicate
Swerdlow DL Levine O. Cholera control among Rwandan refugees in Zaire [19]. Lancet. 1994;344(8932):1302-3.	Wrong study design
Tabbaa D Seimenis A. Population displacements as a risk factor for the emergence of epidemics. Vet Ital. 2013;49(1):19-23.	Wrong study design
Tangermann RH Hull HF Jafari H et al. Eradication of poliomyelitis in countries affected by conflict. Bull World Health Organ. 2000;78(3):330-8.	Wrong study design

ter Kuile FO et al. Mefloquine treatment of acute falciparum malaria: a prospective study of non-serious adverse effects in 3673 patients. Bull World Health Organ. 1995;73(5):631-42.	Wrong study design
Terasaki G Ahrenholz NC Haider MZ. Care of Adult Refugees with Chronic Conditions. Med Clin North Am. 2015;99(5):1039-58.	Wrong study design
Thonnon J Chauvancy G. Evaluation of the immunological and entomological indices of yellow fever in the subprefecture of Tai Ivory Coast. Bulletin de la Societe de pathologie exotique (1990). 1994;87(1):7-10.	Non-English
Tigani B. The trends of tuberculosis in Kosovo in the post war period (20012010). Eur Respir J. 2012;40:no pagination.	Wrong setting
Tiwari SK Love EJ. Gender and tuberculosis control in armed conflict areas in Nepal. International Medical Journal. 2007;14(4):265-71.	Wrong study design
Toole MJ Waldman RJ. Refugees and displaced persons: War hunger and public health. J Am Med Assoc. 1993;270(5):600-5.	Wrong study design
Treatment: HIV treatment regimens can benefit refugees. AIDS Policy Law. 2006;21(17):1-.	Duplicate
Tschirhart N Nosten F Foster AM. Access to free or low-cost tuberculosis treatment for migrants and refugees along the Thailand-Myanmar border in Tak province Thailand. International Journal for Equity in Health. 2016;15(1):no pagination.	Duplicate
Tschirhart N Nosten F Foster AM. Access to free or low-cost tuberculosis treatment for migrants and refugees along the Thailand-Myanmar border in Tak province Thailand. International Journal for Equity in Health. 2016;16:1-12.	Wrong study design
Tschirhart N Sein T Nosten F et al. Migrant and Refugee patient perspectives on travel and tuberculosis along the Thailand-Myanmar Border: A qualitative study. PLoS One. 2016;11(8):no pagination.	Wrong study design
Tsoka-Gwegweni J Okafor U. Haematological alterations in malaria-infected refugees in South Africa. Malar J. 2014;13((Tsoka-Gwegweni Okafor) University of KwaZulu-Natal Durban KwaZulu-Natal South Africa):S37.	Wrong study design
Tsoka-Gwegweni JM Okafor U. Asymptomatic malaria in refugees living in a non-endemic South African City. PLoS One. 2014;9(9):no pagination.	Wrong study design
Tulchinsky TH Belmaker I Raabi S et al. Measles during the Gulf War: a public health threat in Israel the West Bank and Gaza. Public Health Rev. 1992;20(3-4):285-96.	Wrong study design
Tulchinsky TH Belmaker I Raabi S et al. Measles during the Gulf War: A public health threat in Israel the West Bank and Gaza. Public Health Rev. 1993;20(3-4):285-96.	Duplicate
Turktan M Ak O Erdem H et al. Community acquired infections among refugees leading to Intensive Care Unit admissions in Turkey. Int J Infect Dis. 2017;58((Rello) Centro de Investigacion Biomedica en Red de Enfermedades Respiratorias (CIBERES) Universitat Autonoma de Barcelona Spain):111-4.	Wrong study design
Turner C Turner P Cararra V et al. A High Burden of Respiratory Syncytial Virus Associated Pneumonia in Children Less than Two Years of Age in a South East Asian Refugee Population. PLoS One. 2012;7(11):no pagination.	Wrong study design
Turner C Turner P Cararra V et al. The epidemiology of pneumonia in a birth cohort of children living on the Thai-Myanmar border. Int J Infect Dis. 2012;16((Goldblatt) Institute of Child Health London United Kingdom):e13.	Wrong study design

Turner C Turner P Carrara V et al. High Rates of Pneumonia in Children under Two Years of Age in a South East Asian Refugee Population. PLoS One. 2013;8(1):no pagination.	Wrong study design
Turner C Turner P Po L et al. Group B streptococcal carriage serotype distribution and antibiotic susceptibilities in pregnant women at the time of delivery in a refugee population on the Thai-Myanmar border. BMC Infect Dis. 2012;12((Heath) St George's University of London	Wrong study design
Turner P Carrara V Turner C et al. Respiratory virus surveillance in hospitalized pneumonia patients on the Thailand-Myanmar border. Int J Infect Dis. 2012;16((Phares Ortega) Thailand Ministry of Public Health US Centers for Disease Control and Prevention Collaboration	Wrong study design
Turner P Turner CL Watthanaworawit W et al. Influenza in refugees on the Thailand-Myanmar border May-October 2009. Emerg Infect Dis. 2010;16(9):1366-72.	Wrong study design
Turpie ID. Tuberculosis in Somalia. Scott Med J. 2008;53(2):7-8.	Wrong study design
Ujjiga TTA Wamala JF Mogga JJH et al. Risk Factors for Sustained Cholera Transmission Juba County South Sudan	Wrong study design
Ul Haq KA Gul NA Muhammad Hammad H et al. Prevalence of giardia intestinalis and hymenolepis nana in afghan refugee population of mianwali district pakistan. Afr Health Sci. 2015;15(2):394-400.	Wrong study design
Umubyeyi AN Vandebriel G Gasana M et al. Results of a national survey on drug resistance among pulmonary tuberculosis patients in Rwanda. Int J Tuberc Lung Dis. 2007;11(2):189-94.	Wrong setting
Usmanov I Favorov MO Chorba TL. Universal immunization: The diphtheria control strategy of choice in the Republic of Tajikistan 1993-1997. J Infect Dis. 2000;181(SUPPL. 1):S86-S93.	Wrong study design
Valadez JJ Berendes S Jeffery C et al. Filling the Knowledge Gap: Measuring HIV Prevalence and Risk Factors among Men Who Have Sex with Men and Female Sex Workers in Tripoli Libya. PLoS One. 2013;8(6):no pagination.	Wrong topic
Valenciano M Coulombier D Lopes Cardozo B et al. Challenges for communicable disease surveillance and control in southern Iraq April-June 2003. Chicago Illinois: American Medical Association; 2003. p. 654-8.	Wrong study design
Valero-Bernal MV. Malaria in Colombia: Retrospective glance during the past 40 years. Revista de Salud Publica. 2006;8(3):141-9.	Wrong study design
Van Berlaer G Elsafti AM Al Safadi M et al. Diagnoses infections and injuries in Northern Syrian children during the civil war: A cross-sectional study. PLoS One. 2017;12(9):e0182770.	Wrong study design
Varaine F Caugant DA Riou JY et al. Meningitis outbreaks and vaccination strategy. Trans R Soc Trop Med Hyg. 1997;91(1):3-7.	Wrong setting
Verma R Khanna P Chawla S. Cholera vaccine: New preventive tool for endemic countries. Human Vaccines and Immunotherapeutics. 2012;8(5):682-4.	Wrong study design
Vignier N Jeannerod V Pires V et al. PADS 2-05 - Medical care for Syrian and Iraqi refugees with infectious diseases. Med Mal Infect. 2016;46(4):93.	Non-English
Vong S Kebela BO Mukinda V et al. An outbreak of "clinically defined" pertussis in the Democratic Republic of Congo. Medecine tropicale : revue du Corps de sante colonial. 2002;62(1):99.	Non-English
Voorman A Hoff NA Doshi RH et al. Polio immunity and the impact of mass immunization campaigns in the Democratic Republic of the Congo. Vaccine. 2017;35(42):5693-9.	Wrong study design
Vuolo E. Equity in cutaneous leishmaniasis treatment access: Challenges and opportunities from Kabul Afghanistan. Am J Trop Med Hyg. 2010;83(5 SUPPL. 1):125.	Wrong study design

Wagner Z Heft-Neal S Wise PH et al. Women and children living in areas of armed conflict in Africa: a geospatial analysis of mortality and orphanhood. The Lancet Global Health. 2019;7(12):e1622-e31.	Wrong study design
Wahid S Drakeley C Corran P et al. Markers of infection and exposure to malaria in Afghan refugee camps in Khyber Pukhtoon-Khwa (KPK) Pakistan. Am J Trop Med Hyg. 2011;85(6 SUPPL. 1):276.	Wrong study design
Wahid S Stresman GH Kamal SS et al. Heterogeneous malaria transmission in long-term Afghan refugee populations: A cross-sectional study in five refugee camps in northern Pakistan. Malar J. 2016;15(1):no pagination.	Wrong study design
Wakabi W. South Sudan faces grim health and humanitarian situation. The Lancet. 2011;377(9784):2167-8.	Wrong study design
Waldman RJ. Cholera vaccination in refugee settings. J Am Med Assoc. 1998;279(7):552-3.	Wrong study design
Walsh M Min AK Lwin ST et al. Vulnerable populations: Chronically ill individuals people living with HIV women with reproductive health concerns	Wrong study design
Wares DF et al. Control of tuberculosis amongst the Tibetan refugee community in northern India. Indian J Tuberc. 2000;47(1):35-41.	Wrong study design
Warraich H. Pakistan: The final frontier for a polio-free world. The Lancet. 2011;377(9761):207-8.	Wrong study design
White AL Min TH Gross MM et al. Accelerated training of skilled birth attendants in a marginalized population on the Thai-myanmar border: A multiple methods program evaluation. PLoS One. 2016;11(10):no pagination.	Wrong study design
Wolff SP. Leukaemia and wartime evacuation. Nature. 1991;349(6304):23.	Wrong study design
World Health O. Poliomyelitis outbreak spreads across Yemen; case confirmed in Indonesia. Releve epidemiologique hebdomadaire. 2005;80(18):157-8. Ford H. Another African disaster. BMJ (Clinical research ed). 1992;305(6867):1479-80.	Wrong study design
World health roundup. Am J Nurs. 2005;105(6):22-.	Wrong study design
World health roundup. Am J Nurs. 2009;109(5):20-.	Wrong topic
Wolff SP. Leukaemia and wartime evacuation. Nature. 1991;349(6304):23. Wessely S. Commentary: What interventions work for victims of conflict related rape? BMJ (Online). 2010;341(7785):1253.	Duplicate
Woodhead C, Wessely S. Commentary: What interventions work for victims of conflict related rape? BMJ (Online). 2010;341(7785):1253.	Wrong study design
Woods, C W; Armstrong, G; Sackey So et al. . Emergency vaccination against epidemic meningitis in Ghana: implications for the control of meningococcal disease in West Africa. Lancet (London England). 2000;355(9197):30-3	Wrong setting
World Health O. Recent news from WHO. Bull World Health Organ. 2010;88(9):649-.	Wrong setting
World Health O. Rohingya and host communities receive cholera vaccine. Bull World Health Organ. 2017;95(11):732-.	Wrong study design
World Health Organization. Measles vaccination in Nigeria. Bull World Health Organ. 2017;95(2):88-.	Wrong study design
Wright J Kut A Yaman A et al. Health problems among UN refugees at a family medical centre in Ankara Turkey. Scand J Prim Health Care. 2002;20(2):85-7.	Wrong study design
Yaman H Perry RT Yameogo A et al. Migration as a risk factor for measles after a mass vaccination campaign Burkina Faso 2002. Int J Epidemiol. 2005;34(3):556-64.	Wrong study design
Yameogo KR Aksoy M Simsek Z et al. [Investigation of the prevalence of Trichomonas vaginalis among female Syrian refugees with the complaints of vaginitis aged between 15-49 years]. Mikrobiyol Bul. 2016;50(4):590-7.	Wrong setting

Yentur Doni N Watt GCM O'Donnell CA. Living and health conditions of Palestinian refugees in an unofficial camp in the Lebanon: A cross-sectional survey. J Epidemiol Community Health. 2008;62(2):91-7.	Wrong study design
Zabaneh JE, Watt GCM, O'Donnell CA. Living and health conditions of Palestinian refugees in an unofficial camp in the Lebanon: A cross-sectional survey. J Epidemiol Community Health. 2008;62(2):91-7.	Wrong topic
Zapor MJ, Moran KA. Infectious diseases during wartime. Curr Opin Infect Dis. 2005;18(5):395-9.	Wrong study design
Zarocostas J. UN intensifies relief efforts as Somali famine is predicted to spread. BMJ (Clinical research ed). 2011;343:d4949. Lo E Zhong D et al. Impact of interventions on malaria in internally displaced persons along the China-Myanmar border: 2011-2014. Malar J. 2016;15(1):no pagination.	Wrong study design
Zhou G ???. The Knowledge & Attitude on Tuberculosis by Parents of North Korean Refugee Children. Child Health Nursing Research. 2015;21(3):216-26.	Wrong study design
